# Biological characteristics of aging in human acute myeloid leukemia cells: the possible importance of aldehyde dehydrogenase, the cytoskeleton and altered transcriptional regulation

**DOI:** 10.18632/aging.202361

**Published:** 2020-12-20

**Authors:** Maria Hernandez-Valladares, Elise Aasebø, Frode Berven, Frode Selheim, Øystein Bruserud

**Affiliations:** 1 Department of Clinical Science, University of Bergen, Bergen 5021, Norway; 2 The Proteomics Facility of the University of Bergen (PROBE), University of Bergen, Bergen 5009, Norway; 3 The Department of Biomedicine, University of Bergen, Bergen 5009, Norway

**Keywords:** acute myeloid leukenia, age, risk, ALDH2, cytogenetics

## Abstract

Patients with acute myeloid leukemia (AML) have a median age of 65-70 years at diagnosis. Elderly patients have more chemoresistant disease, and this is partly due to decreased frequencies of favorable and increased frequencies of adverse genetic abnormalities. However, aging-dependent differences may also contribute. We therefore compared AML cell proteomic and phosphoproteomic profiles for (i) elderly low-risk and younger low-risk patients with favorable genetic abnormalities; and (ii) high-risk patients with adverse genetic abnormalities and a higher median age against all low-risk patients with lower median age. Elderly low-risk and younger low-risk patients showed mainly phosphoproteomic differences especially involving transcriptional regulators and cytoskeleton. When comparing high-risk and low-risk patients both proteomic and phosphoproteomic studies showed differences involving cytoskeleton and immunoregulation but also transcriptional regulation and cell division. The age-associated prognostic impact of cyclin-dependent kinases was dependent on the cellular context. The protein level of the adverse prognostic biomarker mitochondrial aldehyde dehydrogenase (ALDH2) showed a similar significant upregulation both in elderly low-risk and elderly high-risk patients. Our results suggest that molecular mechanisms associated with cellular aging influence chemoresistance of AML cells, and especially the cytoskeleton function may then influence cellular hallmarks of aging, e.g. mitosis, polarity, intracellular transport and adhesion.

## INTRODUCTION

Acute myeloid leukemia (AML) is an aggressive malignancy characterized by accumulation of immature myeloid cells in the bone marrow [1, 2]. There are two main subsets of AML. The minority of patient with the acute promyelocytic leukemia (APL) variant are characterized by specific genetic abnormalities, accumulation of immature promyelocytic cells, a clinical picture including severe coagulopathy, specific treatment and relatively good prognosis even for elderly patients [3]. In contrast, the non-APL variants of the disease are usually characterized by accumulation of immature blast cells in the bone marrow, it is very heterogeneous with regard to genetic abnormalities and elderly patients often have a more chemoresistant disease and thereby an adverse prognosis compared with younger patients [4]. All patients in the present study have non-APL disease (referred to as AML in our article).

The median age at the first time of AML diagnosis is 65-70 year [1]; the elderly patients with chemoresistant disease thus constitute a large subset of patients. The chemoresistance and thereby the adverse prognosis even when receiving the most intensive treatment is probably caused by several factors. First, favorable cytogenetic abnormalities are less frequent in elderly patients [1, 4]. Second, a relatively large subgroup of these patients have secondary AML (i.e. secondary to previous cytotoxic therapy or a less aggressive chronic hematological malignancy) that can be associated with an adverse prognosis [4], although the independent prognostic impact of this factor in elderly AML patients has recently been questioned [5]. Third, cytogenetic as well as molecular genetic abnormalities (e.g. DNA (cytosine-5)-methyltransferase 3A, *DNMT3A*, and polycomb group protein ASXL1, *ASXL1,* mutations) with adverse prognostic impact are also more frequent in elderly patients [1, 4, 6]. However, additional aging-dependent abnormalities are probably also important for the adverse prognosis of elderly patients. The hematopoietic stem cells in elderly differ from the stem cell in younger individuals with regard to accumulation of mutations and increased frequency of clonal hematopoiesis that seems to predispose to later AML [7]. Age-associated epigenetic changes have also been described [7], and age-associated changes in the bone marrow microenvironment may preferentially support the expansion of cells with preleukemic characteristics [8–12]. Finally, aging hematopoietic stem cells are also characterized by increased numbers of mitochondria, metabolic alterations with decreased autophagy, nuclear abnormalities with decreased levels of lamin (LMNA) in the nuclear envelope and altered cellular polarity with differences in the intracellular distribution of important biomolecules [13–20]. A previous mRNA microarray study suggested that aged AML cells differed in their expression of certain mediators such as RAS, tyrosine-protein kinase Src (SRC) and tumor necrosis factor (TNF) [21].

Taken together all the observations described above suggest that several mechanisms contribute to the chemoresistance of many elderly AML patients. These mechanisms include factors that have a generally accepted prognostic impact independent of age but with age-dependent differences in their frequency. However, additional biological factors that become more frequent with aging are probably also important, and these last observations have led to the hypothesis that aging contributes to leukemogenesis [7]. It is not known whether similar aging-associated characteristics also contribute to the chemoresistance of elderly patients.

To further elucidate possible molecular mechanisms that contributes to chemoresistance especially in elderly individuals we compared the liquid chromatography tandem mass spectrometry (LC-MS/MS)-generated proteomic and phophoproteomic profiles of AML cells derived from contrasting patient groups: (i) elderly low-risk (median age of 68 years) *vs* younger low-risk patients (median age of 47 years) with favorable genetic abnormalities; and (ii) high-risk patients (median age of 74 years) with adverse genetic abnormalities *vs* all the low-risk patients (median age of 64.5 years) [1]. Both these comparisons demonstrated high aldehyde dehydrogenase (ALDH2) levels, altered expression of cytoskeletal proteins and an altered transcriptional regulation in AML cells derived from elderly patients.

## RESULTS

### Patients included in the study

Based on the AML cell samples collected at the first time of diagnosis and the predefined genetic criteria we selected 18 low-risk patients with favorable genetic prognosis and 15 high-risk patients with adverse genetic prognosis (see Materials and Methods for group definitions; Tables 1, 2 and Supplementary Tables 1, 2). In our present context the terms high- and low-risk refer to the risk of having a chemoresistant relapse even after the most intensive antileukemic therapy. The two groups differed significantly with regard to age, cause of their leukemia and morphological signs of differentiation. Low-risk patients were generally younger, none of them had secondary AML and half of them had normal karyotype (Tables 1, 2). The frequency of patients with monocytic AML cell differentiation (i.e. FAB M4/5) was higher in the low-risk group. However, the expression of the CD34 stem cell marker did not differ significantly between the two groups. Most high-risk patients were older and had complex cytogenetic abnormalities (i.e. ≥3 abnormalities).

**Table 1:** Characteristics of the AML patient cohort of this study.

	**High-risk patient group**	**Low-risk patient group**	***P* value**
Number of patients	15	18	
Median age (range), years	74 (50-87)	64.5 (33-79)	0.005
Sex (males/females)	10/5	10/8	NS
Secondary AML cases	6	0	0.005
Chemotherapy predisposing to later AML	1	0	
Previous hematological disease	5	0	
Signs of differentiation			
FAB M4/M5 (monocytic differentiation)	3	11	0.039
CD34 expression	12	10	NS
Adverse genetic abnormalities			
Complex karyotype	10		
Monosomal karyotype	1		
del 5, del 12, -7	4		
Favorable genetic abnormalities			
inv16, t(16;16)		4	
t(8;21)		5	
Normal karyotype, *FLT3* WT or low ITD ratio, *NPM1*-INS		8	
Normal karyotype, *FLT3* WT, *NPM1* WT, *CEBPA* mutated		1	

FAB, French-American-British; WT, wild-type; ITD, internal tandem duplication; INS, a 4 bp-insertion/duplication; NS, not significant.

**Table 2:** Characteristics of the elderly low-risk and younger low-risk patient subgroups based on an age threshold of 65 years.

	**Elderly low-risk patient group**	**Younger low-risk patient group**	***P* value**
Number of patients	9	9	
Median age (range), years	68 (66-79)	47 (33-64)	<0.0001
Sex (males/females)	3/6	7/2	NS
Signs of differentiation			
FAB M4/M5 (monocytic differentiation)	7	4	NS
CD34 expression	5	5	NS
Favorable genetic abnormalities			
inv16, t(16;16)	3	1	NS
t(8;21)	2	3	NS
Normal karyotype, *FLT3* WT or low ITD ratio, *NPM1*-INS	4	4	NS
Normal karyotype, *FLT3* WT, *NPM1* WT, *CEBPA* mutated	0	1	NS

FAB, French-American-British; WT, wild-type; ITD, internal tandem duplication; INS, a 4 bp-insertion/duplication; NS, not significant.

In order to study the impact of morphological signs of differentiation in patient groups that differed significantly with regard to age, we carried out proliferation assays with primary AML cells derived from an alternative cohort of consecutive patients in the presence of hematopoietic growth factors (Supplementary Analysis Tables 1, 2). Our results showed that the proliferative responsiveness of patients with or without morphological signs of differentiation and with age above or below 65 years did not differ significantly. Thus, based on this analysis that included consecutive patients, i.e. not only high-/low-risk karyotypes but also normal and intermediate risk karyotype, we could not find any evidence for a general association between proliferative capacity and patient age/AML cell differentiation.

The 33 AML patient samples were used to perform MS-based proteomics and phosphoproteomics analyses as follows. Firstly, we have carried out an age-dependent analysis in low-risk patients and compared the proteomic and phosphoproteomic profiles between nine elderly low-risk and nine younger low-risk (see Materials and Methods for subgroup definitions). Secondly, we have investigated whether the identified age-dependent differences were also observed when comparing high-risk and all the low-risk patients that differed in age and cytogenetics-related prognosis.

### Effects of aging on the AML cell proteome; a proteomic comparison between elderly and younger low-risk patients

**Figure 1:**
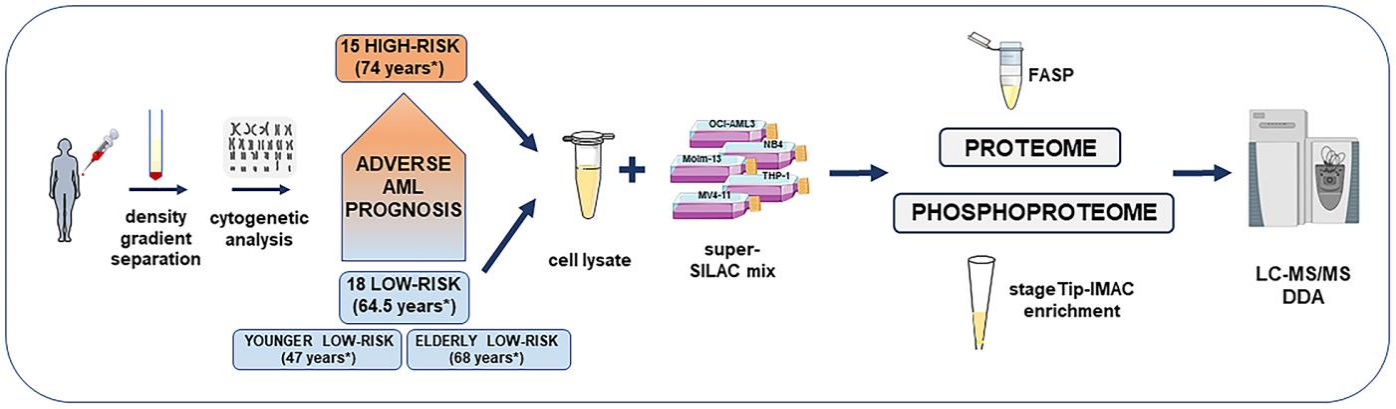
**Overview of the high-risk and low-risk AML patient cohort and the liquid chromatography tandem mass spectrometry (LC-MS/MS) workflow for the proteome and phosphoproteome analysis.** The study included AML cell samples from 15 high-risk and 18 low-risk patients collected at the time of first diagnosis. Patients were classified after cytogenetic and molecular genetic analyses. Low-risk patients were further split into elderly low-risk and younger low-risk patients. AML sample preparation steps for proteome and phosphoproteome analysis included AML cell enrichment by density gradient separation, genetic analyses with classification of patients, cell lysis, addition of the super-SILAC (stable isotope labeling with amino acids in cell culture) mix, filter-aided sample preparation (FASP)-based protein digestion and additional immobilized metal affinity chromatography (IMAC) enrichment of phosphopeptides before data-dependent acquisition (DDA) on the mass spectrometer. *Median age of each patient group or subgroup.

**Figure 2:**
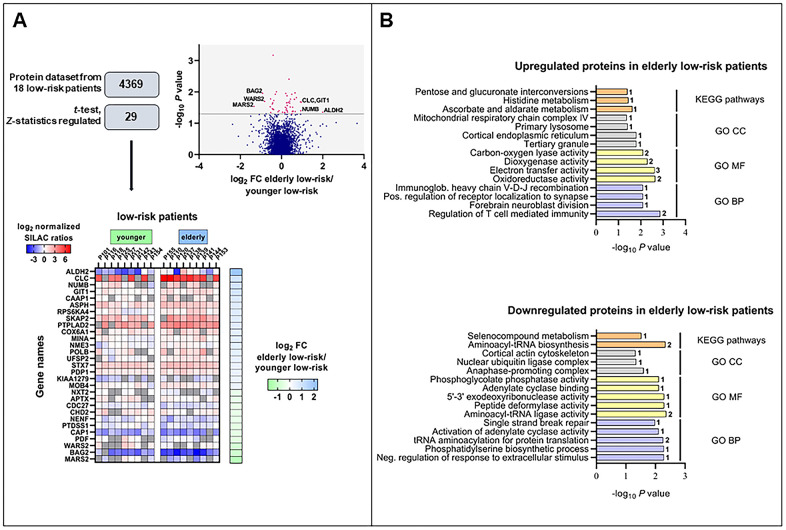
**The regulated proteome in the study of elderly low-risk *vs* younger low-risk patients.** (**A**) Overview of proteomic data analysis from elderly low-risk and younger low-risk patient samples. Volcano plot analysis of proteins quantified in at least five patients per group. Points (in magenta) above the non-axial horizontal grey line represent proteins with significantly different abundances (*P* <0.05). Heatmap of the 29 differentially expressed proteins in the elderly low-risk and younger low-risk groups. The log_2_ of fold change (FC) of protein levels in the elderly low-risk relative to the younger low-risk group is displayed on the right of the heatmap. (**B**) Gene Ontology (GO; BP, biological processes, with lilac bars; CC, cellular compartments, with light grey bars; MF, molecular functions, with yellow bars) and KEGG pathways (orange bars) analyses of upregulated and downregulated proteins in the elderly low-risk group. The various enriched GO terms and KEGG pathways are displayed on the y-axis while the corresponding –log_10_ *P* values are shown on the x-axis. The number of genes associated to a specific GO term or KEGG pathway is shown on the right side of the corresponding bar. Abbreviations were used in cases of long GO term (Immunoglob. for Immunoglobulin; Pos. for Positive and Neg. for Negative).

We obtained the proteome profiles of AML cells derived from nine elderly low-risk and nine younger low-risk patients using our FASP-based workflow (Figure 1). We quantified 5966 proteins, of which 4369 had a quantitative value in at least five patients in each group (Figure 2A). The proteome profiles from the elderly low-risk and younger low-risk patients were compared using *t*-test based statistical analysis and resulted in a small set of only 29 differentially expressed proteins, with 18 of them being upregulated and 11 downregulated for the elderly low-risk patients (Figure 2A, Supplementary File 1, Supplementary Table 3 upper part, Supplementary Table 4). Gene ontology (GO) enrichment analyses showed that regulation of T cell mediated immunity (i.e. syntaxin-7, STX7, and galectin-10, CLC) and oxidoreductase activity (i.e. aspartyl/asparaginyl beta-hydroxylase, ASPH, and ribosomal oxygenase 2, RIOX2) were more abundant GO terms in elderly low-risk patients (Figure 2B, top plot). The proteins enriched in this group were primarily located in organelles such as mitochondria and endoplasmic reticulum. Kyoto Encyclopedia for Genes and Genomes (KEGG) pathways analysis demonstrated that the upregulated elderly low-risk proteome was enriched with histidine, ascorbate and aldarate metabolism pathways (i.e. mitochondrial aldehyde dehydrogenase, ALDH2). In contrast, proteins involved in tRNA aminoacylation for protein translation (e.g. mitochondrial tryptophan-tRNA ligase, WARS2, and mitochondrial methionine-tRNA ligase, MARS2) and 5’-3’ exodeoxyribonuclease activity (e.g. aprataxin, APTX) were less plenteous in elderly low-risk patients (Figure 2B, bottom plot).

It can be seen from Supplementary Table 3 that a major part of the proteins with differential expression is involved in either transcriptional regulation (five proteins), protein homeostasis/modulation (10 proteins) or mitochondrial functions/metabolism (10 proteins). Altered epigenetic/transcriptional regulation is regarded as a characteristic feature of aging in hematopoietic stem cells [7, 22, 23] together with alterations in metabolism [24–26] and/or mitochondrial functions [27–30], protein homeostasis [13, 22] and DNA repair/genomic instability [7, 23, 31]. Thus, even though the comparative analysis of the proteome from elderly and younger low-risk AML patients showed only 29 differentially expressed proteins, most of these proteins are involved in the regulation of cellular processes that are known to be altered in aging cells. Among them, NUMB (protein numb homolog) is of particular interest because it is involved in the regulation of asymmetrical cell division [32] and altered cell polarity is a hallmark of aging [31].

### The effect of aging on the AML cell phosphoproteome; a comparison of elderly and younger low-risk patients

**Figure 3:**
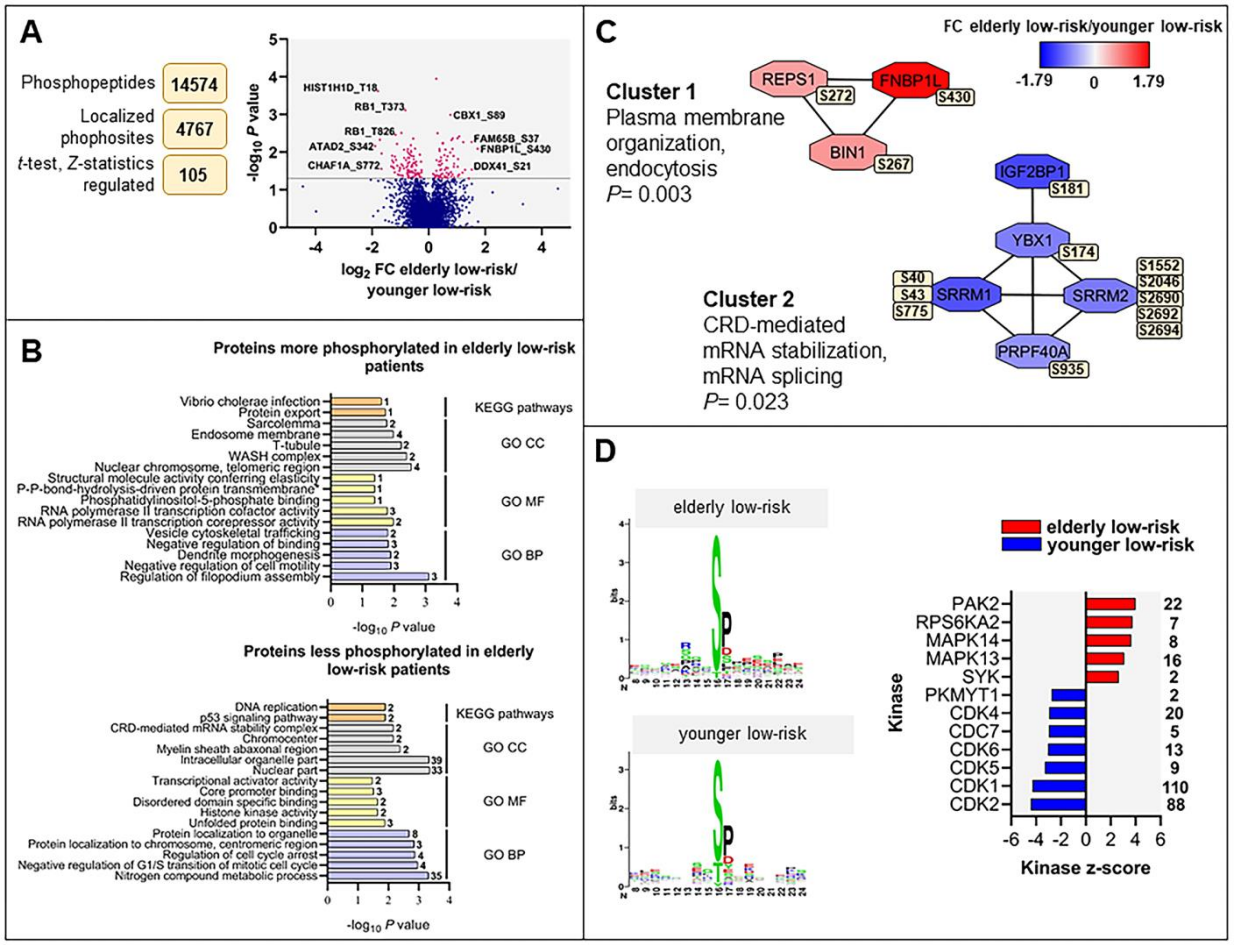
**The regulated phosphoproteome in the study of elderly low-risk *vs* younger low-risk patients.** (**A**) Overview of phosphoproteomic data analysis from elderly low-risk and younger low-risk patient samples. Volcano plot analysis of phosphosites quantified in at least five patients per group. Points (in magenta) above the non-axial horizontal grey line represent phosphosites with significantly different phosphorylation levels (*P* <0.05). (**B**) GO (BP terms with lilac bars; CC terms with light grey bars; MF terms with yellow bars) and KEGG pathways (orange bars) analyses of proteins with increased and decreased phosphorylation in elderly low-risk patients. The various enriched GO terms and KEGG pathways are displayed on the y-axis while the corresponding –log_10_ *P* values are shown on the x-axis. The number of genes associated to a specific GO term or KEGG pathway is shown on the right side of the corresponding bar. (**C**) Networks of protein-protein interactions (PPI) based on STRING database and visualized in Cytoscape after ClusterONE analysis. The significance of networks with high cohesiveness is shown with the *P* value of a one-sided Mann-Whitney U test. The differentially regulated phosphorylation sites are shown in yellow boxes next to each protein. FC of phosphorylation are color-coded; red-colored proteins showed a higher phosphorylation in elderly low-risk patients and blue-colored proteins showed a higher phosphorylation in the younger low-risk group. (**D**) Sequence motif analysis of the ± eight amino acids flanking the differentially regulated phosphorylation sites for each patient group and kinase-substrate enrichment analysis (KSEA) of differentially regulated and unregulated phosphorylation sites. The kinase z-score (x-axis) is the normalized score for each kinase (y-axis), weighted by the number of identified substrates indicated on the right side of the plot. Only significant predicted kinases with false discovery rate (FDR) values <0.05 were shown.

We constructed a dataset comprising 14,574 identified phosphopeptides, from which 11,962 class I protein phosphorylation sites were quantified on 2818 proteins of nine elderly low-risk and nine younger low-risk patients. We identified 105 differentially regulated phosphorylation sites based on statistical analysis of 4767 phosphosites that could be quantified in at least five patients in each of these two groups (Figure 3A and Supplementary File 2). A cluster including 43 significantly upregulated and another cluster including 62 downregulated phosphosites in the elderly relative to the younger low-risk group were subjected to GO and KEGG pathways enrichment analysis (Supplementary Tables 3, 5, 6).

Regulation of filopodium assembly, vesicle cytoskeletal trafficking and RNA polymerase II transcription cofactor activity (Figure 3B, upper plot) represented GO terms with higher protein phosphorylation in elderly low-risk patients. These terms include phosphoproteins such as formin-binding protein 1-like (FNBP1L), protein kinase C-binding protein 1 (ZMYND8), Rho family-interacting cell polarization regulator 2 (FAM65B), dysbindin (DTNBP1), TP53-binding protein 1 (TP53BP1) and arginine-glutamic acid dipeptide repeats protein (RERE). The phosphoproteins enriched in elderly low-risk patients were primarily located in nuclear chromosomes, WASH complex, transverse tubules and endosome membranes. ARID1A (AT-rich interactive domain-containing protein 1A) may be of particular importance because this protein is a regulator of CDC42 (cell division control protein 42 homolog) which plays an essential role in the regulation of actin and tubulin organization and thereby regulation of cellular polarity that can be lost in aging hematopoietic cells [13, 22]. KEGG pathways analysis showed similarities with the results from the GO term enrichment, i.e. the upregulated elderly low-risk phosphoproteome was enriched for protein export pathways. Several phosphoproteins involved in nitrogen compound metabolic process (e.g. histone-lysine N-methyltransferase 2A, KMT2A, and eukaryotic translation initiation factor 3 subunit F, EIF3F), regulation of cell cycle arrest (e.g. cyclin-dependent kinase 1 and 2, CDK1 and CDK2; nucleophosmin, NPM1) and transcriptional activator activity (e.g. ribosomal RNA processing protein 1 homolog B, RRP1B) were less phosphorylated in elderly low-risk patients (Figure 3B, bottom plot). Proteins less phosphorylated in this group showed a significant enrichment of DNA replication and p53 signaling KEGG pathways.

Several protein-protein interactions (PPI) networks of significant cohesiveness were found after ClusterONE analysis based on STRING interactions of differentially phosphorylated proteins (Figure 3C). The most significant network (cluster 1) consisted of three phosphoproteins with higher phosphorylation in elderly low-risk patients and being involved in plasma membrane organization and endocytosis (i.e. protein export found in the KEGG analysis). The second significant cluster involved mRNA stabilization and splicing proteins. Serine/arginine repetitive matrix protein 1 and 2 (SRRM1 and SRRM2) showed higher phosphorylation on multiple sites in younger low-risk relative to elderly low-risk patients. These splicing factors are phosphorylated on multiple serine and threonine residues by dual specificity tyrosine-phosphorylation-regulated kinase 3 (DYRK3) during the G2-to-M transition, after the nuclear-envelope breakdown [33] (i.e. regulation of transcription and cell cycle, see the KEGG analyses).

As shown in Supplementary Table 3, a majority of the total set of the differentially regulated phosphorylated sites were located in proteins involved in RNA synthesis/function (i.e. transcription factors, epigenetic regulation, histone modulation, RNA splicing and ribosomal regulation). Such altered transcriptional regulation is regarded as a hallmark of aging [31]; it is also a characteristic of hematopoietic stem cell aging [19, 20] and thus an important characteristic of AML in elderly patients [7].

### Differences in AML cell kinase activity when comparing elderly low-risk and younger low-risk patients

To identify protein kinases differentially activated in elderly low-risk and younger low-risk patients we performed phosphorylation site motif analysis with the WebLogo tool [34] (Figure 3D, left plots). We found higher activity of several kinases such as protein kinase A and C (PRKACA, PRKCD; basophilic motif upstream to the differentially phosphorylated site), calmodulin-dependent protein kinase II (CaM kinase II; basophilic motif upstream to the differentially phosphorylated site), serine/threonine protein kinase PAK2 (basophilic motif upstream to the differentially phosphorylated site), casein kinase 2 (CSK2; acidic amino acid-based motif downstream to the differentially phosphorylated site) and extracellular signal-regulated kinases (ERK1/2; proline-directed motif downstream to the differentially phosphorylated site) in elderly low-risk patients. In contrast, cyclin-dependent kinases (CDKs; proline-directed motif downstream to the differentially phosphorylated site) substrates appeared more phosphorylated in the younger low-risk group.

The kinase-substrate enrichment analysis (KSEA) [35, 36], which is based on phosphorylation fold changes (FCs) to estimate kinase’s activity, confirmed the higher activity of PAK2 and mitogen-activated protein kinases (MAPKs) in elderly low-risk patients and the higher activity of CDKs in the younger low-risk group (Figure 3D, right plot). KSEA analysis revealed a high number of CDK1 and CDK2 substrates from proteins such as ARID1A, LIG1 (DNA ligase 1), FOXK2 (forkhead box protein K2), NPM1 and retinoblastoma-associated protein (RB1).

We found five phosphosites in three different protein kinases in this data set using the activation loop analysis tool (see Materials and Methods). All of them (CDK1 T14, CDK2 T14, CDK1 Y15, CDK2 Y15 and GSG2 S147) can be phosphorylated during mitosis and were upregulated in younger low-risk relative to elderly low-risk patients.

### Proteomic comparison of high-risk and all low-risk patients; different expression of neutrophil degranulation, platelet degranulation and cytoskeleton proteins

**Figure 4:**
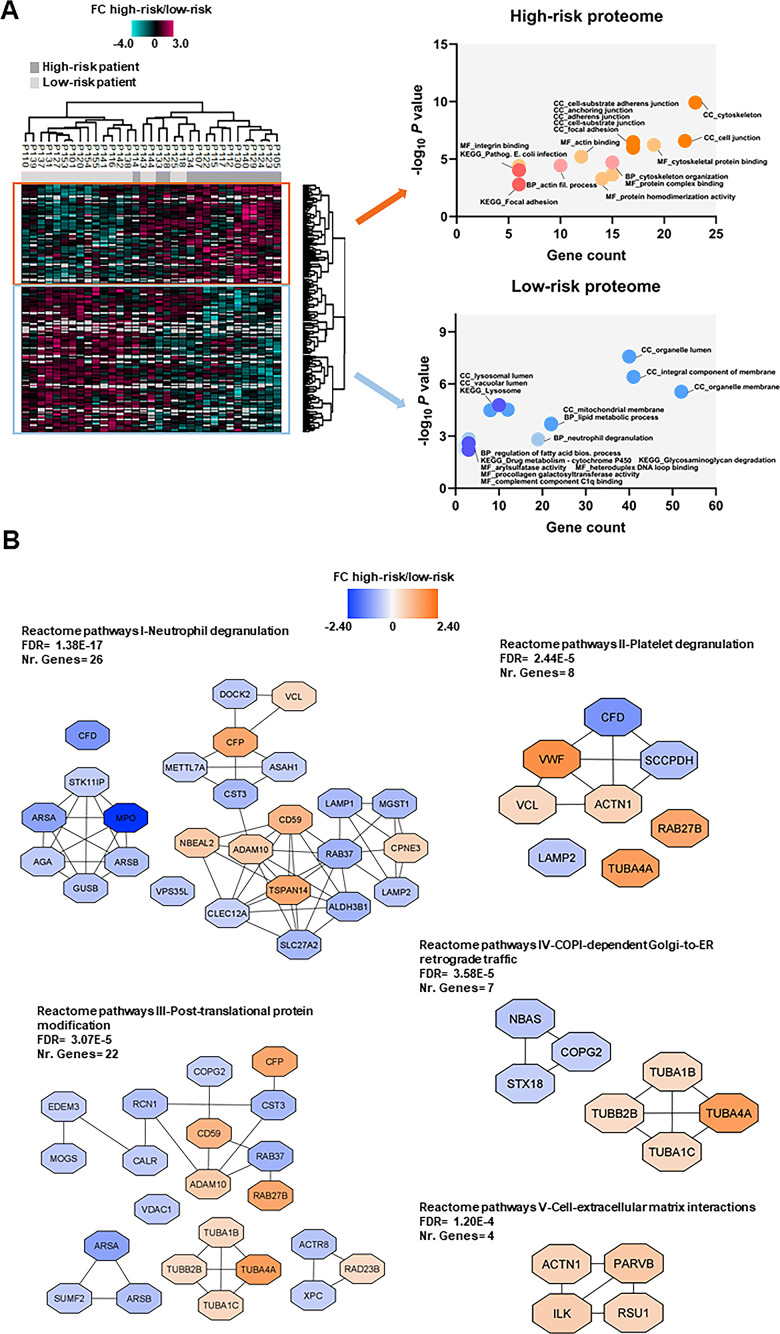
**Proteomic differences between high-risk and low-risk patients; the importance of the cytoskeleton reflected in levels of neutrophil degranulation, platelet degranulation and endomembrane trafficking proteins.** (**A**) Hierarchical clustering of 33 patients was based on the expression (SILAC log_2_ ratio) of 205 proteins with significantly different regulation in AML cells from high-risk (dark grey squares) and low-risk patients (light grey squares). Two vertical main clusters were observed, one dominated by proteins with higher abundance in mostly high-risk patients (upper cluster) and the other by proteins with higher abundance in low-risk patients (lower cluster). GO and KEGG pathways analyses of the two protein clusters were performed to reveal enriched BP, CC and MF terms for the high-risk and low-risk patients. The various enriched GO terms and KEGG pathways are displayed in the scatter plot. The number of genes associated to a specific GO term or KEGG pathway (count) and the corresponding –log_10_ *P* values are shown on the x-axis and y-axis, respectively. Abbreviations were used in cases of long GO term or KEGG pathway name (Pathogen. for Pathogenic; fil. for filament). (**B**) Reactome term enrichment was performed using the STRING app (1.5.1) in Cytoscape. The five Reactome pathways with highest significance are shown with the corresponding FDR values. The protein nodes are colored according to their high-risk/low-risk FC, i.e. orange indicates increased abundance in the high-risk group and blue increased abundance in the low-risk group.

We compared the proteome profiles of AML cells derived from 15 high-risk and 18 low-risk patients. We quantified 6569 proteins, of which 5009 had a quantitative value in at least five patients in each group. The *t*-test based statistical analysis resulted in 205 differentially expressed proteins, 82 proteins were upregulated and 123 were downregulated in high-risk relative to low-risk patients (Supplementary Figure 1A and Supplementary File 3). Hierarchical clustering based on the 205 regulated proteins identified two main patient clusters (Figure 4A, left part), which corresponded to the high-risk and low-risk samples, although a distinct separation was not obtained. GO enrichment analysis showed that cytoskeleton organization, actin binding and integrin binding as biological process and molecular function GO terms were over-represented in high-risk patients (Figure 4A, right part). These terms include several tubulin (TUBB) chains, protein-tyrosine kinase 2-beta (PTK2B), cytoplasmic linker-associated protein 1 (CLASP1), actin-related protein 2/3 complex subunit 1A (ARPC1A), NCK-interacting protein with SH3 domain (NCKIPSD), TNF receptor-associated factor 2 (TRAF2) and NCK-interacting protein kinase (TNIK), alpha-actinin-1 (ACTN1) and integrin-linked protein kinase (ILK). Moreover, KEGG pathways analysis confirmed that the focal adhesion and pathogenic *E. coli* infection pathways (i.e. several tubulin chains and actin-related protein 2/3 complex subunits) were enriched in the upregulated high-risk proteome.

In the low-risk proteome, proteins involved in neutrophil degranulation (i.e. extracellular secretion) and lipid metabolic processes such as complement factor D (CFD), myeloperoxidase (MPO), lysosome-associated membrane glycoprotein 1 and 2 (LAMP1/2), mitochondrial enoyl-CoA delta isomerase 1 (ECI1), 3-ketoacyl-CoA thilase, mitochondrial (ACAA2) and lysophosphatidylcholine acyltransferase 2 (LPCAT2) were higher expressed (Figure 4A, right part). Arylsulfatase and procollagen galactosyltransferase activities were the molecular functions more abundant in the low-risk group. As much as 43% of the proteins with higher abundance in low-risk patients were annotated to the organelle membrane in the cellular component analysis.

Analysis of Reactome terms showed that neutrophil degranulation (Reactome pathways I), platelet degranulation (Reactome pathways II) -both these granulation processes reflecting the extracellular secretion in myeloid cells-, post-translational protein modification (Reactome pathways III), COPI-dependent Golgi-to-ER retrograde traffic (Reactome pathways IV) and cell-extracellular matrix interactions (Reactome pathways V) pathways were enriched in the set of 205 regulated proteins (Figure 4B and Supplementary Table 7). We observed several clusters of upregulated proteins in the high-risk patient group in Reactome pathways III, IV and V including cytoskeleton proteins such as tubulin chains, ACTN1, ILK, beta-parvin (PARVB) and Ras suppressor protein 1 (RSU1). Clusters of upregulated proteins in the low-risk patient group were found in other Reactome pathways, such as the one comprised of arylsulfatase A (ARSA), arylsulfatase B (ARSB), MPO, N(4)-(beta-N-acetylglucosaminyl)-L-asparaginase (AGA), beta-glucuronidase (GUSB) and serine/threonine-protein kinase 11-interacting protein (STK11IP) in Reactome pathways I; ARSA, ARSB and inactive C-alpha-formylglycine-generating enzyme 2 (SUMF2) in Reactome pathways III; and syntaxin-18 (STX18), neuroblastoma-amplified sequence (NBAS) and coatomer subunit gamma-2 (COPG2) in Reactome pathways IV.

Few proteins in Reactome pathways I and II show an expression mainly or limited to myeloid cells, whereas a majority of them are expressed by various tissues/organs/cells and are important for membrane/organellar functions or protein metabolism/modulation. Thus, these networks seem to mainly reflect differences in fundamental and common cellular processes more than differences in the differentiation status of the cells.

### Only ALDH2 had a similar differential expression both when comparing elderly low-risk *vs* younger low-risk and high-risk *vs* all low-risk patients

A main difference between our first comparison of elderly low-risk *vs* younger low-risk patients and this second comparison of high-risk *vs* all low-risk patients is the higher number of differentially expressed proteins in the latter analysis. Furthermore, only three proteins, ALDH2, Ufm1-specific protease 2 (UFSP2) and BAG family molecular chaperone regulator 2 (BAG2) were differentially regulated in both studies (Supplementary Figure 2A). Only ALDH2, a poor prognosis predictor in AML as well as in urothelial cancer ([37]; http://www.proteinatlas.org) showed a similar upregulation in both comparisons. Western blot analyses using lysates from nine high-risk and nine low-risk (five elderly low-risk and 4 younger low-risk) patient cells showed higher ALDH2 expression in high-risk and elderly low-risk when compared to all low-risk and younger low-risk patients, respectively (Supplementary Figure 3), although differences between groups were not statistically significant according to the Mann-Whitney test.

These observations are consistent with the hypothesis that ALDH2 expression reflects an adverse prognostic impact of age in AML.

### Phosphoproteomic comparison of high-risk *vs* all low-risk patients; detection of differences in mitotic cell cycle regulation

**Figure 5:**
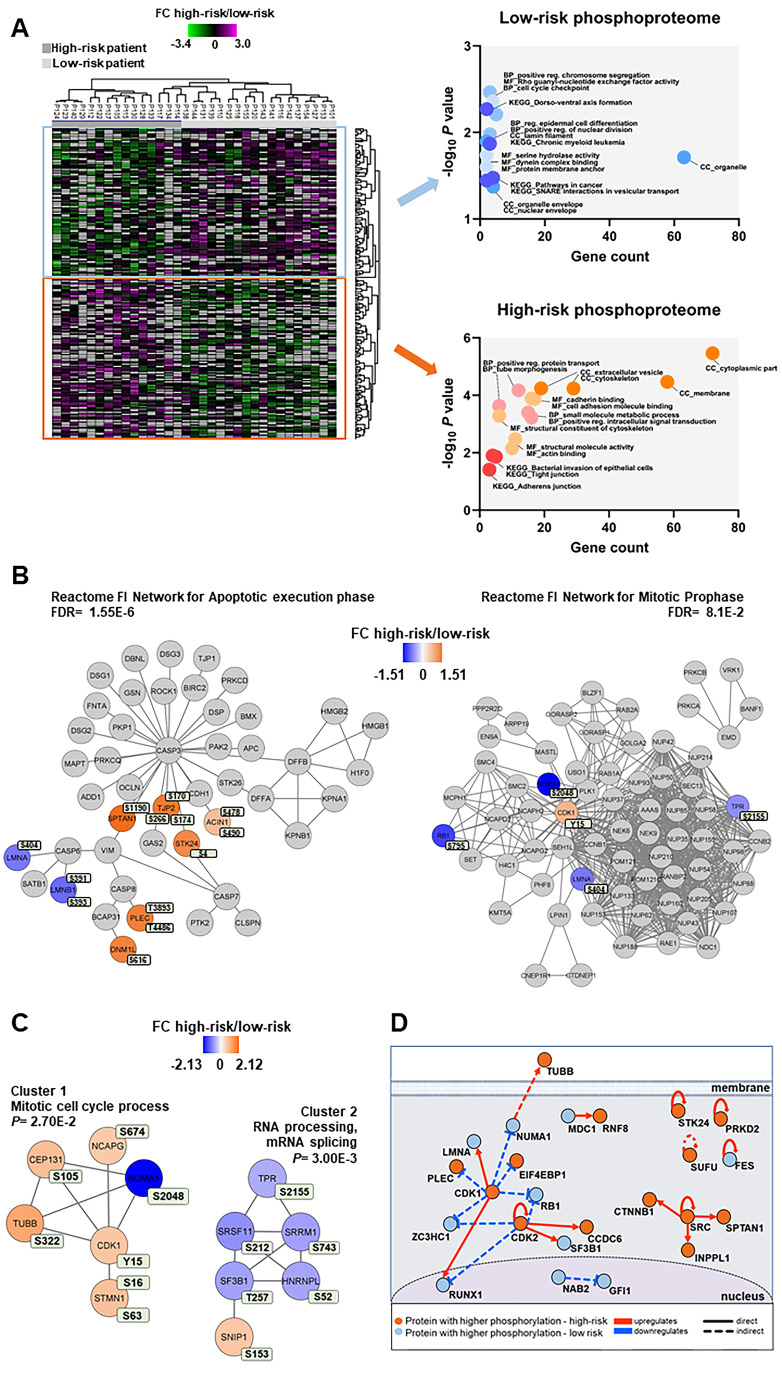
**Phosphoproteomic differences between high-risk and low-risk patients; the importance of the cytoskeleton, mitotic cell cycle regulation and CDK activities.** (**A**) Hierarchical clustering of the 33 patients based on the phosphorylation level (SILAC log_2_ ratio) of 239 phosphosites with significant differences between high-risk and low-risk patient samples. Two vertical main clusters were observed, one dominated by phosphosites with higher phosphorylation in low-risk patients (upper cluster) and the other by phosphosites with higher phosphorylation in high-risk patients (lower cluster). GO and KEGG pathways analyses of the two corresponding phosphoprotein clusters were performed to reveal enriched BP, CC and MF terms in the high-risk and low-risk patient samples. The various enriched GO terms and KEGG pathways are displayed in the scatter plot. The number of genes associated to a specific GO term or KEGG pathways (count) and the corresponding –log_10_ *P* values are shown on the x-axis and y-axis, respectively. Abbreviations were used in cases of long GO term or KEGG pathway name (reg. for regulation). (**B**) Visualization of hit Reactome pathways was performed using the ReactomeFIViz app (7.2.3) in Cytoscape. Two significant Reactome networks (FDR <0.05) that mapped phosphoproteins with differential phosphorylation in our dataset are shown. The protein nodes are colored according to their high-risk *vs* low-risk log_2_ phosphorylation FC, i.e. orange indicates increased phosphorylation in the high-risk group and blue increased phosphorylation in the low-risk group. (**C**) Networks of PPI based on STRING database and visualized in Cytoscape after ClusterONE analysis. The significance of networks with high cohesiveness is shown with the *P* value of a one-sided Mann-Whitney U test. The differentially regulated phosphorylation sites are shown in light green boxes next to each protein. FC of phosphorylation are color-coded; orange-colored proteins showed a higher phosphorylation in the high-risk group and blue-colored proteins showed a higher phosphorylation in the low-risk group. (**D**) Causal relationships between phosphoproteins with differentially regulated phosphorylation sites in the high-risk *vs* low-risk phosphoproteome set was studied with SIGNOR. The analysis showed the pivotal role of CDKs in the control of cell cycle, cytoskeleton and translation phosphoproteins. Nodes and types of relationships are displayed as indicated on the bottom part of the design.

We identified and quantified 14,990 class I protein phosphorylation sites from 3279 proteins when comparing 15 high-risk and 18 low-risk patients. We found 239 differentially regulated phosphorylated sites based on statistical analysis of 6682 phosphosites, which were quantified in at least five patients in each group (Supplementary Figure 1B and Supplementary File 4). Hierarchical clustering using these 239 phosphosites clearly distinguished the phosphoproteome of the two patient groups (Figure 5A, left part). Two clusters, one containing 124 phosphosites and another with 115 phosphosites, were upregulated in the high-risk and in the low-risk patient group, respectively.

Cellular component GO analysis revealed an enrichment of upregulated cytoplasmic, cytoskeleton and membrane phosphoproteins for the high-risk patients, whereas organelle, organelle envelope and nuclear envelope structures were enriched in low-risk patients (Figure 5A, right part). While cell adhesion molecule binding, positive regulation intracellular signal transduction and small molecule metabolic process were the biological process and molecular function GO terms enriched in the high-risk group, positive regulation chromosome segregation, cell cycle checkpoint and Rho guanyl-nucleotide exchange factor activity were enriched in the low-risk patient group (Figure 5A, right part).

Reactome pathways for apoptotic execution phase and for mitotic prophase were found significantly enriched with proteins mainly of higher phosphorylation in the high-risk and in the low-risk patient group, respectively (Figure 5B and Supplementary Table 7). Two PPI networks of significant cohesiveness were found after ClusterONE analysis based on STRING interactions of differentially phosphorylated proteins (Figure 5C and Supplementary Table 7). The most significant network (cluster 2) consisted of six phosphoproteins involved in RNA processing and mRNA splicing, most of them with higher phosphorylation in low-risk patients. The other significant cluster included phosphoproteins of the mitotic cell cycle process (cluster 1). All phosphoproteins, except nuclear mitotic apparatus protein 1 (NUMA1), were significantly more phosphorylated in high-risk patients. A sequence logo analysis of the amino acids surrounding the phosphosites in cluster 1 suggested PRKACA and kinases of the PRKC family involved in the phosphorylation of the six cell cycle proteins (Supplementary Figure 4).

A final analysis of the regulated high-risk *vs* low-risk phosphosite set to study the interactions of their corresponding phosphoproteins in signal transduction with SIGNOR [38] (Figure 5D) confirmed the relevant roles of CDKs in the regulation of the cell cycle, RNA processing, translation and cytoskeleton function that are observed in AML patients with different risk-related cytogenetics abnormalities.

### Several protein kinases are differentially activated in AML cells derived from high-risk and all low-risk patients

**Figure 6:**
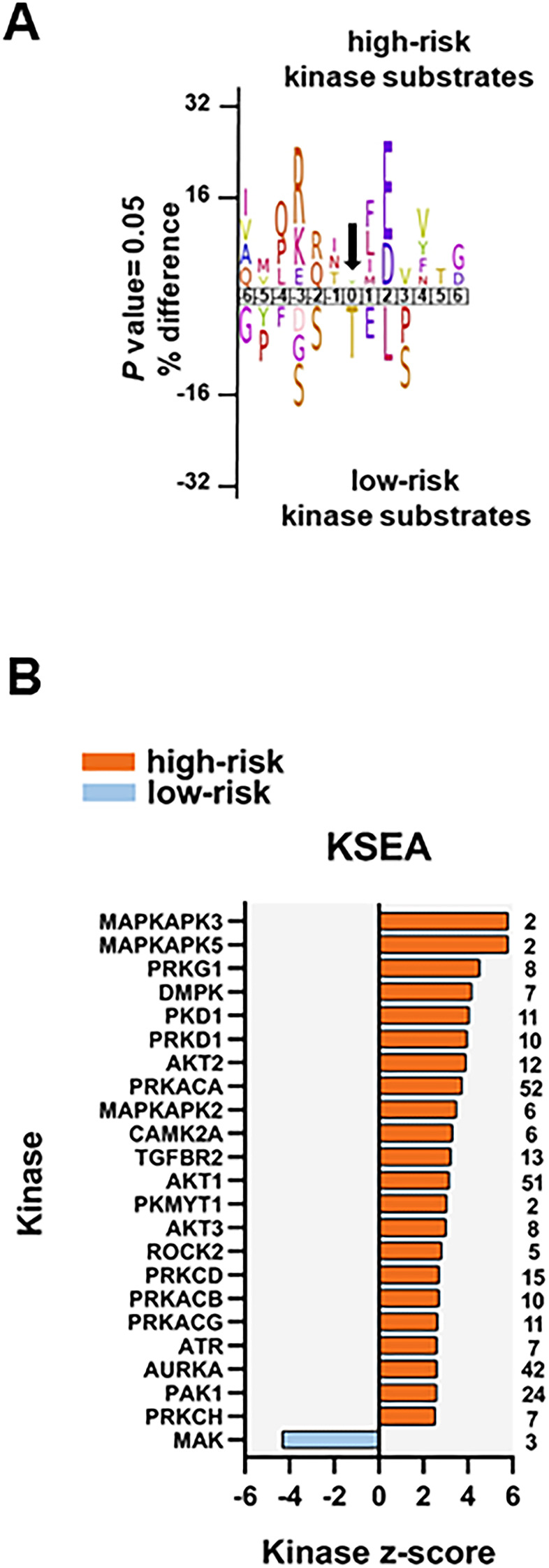
**Kinase prediction analysis of the high-risk *vs* low-risk phosphoproteome.** (**A**) Sequence motif analysis of the ± six amino acids flanking the differentially regulated phosphorylation sites for either group. (**B**) KSEA of differentially regulated and unregulated phosphorylation sites. The kinase z-score (x-axis) is the normalized score for each kinase (y-axis), weighted by the number of identified substrates indicated on the right side of the plot. Significant predicted kinases with FDR <0.05 are shown.

To identify protein kinases differentially activated in the two groups we performed phosphorylation site motif analysis with IceLogo [39]. We found a basophilic motif upstream to the differentially phosphorylated site in high-risk patients when compared to low-risk patients, suggesting an activation of PRKACA, PRKCA and PRKCD (Figure 6A). Furthermore, KSEA confirmed the higher activity of PRKACA and predicted several other serin/threonine protein kinases (PRKG1, PRKD1 and PRKCD), MAPKs and RAC-alpha serine/threonine protein kinase (AKT) isoforms activated in the high-risk patient group (Figure 6B). Although PRKACA and AKT1 phosphorylated a large number of substrates (52 and 51, respectively), CDK1 (significantly predicted with unadjusted *P*= 0.022 in the KSEA; therefore not displayed in Figure 6B which shows predicted kinases at FDR <0.05) phosphorylated 140 substrates in this group. Serine/threonine protein kinase MAK, which phosphorylates proteins involved in protein ubiquitation and in the transcriptional coactivation of androgen receptor, was activated in the low-risk patient group.

We found eight phosphosites on five different protein kinases in the data set of 239 differentially regulated phosphorylation sites. Three phosphosites on serine/threonine-protein kinase D2 (PRKD2; S197, S198 and S706; the latter located in the activation loop of the kinase), involved in the regulation of cell proliferation via ERK1/2 signaling, in Golgi membrane trafficking and cell adhesion, were significantly more phosphorylated in the high-risk patient group. PRKD2 S706 is probably phosphorylated by the members of the PRKC family [40]. SRC S17 and CDK1 Y15 were also significantly more phosphorylated in the high-risk patient group. Phosphorylation of CDK1 Y15 by Wee-1 like protein kinase 1/2 (WEE1/2) inhibits the protein kinase activity and acts as a negative regulator of entry into mitosis (G2 to M transition) whilst phosphorylation by PRKCD activates the G2-M DNA damage checkpoint after UV irradiation [41]. Dephosphorylation at CDK1 Y15 by active M-phase inducer phosphatase 1/2 (CDC25A/B) leads to CDK1 activation at the G2-M transition [42]. The higher phosphorylation of CDK1 (and CDK2) Y15, along with T14 phosphorylation, was further confirmed by a separate MS-based immune-affinity enrichment (Supplementary Table 8 and Supplementary File 5). The significantly higher phosphorylation of CDK1/2 T14 alone in high-risk patients was also observed in the latter analysis (not significant in the general phosphopeptide enrichment analysis with *P*= 0.078). Finally, tyrosine-protein kinase Fes/Fps (FES), a regulator of the actin cytoskeleton and microtubule assembly, S408; and STK26 (serine/threonine-protein kinase 26), a mediator of cell growth, T327 and T328 were significantly more phosphorylated in the low-risk patient group.

The results from Western blotting did not show any significant difference in protein phosphorylation of several kinase candidates between the high-risk and the low-risk groups (Supplementary Figure 5). However, despite the different spectrum of sensitivity of both methods, the increased activity of CDK1, PRKCD and PRKACA in the high-risk group detected by the MS-based data was also observed in the Western blots (Supplementary Figure 5). We also noticed a distinct CDK1 T161-Y15 phosphorylation pattern for each of the patient groups. While these two phosphosites were similarly phosphorylated in low-risk patients, CDK1 T161 phosphorylation was different of that observed in CDK1 Y15 in high-risk patients suggesting a different CDK1 regulation for each of the groups. Moreover, the phosphorylation levels of PRKCD S645 and PRKACA T197 were parallel for each patient showing a joint activation of both kinases.

### Only LSP1 had a similar differential phosphorylation both when comparing elderly low-risk *vs* younger low-risk and high-risk *vs* all low-risk patients

Five phosphosites on proteins lymphocyte-specific protein 1 (LSP1), lamina-associated polypeptide 2, isoform alpha (TMPO), CDK1, CDK2 and N-acetyltransferase ESCO2 (ESCO2) were quantified in both our comparisons, i.e. elderly low-risk *vs* younger low-risk and high-risk *vs* all low-risk patients (Supplementary Figure 2B). Only LSP1_S177, an actin-binding cytoskeleton protein involved in cell migration and possibly phosphorylated at that residue by MAPKAPK2 and/or protein kinase C [43], showed significant associations with high age/increased risk of relapse in both our comparisons. However, CDK1/2 Y15 showed increased phosphorylation in high-risk and in younger low-risk patients. These apparently conflicting observations on CDK’s activity illustrate that the impact of certain age-associated proteomic differences may differ between patient subsets and depend on the biological/genetic context.

## DISCUSSION

Elderly AML patients seem to have more chemoresistant disease than younger patients; this is at least partly due to a lower frequency of low-risk genetic abnormalities and a higher frequency of at least certain high-risk abnormalities in elderly patients [1, 6] although some high-risk abnormalities (e.g. MLL abnormalities) do not show such age-dependent differences [44].

Many elderly patients cannot receive intensive and potentially curative treatment due to an unacceptable risk of treatment-related mortality [1], and elderly patients receiving intensive treatment do not receive the same consolidation therapy as the younger patients, e.g. patients above 60 years of age cannot receive high-dose cytarabine [1]. A difference in the survival for younger and elderly AML patients may therefore reflect differences in chemotherapy and not (only) differences in chemosensitivity. However, younger and elderly AML patients often receive the same induction chemotherapy, and differences in the complete remission rate will therefore reflect differences in chemosensitivity that also are relevant for long-term survival [45]. Several observations suggest that elderly patients have a lower remission rate. First, the initial experience after introduction of the conventional “7+3” cytarabine/anthracycline induction regimen showed a long-term remission of 20-25% for younger and 10% for elderly patients [46]. Second, a large study of intensive treatment showed a significant decrease of complete remission rate with age; the rate was 65% for patients below 56 years of age but only 33% for patients above 75 years [47]. Finally, several studies have investigated the remission rate of patients above 60 years of age receiving the conventional “7+3” regimen but with the increased daunorubicine dose of 60 mg/m^2^/day. Complete remission rates corresponding to 33-51% of the patients have been observed for elderly patients [48, 49] whereas younger patients have shown remission rates exceeding 70% [50, 51] and this higher rate is expected because previous studies of younger patients have described remission rates corresponding to 60-85% [52]. Taken together these observations suggest that elderly AML patients have a more chemoresistant disease.

Normal hematopoietic stem cells develop biological signs of aging [12–20] and the aim of our study was therefore to investigate whether similar biological characteristics of aging could be detected at the proteomic and phosphoproteomic level in AML cells derived from elderly patients. To do so we only included patients with relatively high levels of circulating AML cells (i.e. peripheral blood leukemization) so that enriched cell populations could then be prepared by simple gradient separation alone [53, 54].

In the present study we wanted to compare patient subsets with regard to *in vivo* chemosensitivity (i.e. AML-free survival) and the association between leukemization and *in vivo* chemosensitivity is regarded as uncertain or weak compared with the impact of genetic abnormalities [45]. Several studies have suggested that a prognostic impact of leukemization is seen only when peripheral blood blast counts exceed 100 x 10^9^/L [55–57]. Only a small minority of our patients had such high blood blast counts; therefore, the impact of those few patients on our present results is in our opinion limited.

Bone marrow samples were not used because the diagnostic criteria for AML is usually at least 20% blasts, but for patients with favorable cytogenetic abnormalities there is no such blast criteria [58]. More extensive cell separation procedures that may alter the biological characteristics of the leukemic cells [53] would have therefore been necessary if bone marrow samples had been used. Moreover, blood and bone marrow AML cells seem to have only minor differences [59], and even clonal heterogeneity as well as hierarchical organization of the AML cell clones can be demonstrated in peripheral blood AML cells [60, 61]. The use of cryopreserved biobank samples allowed us to carefully select patients according to our predefined cytogenetic criteria, and follow-up experiments of the same patients were also possible.

Our study should be regarded as population-based because we included all patients in a defined geographical area who were diagnosed with AML during a defined time period. For this reason, our study included a relatively large number of elderly patients, and many elderly and unfit patients did not receive intensive and potentially curative antileukemic treatment [1]. Twelve out of 18 low-risk patients received intensive treatment, and this is expected from the age distribution of these patients. Only four of these 12 patients became long-term survivors. This is not unexpected as one patient was lost from follow-up, we had one early death due to hyperleukocytosis, three elderly patients died from toxicity during intensive consolidation therapy, one patient did not receive consolidation therapy due to severe toxicity and one allotransplanted patient died from Graft versus host disease. Taken together this explains why we have a relatively low long-term AML free survival even for patients with low-risk disease who received intensive chemotherapy.

A relatively high non-relapse mortality is expected for elderly patients [1], and this was also seen in our present study. Our patients were selected according to cytogenetic criteria, and when taking into account that the median age of patients with first diagnosis of AML is 65-70 years [1] it is expected that relatively few of our patients received intensive and potentially curative therapy because of their age. Due to this heterogeneity of our patients with regard to antileukemic treatment, extensive survival analyses were not possible.

Our high- and low-risk cell populations showed additional differences respecting patient age, frequency of secondary AML and morphological signs of differentiation. These differences are expected because favorable cytogenetic abnormalities are most common in younger patients [1, 6, 58]; AML secondary to previous chemotherapy or chronic myeloid malignancies (i.e. chronic myeloproliferative neoplasia, myelodysplastic syndrome) is most common for elderly patients [5, 62]; and both favorable cytogenetic abnormalities as well as *NPM1* mutations are associated with morphological signs of differentiation [58, 63, 64]. High age is associated with adverse prognosis, and to further investigate age-dependent factors independent of the genetic abnormalities we firstly investigated a group of patients with a limited number of well-defined genetic abnormalities (karyotype; receptor-type tyrosine-protein kinase *FLT3*, *FLT3*, *NPM1* and CCAAT/enhancer-binding protein alpha, *CEBPA,* mutations) generally accepted to be associated with a good prognosis [1]. Thus, we compared elderly and younger patients with a favorable prognosis based on analyses of these cytogenetic abnormalities (i.e. the elderly low-risk and the younger low-risk patient groups with median age of 68 and 47 years, respectively). This scientific strategy was chosen because we assumed that an additional age-dependent adverse prognostic impact may be easier to detect for patients with favorable prognosis than for patients with an already adverse prognosis due to their cytogenetic abnormalities. This approach is supported by a recent publication describing an independent adverse prognostic impact of secondary AML only for younger patients [5].

In the second analysis of this study we compared a group with adverse prognosis (i.e. the high-risk patient group with a higher median age of 74 years) *vs* a group with favorable prognosis (the whole low-risk patient group with a lower median age of 64.5 years) to investigate whether possible proteomic or phosphoproteomic characteristics identified in the first comparison could still be detected when comparing high-risk and low-risk patients. Our MS-based methodology is reliable and reproducible for this kind of studies as we have shown by validation with alternative non MS-based technologies in two previous papers [65, 66].

Among the hallmarks of aging, including hematopoietic stem cell aging, are altered cellular communication, altered intracellular trafficking/polarity influencing the communication with neighboring cells/stroma, detoxification/stress responses, and altered transcriptional regulation due to various different mechanisms including altered epigenetic regulation [23, 31]. The most important age-associated differences described in our present studies are increased ALDH2 levels (stress responses), cytoskeletal modulation (trafficking/mitosis/transport/polarity) and transcriptional regulation. These main differences can all be relevant for aging and may then be involved in leukemogenesis and chemosensitivity for elderly AML patients.

Aldehyde dehydrogenase (ALDH) proteins are intracellular enzymes that oxidize cellular aldehydes and thereby participate in regulation of differentiation and development of chemoresistance [67]. Both our primary AML cell comparisons suggested that the protein levels of ALDH2 are increased in elderly AML patients; this was true both when comparing elderly low-risk and younger low-risk patients, and high-risk *vs* all low-risk patients. Both experimental and clinical studies suggest that ALDH2 activity is important for leukemogenesis and/or chemosensitivity in AML. First, ALDH2 activity and *ALDH2* gene polymorphisms seem to be involved in carcinogenesis for various malignancies [68]. This seems to be true not only for solid tumors but also for leukemogenesis; the involvement of ALDH (including ALDH2) for progression of preleukemic Fanconi anemia to bone marrow failure/AML is suggested both by animal models and clinical studies [69–72]. Second, ALDH2 can influence the signaling through several intracellular pathways involved in regulation of apoptosis, and it can thereby have antiapoptotic effects [73]. ALDH inhibition has a synthetically lethal effect in AML cells when combined with glutathione peroxidase-4 inhibition [74], and it can overcome both bortezomib and cytarabine resistance in Down syndrome-associated AML [75]. High ALDH2 activity is also associated with resistance to doxorubicin [76], the drug that is combined with cytarabine in conventional AML induction chemotherapy [1]. Third, ALDH activity can be detected in primary human AML cells, but the activity differs between patients and also between cells within the same hierarchically organized AML cell populations [77]. Patients with a generally high ALDH activity in their AML cells show decreased survival [77]. There is an association between high ALDH activity and high-risk karyotype [78] and high ALDH activity in AML cells is associated with an increased risk of relapse for patients with the favorable t(8;21) abnormality [79]. A recent study even suggested that ALDH2 expression could be included in a 4-gene expression prognostic signature for patients with intermediate-risk AML [80]. Finally, ALDH activity is detected both in normal hematopoietic and leukemic stem cells, but the activity in these two cell types seems to differ [81]. This may explain the observations from previous studies describing that ALDH inhibition can eradicate leukemic stem cells but at the same time spares normal hematopoietic stem cells [73, 78]. Taken together these observations suggest that ALDH activity/ALDH2 expression is important for chemosensitivity in human AML, and our present study suggests that this impact is associated with aging.

Our comparison of patient cohorts also identified several cytoskeletal proteins that were associated with differences in age. The cytoskeleton is important for intracellular trafficking and exocytosis/endocytosis, mitosis, for the cellular contact with neighboring cells and the extracellular matrix [82, 83]. Firstly, cytoskeletal proteins differed both in their expression level and in their phosphorylation when comparing elderly low-risk and younger low-risk patients (Figure 3B, 3C; Supplementary Tables 3–6). Second, differences in cytoskeletal proteins were also detected when comparing high-risk *vs* all low-risk patients (Figures 4B, 5B and Supplementary Table 7), especially those ones involved in membrane trafficking, post-translational protein modification and extracellular matrix interactions. Cytoskeleton proteins can be altered as a part of the aging process [84, 85] and the associations between age and altered levels/phosphorylation of cytoskeletal proteins may therefore reflect an impact of aging in AML. Finally, it should be emphasized that ALDH2 is a cytoskeleton-interacting protein [86] and cytoskeletal proteins were also included in the various interacting molecular networks identified in our studies.

It can be seen from Supplementary Table 1 and Table 1 that morphological signs of monocytic differentiation is more common for younger patients with low-risk disease. This difference is expected because both favorable cytogenetic abnormalities as well as *NPM1* insertions are associated with myeloid differentiation [58, 63, 64]. Furthermore, the data presented in Figure 4 showed that high- and low-risk patients also differ with regard to proteins involved in neutrophil and platelet degranulation. The question is therefore whether the other observed proteomic or phosphoproteomic differences (e.g. organellar functions, regulation of proliferation/mitosis) observed in our study reflect differences in differentiation rather than chemosensitivity, but in our opinion associations with differentiation seem less likely. Important characteristics of the identified proteins belonging to the neutrophil and platelet degranulation networks (Figure 4B) are summarized in Supplementary Table 7. Most of these proteins are expressed in a wide range of cells/tissues and not only in myeloid cell subsets; an observation suggesting that these proteins are important for cellular functions with regard to organellar functions/intracellular trafficking and not only for myeloid cell subsets (i.e. not specific signs of myeloid differentiation). Furthermore, we investigated the *in vitro* proliferative responsiveness of primary AML cells derived from an external cohort of consecutive patients. Even though we observed an expected and statistically significant association between young age and monocytic differentiation for this cohort, neither *in vitro* proliferative responsiveness nor expression of stem cell/molecular differentiation markers showed any significant associations with morphological AML cell differentiation (Supplementary Analysis Tables 1, 2). Taken together these observations therefore suggest that the organellar/degranulation/mitosis networks identified in Figures 4B, 5B, 5C represented aging-dependent rather than differentiation-dependent differences between AML cells.

Both the proteomic and phosphoproteomic analyses suggested that mitotic regulation differs between elderly/high-risk and younger/low-risk patients. When analyzing the proliferative AML cell capacity for a group of consecutive patients we did not find any evidence for a general association between proliferative capacity and differentiation of the AML cells (Supplementary Analysis Table 2). These observations support the hypothesis that the observed differences in regulation of mitosis/proliferation are not caused by differences in AML cell differentiation. Previous clinical studies also support the hypothesis that regulation of proliferation/mitosis is important for chemosensitivity of primary human AML cells. First, autonomous *in vitro* proliferation detected with a 6 days [^3^H]-thymidine incorporation assay similar to our present suspension culture assay (Supplementary Analysis Table 2) was associated with an adverse prognosis in a clinical study including AML patients below 60 years of age and receiving intensive chemotherapy [87]. The same was observed in another study using a 7 days colony formation assay [88] and in a study of cytokine-dependent proliferation [89]. Third, growth of leukemic cells after subcutaneous inoculation in immunodeficient mice is also associated with poor clinical outcome [90] and the same is true for primary human AML cells capable of long-term *in vitro* proliferation in suspension cultures prepared in cytokine-supplemented medium alone without stromal cell support [60]. Even though most of these observations were made for young AML patients, they illustrated that differences in the regulation of AML cell proliferation were associated with differences in prognosis/chemosensitivity, and the differences in molecular regulators of proliferation/mitosis detected in our present study may therefore reflect age-dependent contributions to a molecular profile that is important for the regulation of both mitosis/proliferation and clinical chemosensitivity.

Targeting of the cytoskeleton is regarded as a possible therapeutic strategy in human AML, and several Aurora kinase inhibitors are now in clinical trials for various malignancies [91, 92]. However, despite these clinical studies very few previous investigations have focused on the cytoskeleton in primary AML cells [93–95]. To the best of our knowledge the present study is the first to give a broad and detailed characterization of the cytoskeleton in primary AML cells including a description of patient heterogeneity and possible associations with chemoresistance and/or aging. Our present observations suggesting that the cytoskeletal function is important for chemosensitivity are further supported by a recent study where the development of chemoresistant AML relapse after intensive chemotherapy was associated with altered expression and/or phosphorylation of cytoskeletal proteins [66].

Transcriptional networks are altered in AML patients by several mechanisms that include transcription factor dysregulation by mutation or by translocation or downstream of signaling pathways [96]. We observed higher levels of several transcription repressors (e.g. RERE and ZMYND8) and lower phosphorylation of mRNA stabilization/splicing proteins (e.g. SRRM1/2) in elderly low-risk patients (Figure 3B, 3C). A lower phosphorylation of the same and different RNA processing proteins was also detected (e.g. SRRM1 and serine/arginine-rich splicing factor 11, SRSF11) in high-risk patients when they were compared to all low-risk patients (Figure 5C). Several transcriptional networks of different AML subtypes have been recently described as well as their required role for tumor maintenance and targeting of these altered networks might offer new therapeutic approaches to eliminate the subsistence program of AML cells [97].

Our phosphoproteomics analysis showed that the CDK family appeared to be more activated in younger low-risk patients, especially CDK1 and CDK2 with 110 and 88 substrate counts, respectively, according to the KSEA (Figure 3D). Among these substrates we identified FOXK2 and RB1. FOXK2 is hyperphosphorylated during mitosis by CDK1 and, to a lower extent, CDK2 [98]. The underphosphorylated, active form of RB1 interacts with transcription factor E2F1 (E2F1) and represses its transcription activity, leading to cell cycle arrest [99]. As RB1 was more phosphorylated in younger low-risk patients, E2F1 might be more active and induce myeloid cell-cycle progression [100]. Thus, our results suggest a tight control of the cell-cycle progression in younger low-risk patients.

Our studies also suggest that other biological characteristics of the leukemic cells also differ between elderly low-risk and younger low-risk patients. This includes molecules of the p53 signaling pathway (Figure 3B), which can also be altered as a part of the aging process [101–103]. G2 and S phase-expressed protein 1 (GTSE1), a protein that regulates microtubules (MT) stability during mitosis by inhibiting the mitotic centromere-associated kinesin (MCAK) MT depolymerase activity [104], appeared more phosphorylated in younger low-risk patients.

The regulated phosphoproteome of high-risk patients was involved in apoptotic execution, mitotic prophase, cell cycle progress and RNA processing (Figure 5B–5D). The higher phosphorylation of CDK1 and CDK2 at T14 and Y15, identified by general phosphoenrichment and phosphotyrosine immunoaffinity enrichment, and the higher number of CDK1 substrates identified by KSEA revealed a pivotal performance of these kinases in high-risk patients. Among these substrates, we identified NUMA1, a MT-binding protein that plays a role in the formation and maintenance of the spindle poles and the alignment and the segregation of chromosomes during mitotic cell division [105]. Phosphorylation and dephosphorylation of this protein determine its enrichment at the cell cortex and its association with the dynein-dynactin complex.

The phosphoproteomic analysis of both comparisons of the present study shows that CDKs seem to be activated in both younger low-risk and high-risk patients and target different substrates that might influence prognosis in AML. Moreover, the phosphorylation of CDK1/2 T161 and Y15 active sites was more heterogeneous among high-risk than among low-risk patients (Supplementary Figure 5). We have previously shown that CDKs were also more activated at the time of first diagnosis for patients that relapsed within a 5-year follow-up after intensive and potentially curative therapy [65]. Taking together, CDKs play determinant roles in AML prognosis and relapse, and the prognostic impact seems to differ between patient subsets and depends on the genetic/biological context.

## CONCLUSIONS

Our comparison of elderly low-risk and younger low-risk AML patients suggest that ALDH2 levels, cytoskeletal modulation and altered transcriptional regulation are consistent with an effect of aging on leukemogenesis and chemosensitivity in human AML. The role of kinases such as CDKs on age and disease seems to depend on the biological context and to differ between patient subsets. Elderly patients with high-risk AML seem to differ from all younger patients with low-risk disease with regard to the same cellular processes, even though the molecular mechanisms differ, and few other single molecules (e.g. LSP1) were identified in the two comparisons. Thus, our study suggests age-dependent alterations contributes to chemoresistance in human AML.

## MATERIALS AND METHODS

### Selection of patients and preparation of patient cells

All cell samples in the present study were derived at the first time of diagnosis before start of any antileukemic treatment. Our institution is responsible for diagnosis and treatment of patients with AML in a defined geographical area, and the patients included in our present study represent all patients from this area during a defined time period that fulfilled defined criteria established before start of the study. First, to ensure a high quality of the analyzed samples with at least 95% AML cells we included only patients with high levels of peripheral blood AML cells, i.e. at least 10 x 10^9^ total leukocytes and at least 80% of these circulating leukocytes being leukemic cells. Second, among these consecutive patients with high levels of circulating AML cells, we selected all patients who fulfilled predefined genetic criteria for adverse and favorable prognosis as can been seen from Supplementary Table 1. These criteria are based on the European Leukemia Net (ELN) guidelines [1]. The ELN classification of AML is based on karyotyping together with molecular-genetic analyses, and patients are classified as having favorable, intermediate or adverse prognosis. This risk stratification does not take into account other pretreatment risk factors like peripheral blood blast count at the time of diagnosis, previous hematological malignancy (i.e. myelodysplastic syndrome, chronic myelomonocytic leukemia or chronic myeloproliferative neoplasia) and previous exposure to cytotoxic therapy for other diseases [1]. The classification does not take into account response to first induction cycle and minimal residual disease (MRD) after remission induction either [1, 45, 106, 107].

The patients with adverse prognosis included ten patients with complex karyotype and five patients with either monosomal karyotype, del 5 or -7. These adverse prognosis patients represent all patients who fulfilled the cytogenetic criteria for high-risk disease. Patients were screened for *FLT3* and *NPM1* mutations but additional mutational analyses for identification of additional adverse prognosis patients were not available during the defined time period, and for this reason these adverse prognosis patients were selected based on cytogenetic criteria alone. These patients will be referred to as high-risk patients. The median age of this patient group is 74 years.

The patients with favorable prognosis included all patients with the cytogenetic abnormalities inv(16), t(16;16) and t(8;21). We also included all patients with normal karyotype, wild-type *FLT3* and *NPM1* insertion as well as one patient with normal karyotype, low ratio of internal tandem duplication mutation of *FLT3*, *FLT3*-ITD, and *NPM1* insertion (Supplementary Table 1, patient F17) and another patient with *CEBPA* mutation (Supplementary Table 1, patient F18). Analysis of the *FLT3*-ITD ratio was not available as a routine analysis during this time period and only one patient could be classified as favorable based on these criteria (Supplementary Table 1). These patients will be referred to as low-risk patients. The median age of this patient group is 64.5 years.

More characteristics of the high-risk and low-risk patient groups are summarized in Tables 1, 2, and more detailed comparisons are presented in Supplementary Tables 1, 2. All patients were Caucasians.

As we included patients with high levels of circulating AML blasts at the time of first diagnosis, we could prepare highly enriched AML cell populations (>95% purity) by a standardized method based on density gradient separation alone (for a detailed discussion see references [53, 54, 65]). All samples were cryopreserved by using the same standardized method. The patient samples included in our study did not differ significantly with regard to peripheral blood blast count or storage time in liquid nitrogen.

### Patient grouping for MS-based proteomic and phosphoproteomic analysis

In order to compare patient groups with different cytogenetics-based prognosis, we selected 15 high-risk and 18 low-risk patients for subsequent proteomics analyses. The difference of the median age between the two groups was statistically significant (*P*= 0.005, Table 1). In order to study the influence of age in low-risk patients, we further divided the 18 patients in two subgroups of nine patients each, according to an age threshold of 65 years at the time of diagnosis, i.e. the elderly low-risk (median age of 68 years) and the younger low-risk (median age of 47 years). This threshold was chosen because it corresponds to the median age of patients at the first diagnosis of AML and it has also been used to distinguish between young/middle aged and elderly patients (i.e. not fit for the most intensive chemotherapy) in previous clinical studies [1, 108, 109]. The difference of the median age between the two subgroups was statistically significant (*P* <0.0001). This and other patient characteristics of the low-risk subgroups are shown in Table 2.

### Peptide preparation

Our standard sample preparation of patient cell lysate in 4% sodium dodecyl sulfate (SDS)/0.1 M Tris-HCl (pH 7.6), the filter-aided sample preparation (FASP) procedure with AML patient samples and the immobilized metal affinity chromatography (IMAC) for phosphopeptide enrichment have been described previously [65, 110]. In short, 20 μg of each patient lysate was mixed with 10 μg of an AML-specific super-SILAC mix [111] for proteomic analyses, and digested according to the standard FASP protocol [110, 112]. The super-SILAC spiked peptide samples were fractionated using styrenedivinylbenzene-reversed phase sulfonate (SDB-RPS) plugs (Empore Discs, 3M) for proteome analysis [113]. The phosphoproteomics samples (226-2506 μg) were mixed with the super-SILAC mix at 1:2 ratio (w:w; super-SILAC mix:AML patient sample), FASP processed and enriched using the IMAC procedure. Extra labeled peptides samples (600-1479 μg) were also FASP prepared for phosphotyrosine immunoprecipitation (IP). IP was performed using PTMScan pTyr antibody beads (p-Tyr-1000; Cell Signaling Technology) according to the manufacturer’s protocol. All the peptides samples were brought to equal volumes of binding buffer before antibody incubation. Eluted Tyr-phosphopeptides were cleaned up with 3-C18-disks-stage tips before MS analysis.

### LC-MS/MS measurements

Peptide sample preparation prior to proteome and phosphoproteome analysis and settings of the LC-MS/MS runs on a Q Exactive HF Orbitrap mass spectrometer coupled to an Ultimate 3000 Rapid Separation LC system (Thermo Scientific) were carried out as described earlier [65]. Tyr-phosphopeptides were pre-concentrated on a 2 cm × 75 μm ID Acclaim PepMap 100 trapping column and separated on a 25 cm × 75 μm ID EASY-spray PepMap RSLC analytical column (Thermo Scientific). The Tyr-phosphopeptides were eluted during a 105 min binary gradient with solvent A (0.1% formic acid) and solvent B (0.1% formic acid/acetonitrile). The gradient started at 5% B from 0–5 min and increased to 7% B from 5–5.5 min, then to 22% B from 5.5–65 min, to 35% B from 65–87 min, and to 90% B from 87–92 min. Hold at 90% from 92-102 min, then ramped to 5% B from 102–105 min. The Q Exactive HF mass spectrometer was operated in data dependent acquisition (DDA) mode. Full MS scans (scan range 375–1500 m/z) were acquired in profile mode with a resolution *R* = 60 000 using an AGC target value of 3 × 10^6^ charges. MS/MS scans were acquired in profile mode for the top 10 precursors. The AGC target value was set to 1 × 10^5^ charges with a maximum injection time of 110 ms and a resolution *R* = 60 000. The normalized collision energy was 28 and the isolation window was 1.2 m/z with null m/z offset. The dynamic exclusion lasted for 20s.

The super-SILAC proteomic, phosphoproteomic and Tyr-phosphoproteomic samples were analyzed as three separate experiments in a controlled randomized order (i.e. samples from each patient group were distributed equally over the analysis sequence) with a LC-MS quality control (HeLa protein digest) run approximately every 10 patient samples.

### Data analysis

LC-MS/MS raw files were processed with MaxQuant software version 1.5.2.8 [114, 115]. The spectra were searched against the concatenated forward and reversed-decoy Swiss-Prot Homo sapiens database version 2018_02 using the Andromeda search engine [116]. The Perseus 1.6.1.1 platform was used to analyze and visualize protein groups and phosphosites [117]. MaxQuant-normalized SILAC ratios were inverted, log_2_ transformed and normalized again using width adjustment. Hierarchical clustering of significantly differential proteins and phosphosites was done with Perseus using the Pearson correlation function and complete linkage. Volcano plots were done with Prism8 (GraphPad). GO and KEGG pathways analysis was performed using a GO tool [118]. The most significantly over-represented GO and KEGG pathways terms with *P* <0.05 were displayed in bar or scatter plots in Prism8. The amino acid distribution surrounding the phosphosites was analyzed using iceLogo (*P*= 0.05) with the sequence windows obtained in the MaxQuant-generated phosphosite output file [39]. Sequence logo analyses from a small number of phosphopeptide sequences were generated with WebLogo [34]. Kinase activity estimates were inferred by the KSEA App. [35, 36]. Regulated and unregulated phosphosites were analyzed with the PhosphoSitePlus [119] and NetworKin [120] databases using a substrate count and a NetworKin score cutoff of 5. Kinase activation loop analysis was performed with the tools for phosphoproteomics data analysis at http://phomics.jensenlab.org. PPI networks were obtained by using the STRING database version 11.0 with interactions derived from experiments and databases at a high confidence score of 0.7 [121]. Networks were visualized using the Cytoscape platform version 3.3.0 [122]. The ClusterONE plugin was used to identify protein groups of high cohesiveness [123]. Reactome term enrichment and visualization of hit pathways were performed using the STRING app (1.5.1) [124] and the ReactomeFIViz app (7.2.3) [125, 126], respectively. Causal relationships between phosphoproteins were studied with the SIGnaling Network Open Resource (SIGNOR) 2.0 [38]. Venn diagrams were made with Biovenn [127].

### Western blotting

Western blotting from nine high-risk and nine low-risk patient cells were performed. Twenty μg of each SDS-based cell lysate was loaded on a NuPAGE 4-12% Bis-Tris protein gel (ThemoFisher Scientific) and transferred onto a nitrocellulose membrane (Amersham Protran, GE Healthcare Life Sciences) in an XCell II Blot Module (ThermoFisher Scientific). Antibodies were purchased from Cell Signaling Technology and Abcam. They were used according to manufacturer’s guidelines. Chemiluminescence was developed with SuperSignal West kits (Thermo Scientific) and measured on a LAS-3000 imager (Fujifilm). Band intensities for each protein were determined by densitometry software Image J [128]. Band intensities of a protein spotted at approximately 62 kDa on Ponceau-stained membranes were used for normalization.

### Statistical analysis

Proteins and phosphosites (localization probability >0.75) with at least five individual SILAC ratios in each patient group were selected for two-sample unequal variance *t*-test and *Z*-statistics [129] to find significantly different FC for proteins and phosphosites between the different patient groups. Tyrosine phosphoproteomic data were tested for significance using linear models for microarray data (LIMMA) using the VSClust app [130]. Data from Western blotting bands were expressed as the median ± 95% confidence interval. Statistical analysis was performed using the Mann-Whitney test in Prism8.

### Ethics statement

Primary AML cells were collected from AML patients after written informed consent in accordance with the Declaration of Helsinki. The storage of cells in our biobanks (REK 1759/2015) and the use of cells in the present project (REK 305/2017) were approved by the Regional Ethics Committee.

### Data availability

The LC-MS/MS raw files and MaxQuant output files have been deposited to the ProteomeXchange consortium via the PRIDE partner repository [131, 132] with dataset identifier PXD019785.

## Supplementary Material

Supplementary Figures

Supplementary Tables 1, 2 and 3

Supplementary Table 4

Supplementary Table 5

Supplementary Table 6

Supplementary Table 7

Supplementary Table 8

Supplementary File 1

Supplementary File 2

Supplementary File 3

Supplementary File 4

Supplementary File 5

Supplementary References

Supplementary Data Analysis
