# Supplementary material for: Biological characteristics of aging in human acute myeloid leukemia cells: the possible importance of aldehyde dehydrogenase, the cytoskeleton and altered transcriptional regulation
Source: Aging (Albany NY). 2020 Dec 20;12(24):24734–77. doi: 10.18632/aging.202361 (PMC7803495; doi:10.18632/aging.202361)
Supplement: Supplementary Figures [file aging-12-202361-s001.pdf]

SUPPLEMENTARY FIGURES

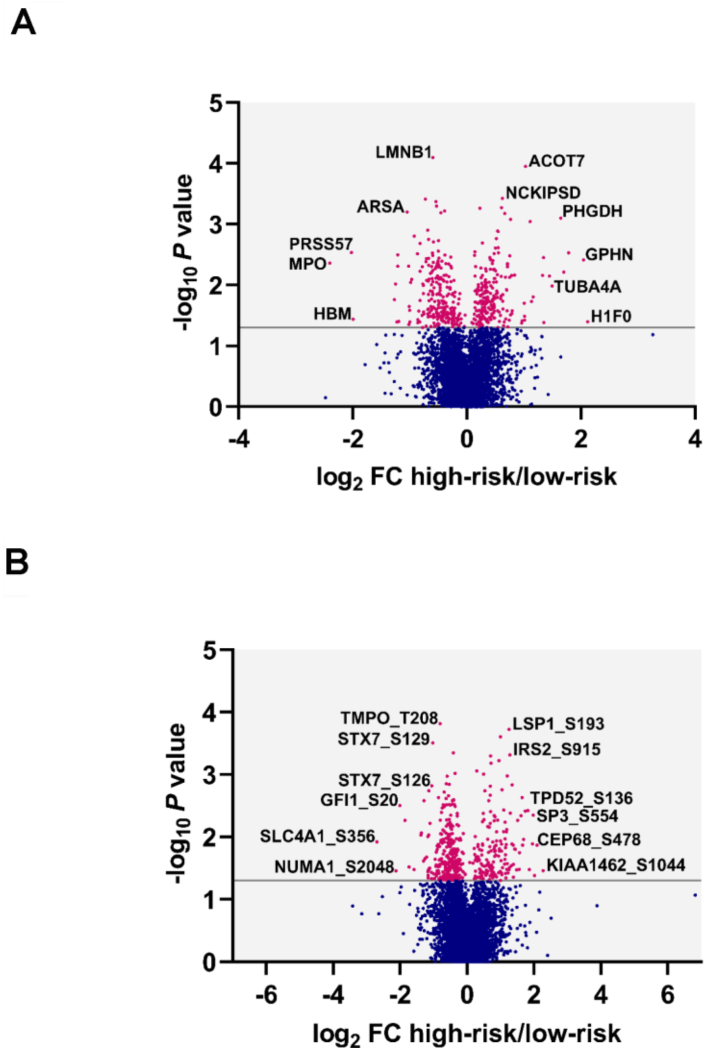

Supplementary Figure 1. Volcano plot analyses of the data from the high-risk *vs* low-risk cohort. Points (in magenta) above the non-axial horizontal grey line represent proteins or phosphosites with significantly different abundances or phosphorylation ($P < 0.05$), respectively. (**A**) All proteins with at least 5 quantitative values in each group were used in the analysis. (**B**) All phosphosites with at least 5 quantitative values in each group were used in the analysis.

A

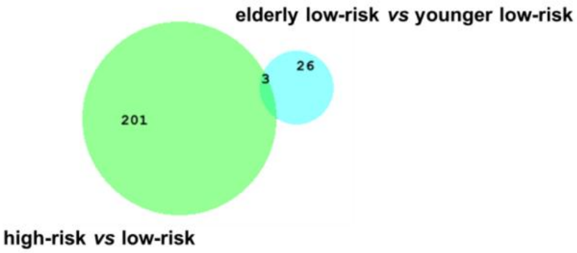

Protein	FC elderly low-risk *vs* younger low-risk	FC high-risk *vs* low-risk
ALDH2	1.9985	1.7844
UFSP2	0.4986	-0.4933
BAG2	-0.9124	1.4456

B

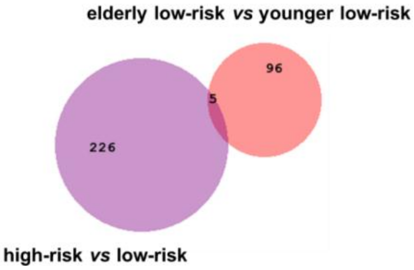

Phosphosite	FC elderly low-risk *vs* younger low-risk	FC high-risk *vs* low-risk
LSP1_S177	1.0748	0.9508
TMPO_S159	0.6759	-1.1676
CDK1_Y15	-1.0066	0.7270
CDK2_Y15	-1.0066	0.7270
ESCO2_S75	-1.7257	1.4278

Supplementary Figure 2. Venn diagrams of regulated proteins and phosphoproteins in the studies of elderly low-risk *vs* younger low-risk, and high-risk *vs* all low-risk patients. (**A**) Overlap of 29 and 204 regulated proteins in the elderly low-risk *vs* younger low-risk, and high-risk *vs* low-risk studies, respectively, with table of the fold change (FC) values of overlapped proteins obtained in both studies. (**B**) Overlap of 101 and 231 unique differentially regulated phosphorylation sites in the elderly low-risk *vs* younger low-risk, and high-risk *vs* low-risk studies, respectively, with table of the FC values of overlapped phosphosites obtained in both studies.

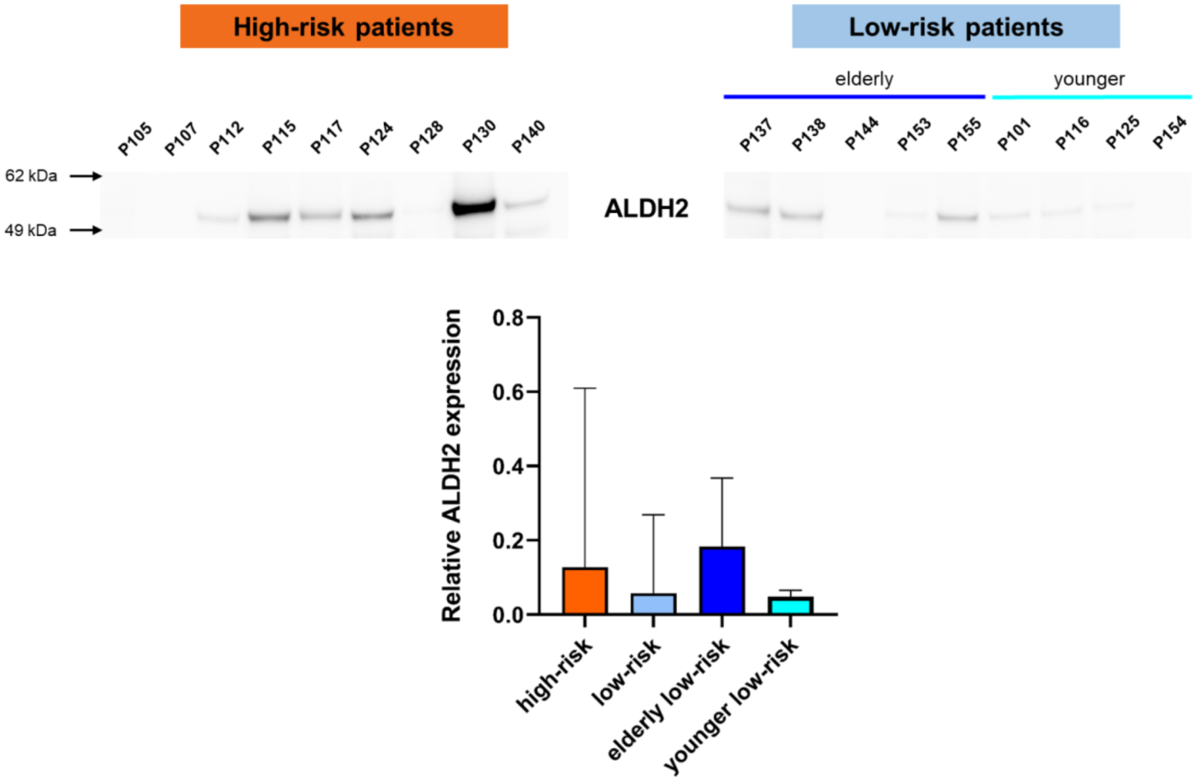

Supplementary Figure 3. Western blots of sample lysates from nine high-risk and nine low-risk patients to study the activity of ALDH2. Band intensities of ALDH2 protein were normalized before statistical analysis. Data from Western blot bands were expressed as the median ± 95% confidence interval in the bar plots. Band intensities from the high-risk and low-risk as well as from elderly low-risk and younger low-risk samples were compared using the Mann-Whitney test. None of the comparisons were statistically significant. Western blots were not replicated.

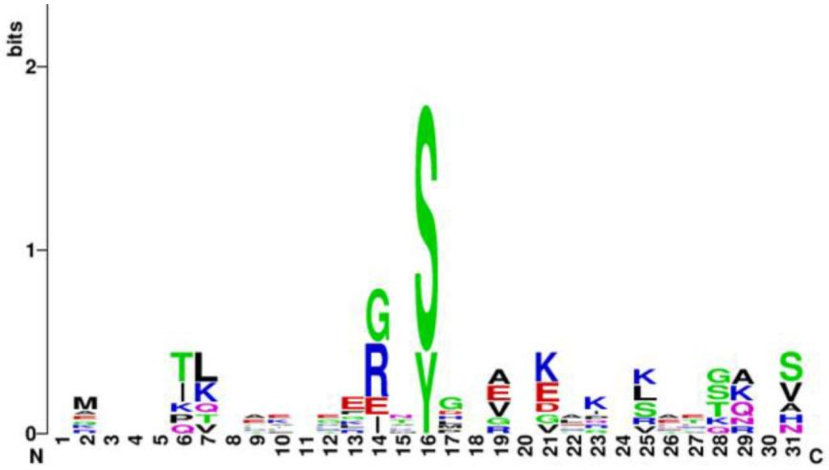

Supplementary Figure 4. Sequence logo analysis of the phosphoprotein Cluster 1 showed in Figure 5C on the main text. Thirty-one amino acid sequence windows surrounding the phosphorylation sites (located on position 16 on the x-axis) described in the protein cluster.

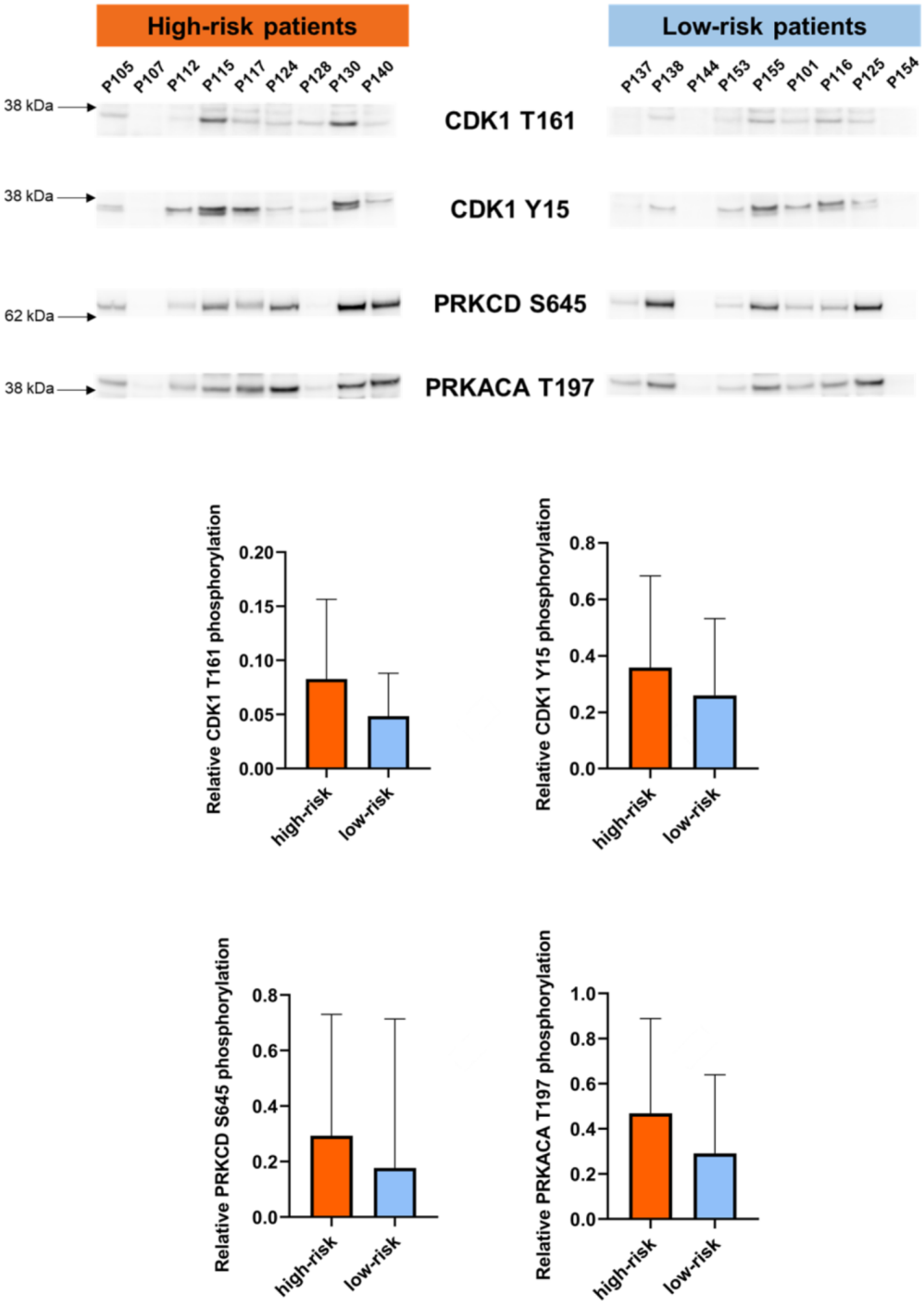

Supplementary Figure 5. Western blots of sample lysates from nine high-risk and nine low-risk patients to study the activity of CDK1, PRKCD and PRKACA. Band intensities of phosphorylated proteins were normalized before statistical analysis. Data from Western blot bands were expressed as the median ± 95% confidence interval in the bar plots. Band intensities from the high-risk and low-risk samples were compared using the Mann-Whitney test. None of the comparisons were statistically significant. Western blots were not replicated.
