# Supplementary material for: Biological characteristics of aging in human acute myeloid leukemia cells: the possible importance of aldehyde dehydrogenase, the cytoskeleton and altered transcriptional regulation
Source: Aging (Albany NY). 2020 Dec 20;12(24):24734–77. doi: 10.18632/aging.202361 (PMC7803495; doi:10.18632/aging.202361)
Supplement: Supplementary Tables 1, 2 and 3 [file aging-12-202361-s002.pdf]

SUPPLEMENTARY TABLES

Supplementary Table 1. Clinical and biological characteristics of individual patients included in the study.

ID	Sex	Age	FAB	CD34	Karyotype	*FLT3*	*NPM1*
**High-risk patients, i.e. adverse cytogenetic abnormalities**							
A1-P105	F	64	M1	+	Complex[2]	WT	WT
A2-P112	F	51	M0	+	Complex	WT	WT
A3-P114	M	72	M1	+	Complex	WT	-
A4-P117	F	64	M1	+	Complex	ITD	WT
A5-P122	M	84	M1	+	Complex	WT	WT
A6-P130	M	78	M0	-	Complex	WT	WT
A7-P133	M	74	M4	-	Complex	WT	-
A8-P134	M	80	M1	+	Complex	-	-
A9-P140	F	50	M2	+	Complex	WT	WT
A10-P128	M	77	M2	+	Complex	ITD	WT
A11-P115	F	87	M0	+	del 5	WT	WT
A12-P107	M	73	M2	+	Monosomal	ITD	WT
A13-P123	M	81	M2	-	-7	WT	WT
A14-P124	M	76	M5	+	del 12, -7	WT	WT
A15-P129	M	63	M5	+	-7	WT	WT
**Low-risk patients, i.e. favorable genetic abnormalities**							
F1-P110	F	67	M4	+	t(16;16), +22	-	-
F2-P116	M	36	M5	+	inv16, +8, +22	ITD	WT
F3-P139	M	66	M5	+	t(16;16)	WT	WT
F4-P141	M	79	M2	-	inv16, del 7	WT	WT
F5-P127	M	41	M1	+	t(8;21), del 9, -3, -20, -22	WT	WT
F6-P131	F	33	M1	+	t(8;21)	WT	WT
F7-P137	F	74	M4	+	t(8;21)	ITD	WT
F8-P144	F	66	M4	+	t(8;21)	WT	WT
F9-P154	M	47	M4	-	t(8;21)	WT	WT
F10-P101	M	50	M2	-	Normal	WT	INS
F11-P118	F	61	M5	-	Normal	WT	INS
F12-P120	F	68	M5	-	Normal	WT	INS
F13-P125	M	64	M5	-	Normal	WT	INS
F14-P138	M	65	M4	-	Normal	WT	INS
F15-P143	M	64	M1	+	Normal	WT	INS
F16-P153	F	70	M4	+	Normal	WT	INS
F17-P155[3]	F	71	M0	-	Normal	ITD^{low}	INS
F18-P142[4]	M	33	M2	+	Normal	WT	WT

The table presents individually patient characteristics (sex, gender), morphological (FAB classification) and molecular signs of differentiation (CD34 expression), karyotype and *FLT3*/*NPM1* mutational status[1].

[1]Abbreviations: FAB, French-American-British; INS, a 4 bp-insertion/duplication; ITD, internal tandem duplication; WT, wild-type; -, not determined.

[2]Defined as ≥ 3 cytogenetic abnormalities.

[3]The patient had a low ITD ratio and could therefore be classified as having a favorable prognosis. He was initially classified as ITD negative but later reclassified based on a new analysis.

[4]This patient has *CEBPA* mutation.

Supplementary Table 2. Patient treatment and survival.

ID	Treatment[1]	Survival (months)
	**High risk patients, i.e. adverse cytogenetic abnormalities**	
A1-P105	Best supportive care	<1
A2-P112	AML-stabilizing therapy based on ATRA and valproic acid	3
A3-P114	AML-stabilizing therapy based on ATRA and valproic acid	13
A4-P117	Best supportive care	3
A5-P122	AML-stabilizing therapy based on ATRA and valproic acid	5
A6-P130	Valproic acid plus hydroxyurea	3
A7-P133	Best supportive care	<1
A8-P134	AML-stabilizing therapy based on ATRA and valproic acid	1
A9-P140	Intensive chemotherapy followed by allogeneic SCT	>46[2]
A10-P128	Best supportive care	<1
A11-P115	ATRA, valproic acid, low-dose cytarabine	<1
A12-P107	Best supportive care	2
A13-P123	ATRA, valproic acid, low-dose cytarabine	16
A14-P124	Valproic acid plus hydroxyurea	6
A15-P129	Best supportive care	6
	**Low risk patients, i.e. favorable genetic abnormalities**	
F1-P110	Best supportive care	<1
F2-P116	Death from acute GVHD after allogeneic SCT in second CR	25
F3-P139	ATRA, valproic acid, low-dose cytarabine	<1
F4-P141	Valproic acid plus hydroxyurea	3
F5-P127	Lost from follow-up	-
F6-P131	Intensive chemotherapy followed by autologous SCT	>56
F7-P137	Azacitidine	3
F8-P144	Intensive induction and consolidation chemotherapy	>38
F9-P154	High-dose chemotherapy	>144
F10-P101	One cycle of intensive induction therapy with CR, no further chemotherapy due to severe toxicity	28
F11-P118	Intensive induction therapy with CR, toxic death during consolidation	2
F12-P120	Death from hyperleukocytosis	<1
F13-P125	Intensive induction with CR, toxic death during consolidation therapy	2
F14-P138	Intensive chemotherapy followed by autologous SCT, non-relapse death	14
F15-P143	Intensive induction with CR, toxic death during consolidation chemotherapy	2
F16-P153	AML-stabilizing therapy based on ATRA and valproic acid	2
F17-P155	Best supportive care	2
F18-P142	Intensive chemotherapy followed by autologous SCT	>44

[1]Abbreviations: ATRA, all-trans retinoic acid; CR, complete transmission; GVHD, Graft versus host disease; SCT: stem cell transplantation.

[2]The sign > means that the patient is still alive without relapse.

Supplementary Table 3. Classification of differentially expressed proteins and phosphorylated phosphoproteins found in the comparative studies between nine elderly low-risk and nine younger low-risk patients based on hallmarks of aging as explained in previous publications [1–3].

Classification	Genomic stability, DNA repair	Mitosis, cell cycle	Epigenetics, chromatin, transcription, RNA splicing, ribosome	Protein homeostasis, metabolism, protein modification	Mitochondria, metabolism	Cytoskeleton, actin polarization	Intracellular ER[1]-Golgi trafficking	Intracellular signaling	Extracellular secretion	Membrane structure	Tumor suppressor	Regulation of senescence	Regulation of apoptosis
Altered protein level in low-risk patients	APTX<br>CAAP1<br>NME3<br>POLB	CDC27<br>KIAA1279<br>NUMB	CHD2<br>MARS2<br>MINA<br>RPS6KA4<br>WARS2	ASPH<br>BAG2<br>CDC27<br>KIAA1279<br>NME3<br>PDP1<br>PTPLAD2<br>STX7<br>UFSP2<br>NUMB	ALDH2<br>CLC<br>COX6A1<br>KIAA1279<br>MARS2<br>NXT2<br>PDF<br>PDP1<br>PTDSS1<br>WARS2	CAP1<br>GIT1<br>SKAP2	KIAA1279<br>NXT2<br>STX7	GIT1<br>MOB4<br>NENF<br>SKAP2	NENF	PTDSS1<br>NUMB	PTPLAD2		CAAP1
Increased phosphorylation in elderly low-risk patients	RMI1<br>TOX4<br>TP53BP1	CBX1<br>HECA<br>TMPO	ARID1A<br>AHNAK<br>CBX1<br>DDX41<br>IRF2BP1<br>KANSL1<br>KDM3B<br>RERE<br>RPS6KA4<br>SP100<br>TCEAL3<br>TMPO<br>TOX4<br>TP53BP1<br>ZMYND8	DTNBP1		ARIDA1<br>FAM21C<br>FNBP1L<br>LSP1<br>MYO18A<br>REPS1	BIN1<br>REPS1<br>SEC61B<br>WDR44	DOCK5<br>FAM65B	LILRB3		BIN1<br>TP53BP1		BIN1<br>RERE
Decreased phosphorylation in elderly low-risk patients	LIG1	CDK1<br>CDK2<br>ESCO2<br>FAM83H<br>FOXK2<br>GSG2<br>INCENP<br>RB1	AATF<br>ATAD2<br>BCLAF1<br>CHAF1A<br>CHD4<br>CHD9<br>CNP<br>EIF3F<br>ESCO2<br>FOXK2<br>GSG2<br>HIST1H1D<br>IGF2BP1<br>ING5<br>KMT2A<br>NPM1<br>PPIG<br>PRPF40A<br>RPRD2<br>RRP1B<br>RRP36<br>SRRM1<br>SRRM2	ATAD2<br>BCLAF1<br>EEF2<br>HSP90AB1<br>NPM1<br>POLA2<br>PPP6R3	CDK2<br>ESYT2<br>FOXK2<br>PCYT1B	IGF2BP1<br>SCRIB		IGF2BP1<br>IRS2			AATF<br>EIF3F<br>ING5<br>RB1<br>SCRIB	CDK2	AATF<br>BCLAF1<br>FOXK2
N[2]	8	14	43	18	14	11	7	8	2	2	8	1	6

The table is based on information from the Gene database and selected references are from the PubMed database as described more in detail in Supplementary Tables 4–6.

Abbreviations: [1]ER, endoplasmic reticulum; [2]N, number of proteins.
