# Supplementary material for: Biological characteristics of aging in human acute myeloid leukemia cells: the possible importance of aldehyde dehydrogenase, the cytoskeleton and altered transcriptional regulation
Source: Aging (Albany NY). 2020 Dec 20;12(24):24734–77. doi: 10.18632/aging.202361 (PMC7803495; doi:10.18632/aging.202361)
Supplement: Supplementary Table 4 [file aging-12-202361-s003.docx]

**Supplementary Table 4.** **Proteins with significantly altered level in low-risk patients**

The proteins are listed in alphabetic order according to the name of the encoding gene. Proteins that are important in hematopoietic stem cells are highlighted in yellow. The table is based on information from the Gene database and selected references are from PubMed

(FC stands for protein expression fold change)

| **Protein/FC elderly low-risk *vs* younger low-risk** | **Comment** | **Reference(s)** | **Keywords** |
| --- | --- | --- | --- |
| ALDH2/2.00 | **Aldehyde dehydrogenase 2 family member.** Aldehyde dehydrogenase is the second enzyme of the major oxidative pathway of alcohol metabolism. Two major isoforms of aldehyde dehydrogenase, cytosolic and mitochondrial, can be distinguished. This gene encodes a mitochondrial isoform | [4] | Mitochondria,  metabolism |
| APTX/-0.41 | **Aprataxin.** This gene encodes a member of the histidine triad (HIT) superfamily. The encoded protein may play a role in single-stranded DNA repair through its nucleotide-binding activity and its diadenosine polyphosphate hydrolase activity | [5] | DNA repair |
| ASPH/0.67 | **Aspartate beta-hydroxylase.** This protein is thought to be important in calcium homeostasis. The longest isoforms hydroxylate aspartic acid or asparagine residues. Other isoforms lack the hydroxylase domain, and some have been localized to the endoplasmic |  | Protein hydroxylation,  calcium activation |
| BAG2/-0.91 | **BAG cochaperone 2.** BAG proteins compete with Hip for binding to the Hsc70/Hsp70 ATPase domain and promote substrate release. The predicted BAG2 protein contains 211 amino acids. The BAG domains of BAG1, BAG2, and BAG3 interact specifically with the Hsc70 ATPase domain in vitro and in mammalian cells. All 3 proteins bind with high affinity to the ATPase domain of Hsc70 and inhibit its chaperone activity in a Hip-repressible manner |  | Protein stability |
| CAAP1/0.72 | **Caspase activity and apoptosis inhibitor 1.** This protein has been implicated as a negative regulator of the intrinsic apoptosis pathway by modulating caspase expression and activity | [6] | Apoptosis |
| CAP1/-0.53 | **Cyclase associated actin cytoskeleton regulatory protein 1.** The protein involved in the cyclic AMP pathway and also interacts with CAP2 (cyclase associated actin cytoskeleton regulatory protein 2) and actin |  | Actin |
| CDC27/-0.42 | **Cell division cycle 27.** The protein is a component of the anaphase-promoting complex (APC) that catalyzes the formation of cyclin B-ubiquitin conjugate, which is responsible for the ubiquitin-mediated proteolysis of B-type cyclins. This protein was shown to interact with mitotic checkpoint proteins |  | Mitosis,  proteostasis? |
| CHD2/-0.44 | **Chromodomain helicase DNA binding protein 2.** CHD genes alter gene expression possibly by modification of chromatin structure thus altering access of the transcriptional apparatus to its chromosomal DNA template | [7] | Transcription |
| CLC/0.97 | **Charcot-Leyden crystal galectin.** Lysophospholipases are enzymes that act on biological membranes to regulate the multifunctional lysophospholipids. This protein is a lysophospholipase. It hydrolyzes lysophosphatidylcholine to glycerophosphocholine and a free fatty acid. This protein may also possess carbohydrate-binding activities |  | Lipid metabolism |
| COX6A1/0.56 | **Cytochrome c oxidase subunit 6A1.** Cytochrome c oxidase (COX) is the terminal enzyme of the mitochondrial respiratory chain, catalyzes the electron transfer from reduced cytochrome c to oxygen |  | Mitochondria,  metabolism |
| GIT1/0.91 | **GIT ArfGAP 1 (ARF GTPase-activating protein GIT1).** Arf GTPase-activating proteins (Arf GAPs) control the activity of ADP-ribosylation factors (Arfs) by inducing GTP hydrolysis and participate in a diverse array of cellular functions both through mechanisms that are dependent on and independent of their Arf GAP activity. A number of these functions hinge on the remodeling of actin filaments. Accordingly, some of the effects exerted by Arf GAPs involve proteins known to engage in regulation of the actin dynamics and architecture, such as Rho family proteins and nonmuscle myosin 2. The encoded protein interacts with the myosin MYO18A. The related molecule GIT2 is important in aging | [8-10] | Actin,  GTPase,  Rho protein,  MYO18A |
| KIAA1279/0.40 | **Kinesin family binding protein.** This gene encodes a kinesin family member 1 binding protein that is characterized by two tetratricopeptide repeats. The encoded protein localizes to the mitochondria and may be involved in regulating transport of the mitochondria. Kinesis are important for the control of microtubules thereby intracellular cargo trafficking but also for accurate chromosomal segregation during mitosis | [11, 12] | Mitochondria,  mitosis,  intracellular trafficking,  proteostasis |
| MARS2/-1.33 | **Methionyl-tRNA synthetase 2, mitochondrial.** This mitochondrial methionyl-tRNA synthetase protein is encoded by the nuclear genome and imported to the mitochondrion. This protein likely functions as a monomer and is predicted to localize to the mitochondrial matrix. Mutations in this gene are associated with the autosomal recessive neurodegenerative disease spastic ataxia-3 (SPAX3) |  | Mitochondria,  RNA |
| MINA/0.56 | **Ribosomal oxygenase 2.** This protein is a c-Myc target gene that may play a role in cell proliferation or regulation of cell growth. This oxygenase can act as both a histone lysine demethylase and a ribosomal histidine hydroxylase. It is also involved in the demethylation of trimethylated Lys9 on histone H3 (H3K9me3), leading to an increase in ribosomal RNA expression | [13] | Ribosomal RNA expression |
| MOB4/0.40 | **MOB family member 4, phocein.** The protein is a regulator of signaling through the Hippo pathway and may be involved in carcinogenesis | [14, 15] | Intracellular signaling |
| NENF/-0.45 | **Neudesin neurotrophic factor.** This is an extracellularly released protein but also a modulator of intracellular signaling | [16] | Intracellular signaling |
| NME3/0.54 | **NME/NM23 nucleoside diphosphate kinase 3.** This protein is an isoform of the NME family of proteins. NME3 has been implicated in repair of both single- and double-stranded breaks in DNA. This suggests that reduced expression of NME proteins could contribute to the genomic instability that drives cancer progression | [17] | Genome stability |
| NUMB/0.93 | **NUMB endocytic adaptor protein.** The protein plays a role in the determination of cell fates during development. Its degradation is induced in a proteasome-dependent manner by MDM2, it is a membrane-bound protein that associates with EPS15, LNX1, and NOTCH1 |  | Cell division |
| NXT2/-0.41 | **Cytochrome P450 family 2 subfamily C member 19.** Cytochrome P450 proteins are monooxygenases which catalyze many reactions involved in drug metabolism and synthesis of cholesterol, steroids and other lipids. This protein localizes to the endoplasmic reticulum and is known to metabolize many xenobiotics |  | Metabolism,  endoplasmic reticulum |
| PDF/-0.55 | **Peptide deformylase, mitochondrial.** Protein synthesis proceeds after formylation of methionine by methionyl-tRNA formyl transferase (FMT) and transfer of the charged initiator f-met tRNA to the ribosome. The product of this gene, peptide deformylase (PDF), removes the formyl group from the initiating methionine of nascent peptides. In human cells, only mitochondrial proteins have N-formylation of initiating methionines |  | Mitochondria |
| PDP1/0.42 | **Pyruvate dehydrogenase phosphatase catalytic subunit 1.** Pyruvate dehydrogenase (E1) is one of the three components (E1, E2, and E3) of the large pyruvate dehydrogenase complex. Pyruvate dehydrogenase kinases catalyze phosphorylation of serine residues of E1 to inactivate the E1 component and inhibit the complex. Pyruvate dehydrogenase phosphatases catalyze the dephosphorylation and activation of the E1 component to reverse the effects of pyruvate dehydrogenase kinases. Along with the pyruvate dehydrogenase complex and pyruvate dehydrogenase kinases, this enzyme is located in the mitochondrial matrix | [18, 19] | Protein modification,  mitochondria |
| POLB/0.52 | **DNA polymerase beta.** This DNA polymerase is involved in base excision and repair, also called gap-filling DNA synthesis. The protein monomer is normally found in the cytoplasm and translocates to the nucleus upon DNA damage | [20] | DNA repair |
| PTDSS1/-0.52 | **Phosphatidylserine synthase 1.** The protein catalyzes the formation of phosphatidylserine from either phosphatidylcholine or phosphatidylethanolamine. Phosphatidylserine localizes to the mitochondria-associated membrane of the endoplasmic reticulum, where it serves a structural role as well as a signaling role |  | Mitochondria,  membrane structure |
| PTPLAD2/0.61 | **3-hydroxyacyl-CoA dehydratase 4.** The protein contains a characteristic catalytic motif of the protein tyrosine phosphatases (PTPs) family. Members of the PTP family are signaling molecules that regulate a variety of cellular processes. The protein can function as a tumor suppressor | [21] | Tumor suppressor,  protein modification |
| RPS6KA4/0.65 | **Ribosomal protein S6 kinase A4.** This protein is a member of the ribosomal S6 kinase family of serine/threonine kinases. It phosphorylates various substrates, including CREB1 and ATF1. The encoded protein can also phosphorylate histone H3 to regulate certain inflammatory genes |  | Transcription,  Histones,  CREB and ATF1 phosphorylation |
| SKAP2/0.63 | **Src kinase associated phosphoprotein 2.** The protein is an adaptor protein that is thought to play an essential role in the Src signaling pathway. Some reports indicate that this protein inhibits actin polymerization through interactions with actin assembly factors | [22, 23] | Src signaling,  actin |
| STX7/0.43 | **Syntaxin 7.** The protein is a syntaxin family membrane receptor involved in vesicle transport. The encoded protein binds alpha-SNAP, an important regulator of transport vesicle fusion. Along with syntaxin 13, this protein plays a role in the ordered fusion of endosomes and lysosomes with the phagosome |  | Vesicle fusion,  Lysosomes,  protein homeostasis? |
| UFSP2/0.50 | **UFM1 specific peptidase 2.** This gene encodes a highly conserved cysteine protease. The protein cleaves two C-terminal residues from ubiquitin-fold modifier 1, a ubiquitin-like post-translational modifier protein. Activation of ubiquitin-fold modifier 1 by the encoded protein exposes a C-terminal glycine residue that allows interaction with other proteins and transfer to its target protein. An allelic variant of this gene has been associated with Beukes hip dysplasia. Alternative splicing results in multiple transcript variants |  | Protein homeostasis? |
| WARS2/-0.82 | **Tryptophanyl tRNA synthetase 2, mitochondrial.** Aminoacyl-tRNA synthetases catalyze the aminoacylation of tRNA by their cognate amino acid. Because of their central role in linking amino acids with nucleotide triplets contained in tRNAs, aminoacyl-tRNA synthetases are thought to be among the first proteins that appeared in evolution. Two forms of tryptophanyl-tRNA synthetase exist, a cytoplasmic form, named WARS, and a mitochondrial form, named WARS2. This gene encodes the mitochondrial tryptophanyl-tRNA synthetase. Two alternative transcripts encoding different isoforms have been described |  | Mitochondrial  RNA |
