# Supplementary material for: Biological characteristics of aging in human acute myeloid leukemia cells: the possible importance of aldehyde dehydrogenase, the cytoskeleton and altered transcriptional regulation
Source: Aging (Albany NY). 2020 Dec 20;12(24):24734–77. doi: 10.18632/aging.202361 (PMC7803495; doi:10.18632/aging.202361)
Supplement: Supplementary Table 5 [file aging-12-202361-s004.docx]

**Supplementary Table 5.** **Proteins with significantly higher phosphorylation in elderly low-risk patients**

The proteins are listed in alphabetical order according to the name of the encoding gene. Phosphoproteins that are important in hematopoietic stem cells are highlighted in yellow. The table is based on information from the Gene database and selected references are from PubMed

(FC stands for phosphorylation fold change)

| **Protein/phosphorylation site(s)/FC elderly low-risk *vs* younger low-risk** | **Comment** | **Reference(s)** | **Keywords** |
| --- | --- | --- | --- |
| AHNAK/S5762/1.29 | **AHNAK nucleoprotein.** The protein is a large (700 kDa) structural scaffold protein. It may play a role in cell structure and migration/tumor metastasis. A shorter variant encoding a 17 kDa isoform initiates a feedback loop that regulates alternative splicing of this gene |  | Nucleus,  splicing |
| AHNAK/S5752/0.89 |  |  |  |
| AHNAK/S5763/0.84 |  |  |  |
| ARID1A/S363/0.62 | **AT-rich interaction domain 1A.** This gene encodes a member of the SWI/SNF family whose members have helicase and ATPase activities and are thought to regulate transcription of certain genes by altering the chromatin structure around those genes. The encoded protein is part of the large ATP-dependent chromatin remodeling complex SNF/SWI, and through this complex there is a functional interaction involving DOC10 with Rac1 and CDC42 | [24-26] | Transcription,  chromatin,  SNF/SWI,  RAC1, DOCK10, CDC42 |
| BIN1/S267/0.70 | **Bridging integrator 1.** This gene encodes a MYC-interacting nucleocytoplasmic adaptor protein with features of a tumor suppressor. It may be involved in vesicle endocytosis and may interact with dynamin, synaptojanin, endophilin, and clathrin. Certain isoforms activate a caspase-independent apoptotic process |  | Tumor suppressor,  apoptosis,  endocytosis |
| CBX1/S89/0.77 | **Chromobox 1.** This nonhistone protein is a member of the heterochromatin protein family, it is associated with centromeres and interacts with a number of chromatin-associated nonhistone proteins. The protein may play an important role in the epigenetic control of chromatin structure and gene expression |  | Chromatin,  centromeres,  transcription |
| DDX41/S21/1.52 | **DEAD-box helicase 41.** DEAD box proteins are RNA helicases and are important for alteration of RNA secondary structure. Some members of the DEAD box protein family seem to be involved in cell division. This protein interacts with several spliceosomal proteins. DDX41 mutations are observed in germline cells long before MDS presentation. The protein is probably important for leukemogenesis in human AML | [27, 28] | RNA helicase |
| DDX41/S23/1.52 |  |  |  |
| DOCK5/S1789/0.90 | **Dedicator of cytokinesis 5.** This member of the dedicator of cytokinesis protein family that acts as guanine nucleotide exchange factors for small Rho family G proteins. The protein is thought to associate with adaptors CRK and CRKL | [29] | G protein |
| DTNBP1/S316/0.59 | **Dystrobrevin binding protein 1.** This protein plays a role in organelle biogenesis associated with lysosomes. A similar protein in mouse is a component of a protein complex termed biogenesis of lysosome-related organelles complex 1 (BLOC-1) and the dystrophin-associated protein complex (DPC) |  | Lysosome |
| FAM21C/S539/1.22 | **WASH complex subunit 2C.** The WASH complex is important for actin polymerization | [30] | Actin |
| FAM21A/T287/0.80 | **WASH complex subunit 2A** |  |  |
| FAM65B/S37/1.52 | **Rho family interacting cell polarization regulator 2.** This protein is an inhibitor of the small G protein RhoA | [31, 32] | G-protein |
| FNBP1L/S430/1.73 | **Formin binding protein 1 like.** This protein binds CDC42 and N-WASP. It promotes CDC42-induced actin polymerization by activating the N-WASP-WIP complex and, therefore, links cell surface signals to the actin cytoskeleton. Important for aging in hematopoietic stem cells | [29, 33, 34] | CDC42, actin, cytoskeleton |
| HECA/S264/0.85 | **Hdc homolog, cell cycle regulator.** This is a highly basic, cytoplasmic protein that regulates the re-entry of imaginal cells into the mitotic cycle |  | Mitosis |
| HECA/S268/0.85 |  |  |  |
| HECA/S272/0.85 |  |  |  |
| HMHA1/S23/1.07 | **Rho GTPase-activating protein 45.** This protein contains a GTPase activator for the Rho-type GTPases (RhoGAP) domain that would be able to negatively regulate the actin cytoskeleton as well as cell spreading. However, also contains N-terminally a BAR-domin which is able to play an autoinhibitory effect on this RhoGAP activity |  | Cytoskeleton |
| HMHA1/S25/1.07 |  |  |  |
| IRF2BP1/S453/0.63 | **Interferon regulatory factor 2 binding protein 1.** This is a nuclear protein acting as a transcriptional cofactor. It is important for cell survival and differentiation | [35] | Transcription |
| KANSL1/S991/0.84 | **KAT8 regulatory NSL complex subunit 1.** This nuclear protein is a subunit of two protein complexes involved with histone acetylation, the MLL1 complex and the NSL1 complex |  | Chromatin,  histone, acetylation |
| KANSL1/T1003/0.84 |  |  |  |
| KDM3B/S798/0.57 | **JmjC domain-containing histone demethylase 2.** This is a histone demethylase that is important in regulation of H3K4 methylation. It is important for the tumorigenic potential of human colorectal cancer stem cells through Wnt/β-catenin signaling. It is also a regulator of transcription through its interaction with SCAI | [36, 37] | Histone  H3K4-H3K9  demethylation,  transcription |
| LILRB3/S503/1.02 | **Leukocyte immunoglobulin like receptor B3.** The protein is a member of the leukocyte immunoglobulin-like receptor (LIR) family, it binds to MHC class I molecules |  | MHC binding |
| LSP1/S177/1.07 | **Lymphocyte specific protein 1.** This is an intracellular F-actin binding protein. | [38, 39] | Actin |
| MYO18A/S2005/0.81 | **Myosin XVIIIA.** The protein binds GOLPH3, linking the Golgi to the cytoskeleton and influencing Golgi membrane trafficking. It is also part of a complex that assembles lamellar actomyosin bundles. MYO18A has been found in a few different complexes involved in intracellular transport processes. MYO18A is found in a complex with LURAP1 and MRCK that functions in retrograde treadmilling of actin | [10] | Cytoskeleton, Golgi,  actin |
| REPS1/S272/0.63 | **RALBP1 associated Eps domain containing 1.** This signaling adaptor protein interacts with proteins that participate in signaling, endocytosis and cytoskeletal changes. The encoded protein has been found in association with intersectin 1 and Src homology 3-domain growth factor receptor-bound 2-like (endophilin) interacting protein 1 when intersectin 1 was isolated from clathrin-coated pits |  | Endocytosis, intersectin 1,  endophilin |
| RERE/S656/0.67 | **Arginine-glutamic acid dipeptide repeats.** The encoded protein co-localizes with a transcription factor, it associates with a histone deacetylase, function as transcriptional corepressor and its overexpression triggers apoptosis |  | Transcription,  HDAC,  apoptosis |
| RMI1/S284/1.04 | **RecQ mediated genome instability 1.** Repair of DNA double-strand breaks via homologous recombination can produce double Holliday junctions (dHJs) that require enzymatic separation. Topoisomerase IIIα (TopIIIα) together with RMI1 disentangles the final hemicatenane intermediate obtained once dHJs have converged | [40] | DNA repair |
| RMI1/S292/0.73 |  |  |  |
| RPS6KA4/S343/0.76 | **Ribosomal protein S6 kinase A4.** This member of the RSK (ribosomal S6 kinase) family of serine/threonine kinases that phosphorylates various substrates, including CREB1 and ATF1. The encoded protein can also phosphorylate histone H3 |  | CREB1, ATF,  histone |
| RPS6KA4/S347/0.76 |  |  |  |
| SEC61B/S17/0.79 | **SEC61 translocon subunit beta.** The Sec61 complex is the central component of the protein translocation apparatus of the endoplasmic reticulum membrane. Sec61 subunits are also observed in the post-ER compartment |  | Endoplasmic reticulum |
| SP100/S389/1.04 | **SP100 nuclear antigen.** This protein is a component of the PML (promyelocytic leukemia)-SP100 nuclear bodies and binds heterochromatin proteins |  | Chromatin |
| TCEAL3/S30/1.02 | **Transcription elongation factor A like 3.** This protein is a member of the transcription elongation factor A (SII)-like (TCEAL) family. Members of this family may function as nuclear phosphoproteins that modulate transcription in a promoter context-dependent manner |  | Transcription |
| TMPO/S159/0.68 | **Thymopoietin.** The gene encodes several distinct LEM domain containing isoforms. LEM domain proteins include inner nuclear membrane and intranuclear proteins that are involved in gene expression (HDAC3 interaction), chromatin organization, and replication and cell cycle control |  | Transcription,  chromatin,  cell cycle |
| TOX4/S152/0.57 | **TOX high mobility group box family member 4.** The protein is a transcriptional modulator, it participates in reprogramming of cells and is important in DNA repair | [41, 42] | DNA repair,  transcription,  reprogramming |
| TP53BP1/S523/0.88 | **Tumor protein p53 binding protein 1.** This protein functions in the DNA double-strand break repair pathway choice, promoting non-homologous end joining (NHEJ) pathways. It plays multiple roles in the DNA damage response, including promoting checkpoint signaling following DNA damage, acting as a scaffold for recruitment of DNA damage response proteins to damaged chromatin, and promoting NHEJ pathways | [43-47] | DNA damage,  chromatin |
| TP53BP1/S525/0.88 |  |  |  |
| WDR44/S27/0.90 | **WD repeat domain 44.** The protein that interacts with the small GTPase rab11 and may play a role in endosome recycling |  | Endosomal trafficking |
| ZMYND8/S515/0.63 | **Zinc finger MYND-type containing 8.** This is a receptor for activated C-kinase (RACK) protein and binds to activated protein kinase C beta I. It contains a bromodomain and two zinc fingers and is thought to be a transcriptional regulator |  | Transcription |
