# Supplementary material for: Biological characteristics of aging in human acute myeloid leukemia cells: the possible importance of aldehyde dehydrogenase, the cytoskeleton and altered transcriptional regulation
Source: Aging (Albany NY). 2020 Dec 20;12(24):24734–77. doi: 10.18632/aging.202361 (PMC7803495; doi:10.18632/aging.202361)
Supplement: Supplementary Table 6 [file aging-12-202361-s005.docx]

**Supplementary Table 6.** **Proteins with significantly lower phosphorylation in elderly low-risk patients**

The proteins are listed in alphabetical order according to the name of the encoding gene. Phosphoproteins that are important in hematopoietic stem cells are highlighted in yellow. The table is based on information from the Gene database and selected references are from PubMed

(FC stands for phosphorylation fold change)

| **Protein/phosphorylation site(s)/FC elderly low-risk *vs* younger low-risk** | **Comment** | **Reference(s)** | **Keywords** |
| --- | --- | --- | --- |
| AATF/S320/-1.66 | **Apoptosis antagonizing transcription factor.** The protein was identified on the basis of its interaction with the protein kinase MAP3K12/DLK, a kinase involved in regulation of apoptosis. It contains a leucine zipper, that is a characteristic motif of transcription factors. AATF is a central regulator of the cellular outcome upon p53 activation, a finding that has primarily been attributed to its function as a transcription factor. It is also important for ribosome biogenesis and plays a role in rRNA maturation. The protein interactome of AATF contains a large set of proteins with a role in rRNA maturation with an emphasis on the protein-RNA-complexes required for the generation of the small ribosomal subunit | [48] | p53,  apoptosis,  transcription,  RNA,  ribosome |
| AATF/S321/-1.25 |  |  |  |
| ATAD2/S342/-1.89 | **ATPase family AAA domain containing 2.** A large family of ATPases has been described, whose key feature is that they share a conserved region of about 220 amino acids that contains an ATP-binding site. The proteins that belong to this family either contain one or two AAA (ATPases Associated with diverse cellular Activities) domains. AAA family proteins often perform chaperone-like functions that assist in the assembly, operation, or disassembly of protein complexes. The protein encoded by this gene contains two AAA domains, as well as a bromodomain. ATAD2 is recruited to replication sites through a direct interaction with di-acetylated histone H4 at K5 and K12. Atad2 is an abundant nucleosome-bound protein present on active genes, associated with chromatin remodelling, DNA replication, and DNA repair factors. A structural analysis of its bromodomain and subsequent investigations demonstrate that histone acetylation guides ATAD2 to chromatin | [49, 50] | Chromatin,  nucleosome,  histonacetylation,  chaperone |
| BCLAF1/S181/-0.74 | **BCL2 associated transcription factor 1.** This transcriptional repressor interacts with several members of the BCL2 family of proteins and overexpression of this protein induces apoptosis, which can be suppressed by co-expression of BCL2 proteins. It is also an important mediator in DNA damage-induced senescence | [51] | Transcription,  proapoptotic,  senescence |
| CDK1/T14/-1.01 | **Cyclin dependent kinase 1.** The encoded protein is a member of the Ser/Thr protein kinase family. It is a catalytic subunit of the highly conserved protein kinase complex known as M-phase promoting factor (MPF), which is essential for G1/S and G2/M phase transitions of eukaryotic cell cycle. Mitotic cyclins stably associate with this protein and function as regulatory subunits |  | Mitosis,  cell cycle |
| CDK1/Y15/-1.01 |  |  |  |
| CDK2;CDK3/T14/  -1.01 | **Cyclin dependent kinase 2.** This protein is a member of a family of serine/threonine protein kinases that participate in cell cycle regulation. It is the catalytic subunit of the cyclin-dependent protein kinase complex. Activity of this protein is especially critical during the G1 to S phase transition. Reactive oxygen species (ROS) accelerate cellular senescence. Excessive ROS generation seems to promote cellular senescence by inducing cell cycle arrest through cyclin-dependent kinase 2  **Cyclin dependent kinase 3.** This protein promotes entry into S phase, in part by activating members of the E2F family of transcription factors. The protein also associates with cyclin C and phosphorylates the retinoblastoma 1 protein to promote exit from G0 | [52] | Mitosis,  cell cycle,  senescence,  ROS |
| CDK2;CDK3/Y15  -1.01 |  |  |  |
| CHAF1A/S772/-1.67 | **Chromatin assembly factor 1 subunit A.** The chromatin assembly factor I (CAF1) is a nuclear complex consisting of p50, p60 (CHAF1B; MIM 601245), and p150 (CHAF1A) subunits that assembles histone octamers onto replicating DNA. It is regarded as a histone chaperone and an epigenetic regulator |  | Epigenetic,  histone,  transcription |
| CHD4/S1531/-0.71 | **Chromodomain helicase DNA binding protein 4.** The protein belongs to the SNF2/RAD54 helicase family, is the main component of the nucleosome remodeling and deacetylase complex and has an important role in epigenetic transcriptional repression. It seems to have a role in leukemogenesis and regulation of chemosensitivity in AML | [53, 54] | Helicase  nucleosome,  transcription,  epigenetic |
| CHD9/S616/-0.72 | **Chromodomain helicase DNA binding protein 9.** This nucleolar protein is involved in the regulation of the chromatin structure | [55] | Nucleolus,  chromatin,  transcription |
| CNP/S150/-0.97 | **2',3'-cyclic nucleotide 3' phosphodiesterase.** The activity of X box-binding protein 1 (XBP1), a master transcriptional regulator of endoplasmic reticulum homeostasis and the unfolded protein response, is controlled by a two-step splicing reaction in the cytoplasm. CNP suppresses the second step in *XBP1* splicing | [56] | Transcription,  splicing |
| EEF2/T57/-0.86 | **Eukaryotic translation elongation factor 2.** This gene encodes a member of the GTP-binding translation elongation factor family. This protein is an essential factor for protein synthesis. It promotes the GTP-dependent translocation of the nascent protein chain from the A-site to the P-site of the ribosome | [57] | Protein synthesis |
| EEF2/T59/-0.86 |  |  |  |
| EIF3F/S258/-0.76 | **Eukaryotic translation initiation factor 3 subunit F.** The protein has tumor suppressor activity, is an inhibitor of translation and regulates RNA degradation | [58] | Tumor suppressor,  translation,  RNA |
| ESCO2/S75/-1.73 | **Establishment of sister chromatid cohesion N-acetyltransferase 2.** This protein may have acetyltransferase activity and be required for the establishment of sister chromatid cohesion during the S phase of mitosis |  | Acetyltransferase,  transcription,  mitosis |
| ESYT2/S727/-0.70 | **Extended synaptotagmin 2.** The protein is probably important for the control of lipid transfer at membrane contact sites, i.e. between organelles | [59] | Lipid transport |
| FAM83H/S892/-1.08 | **Family with sequence similarity 83 member H.** The protein encoded by this gene plays an important role in the structural development and calcification of tooth enamel. The long non-coding RNA FAM83H-AS1 RNA seems to have oncogenic functions by suppressing expression of the cell cycle regulator CDKN1A | [60] | Cell cycle? |
| FOXK2/S373/-0.67 | **Forkhead box K2.** This protein contains a fork head DNA binding domain. It may be involved in the regulation of cellular promoter elements. FoxK2 is an important regulator of cellular metabolism and mitochondrial functions, is a regulator of proliferation and survival, promotes Wnt signaling, functions as a transcriptional regulator (interacting with AT-1), expression is increased during mitosis and phosphorylation is also increased during mitosis by CDK1. Its prognostic impact in cancer seems to depend on the biological context | [61-63] | Transcription,  AT1,  mitosis,  CDK1,  survival,  mitochondria  metabolism |
| GSG2/S147/-0.91 | **Germ cell associated 2, haspin.** The protein regulates spindle assembly, affects the phosphorylation state of proteins involved in gene expression regulation and splicing and protects centromeric cohesion in mitosis | [64-66] | Mitosis,  transcription,  splicing |
| GTSE1/S575/-1.23 | **G2 and S-phase expressed 1.** The protein encoded by this gene is only expressed in the S and G2 phases of the cell cycle, where it colocalizes with cytoplasmic tubulin and microtubules. In response to DNA damage, the encoded protein accumulates in the nucleus and binds the tumor suppressor protein p53, shuttling it out of the nucleus and repressing its ability to induce apoptosis | [67] | DNA damage, cell cycle |
| GTSE1/S580/-1.23 |  |  |  |
| HIST1H1D/T18/-1.79 | **H1.3 linker histone, cluster member.** Two molecules of each of the four core histones (H2A, H2B, H3, and H4) form an octamer, around which approximately 146 bp of DNA is wrapped in repeating units, called nucleosomes. The linker histone, H1, interacts with linker DNA between nucleosomes and functions in the compaction of chromatin into higher order structures. This gene encodes a replication-dependent member of the histone H1 family |  | Histone |
| HSP90AB1/S226/-1.26 | **Heat shock protein 90 alpha family class B member 1.** The protein is a member of the heat shock protein 90 family; these proteins are involved in signal transduction, protein folding and degradation and morphological evolution |  | HSP90,  chaperon |
| IGF2BP1/S181/-1.29 | **Insulin like growth factor 2 mRNA binding protein 1.** This member of the insulin-like growth factor 2 mRNA-binding protein family that functions by binding to the mRNAs of certain genes, including insulin-like growth factor 2 (IGF2), beta-actin and beta-transducin repeat-containing protein, and regulating their translation |  | RNA,  actin,  IGF2 |
| INCENP/S214/-1.16 | **Inner centromere protein.** The inner centromere proteins display a broad localization along chromosomes in the early stages of mitosis but gradually become concentrated at centromeres as the cell cycle progresses into mid-metaphase. During telophase, the proteins are located within the midbody in the intercellular bridge, where they are discarded after cytokinesis |  | Mitosis,  centromer |
| ING5/S118/-0.96 | **Inhibitor of growth family member 5.** This tumor suppressor protein inhibits cell growth and induces apoptosis. This protein contains a PHD-type zinc finger and it interacts with p53 and p300, a component of the histone acetyl transferase complex, suggesting a role in transcriptional regulation. It stabilizes p53 | [68] | Tumor suppressor  p53,  histone,  transcription |
| IRS2/S608/-1.03 | **Insulin receptor substrate 2.** This cytoplasmic signaling molecule that mediates effects of insulin, insulin-like growth factor 1, and other cytokines by acting as a molecular adaptor between diverse receptor tyrosine kinases and downstream effectors |  | Intracellular signaling |
| KMT2A/S2060/-0.78 | **Lysine methyltransferase 2A.** This transcriptional coactivator plays an essential role in regulating gene expression during early development and hematopoiesis. Its SET domain, is responsible for its histone H3 lysine 4 (H3K4) methyltransferase activity and mediates chromatin modifications associated with epigenetic transcriptional activation. The protein regulates the transcription of specific target genes, including many of the HOX genes, and is important for hematopoietic stem and progenitor cell homeostasis | [69] | Transcription,  hematopoiesis,  Hox genes,  hematopoietic stem cells |
| LIG1/S36/-0.67 | **DNA ligase 1.** This protein is a member of the ATP-dependent DNA ligase protein family. It functions in DNA replication, recombination, and the base excision repair process |  | DNA repair |
| LIG1/S46/-0.67 |  |  |  |
| NPM1/S70/-0.88 | **Nucleophosmin 1.** The protein is involved in several cellular processes, including centrosome duplication, protein chaperoning, and cell proliferation. It shuttles between the nucleolus, nucleus, and cytoplasm, chaperoning ribosomal proteins and core histones from the nucleus to the cytoplasm |  | Centrosome,  protein chaperoning,  ribosome,  histone |
| PCYT1B/S315/-1.01 | **Phosphate cytidylyltransferase 1, choline, beta.** The protein belongs to the cytidylyltransferase family. It is involved in the regulation of phosphatidylcholine biosynthesis. It probably contributes to lipid metabolism and phospholipid compositional homeostasis | [70] | Metabolism |
| POLA2/S152/-1.11 | **DNA polymerase alpha 2, accessory subunit.** The CST (CTC1-STN1-TEN1) complex mediates critical functions in maintaining telomere DNA and overcoming genome-wide replication stress. A conserved biochemical function of the CST complex is its primase-Pol α stimulatory activity. The STN1-POLA2 interaction seems to promote the conformational change for nucleic acid delivery to Pol α and subsequent DNA synthesis, and these activities are necessary for the in vivo function of STN1 | [71] | Orotein conformation,  telomere |
| POLA2/S141/-0.96 |  |  |  |
| PPIG/S546/-0.71 | **Peptidylprolyl isomerase G.** The protein is regarded as a splicing factor | [72] | Splicing factor |
| PPP6R3/S523/-0.70 | **Protein phosphatase 6 regulatory subunit 3.** Protein phosphatase regulatory subunits, such as SAPS3, modulate the activity of protein phosphatase catalytic subunits by restricting substrate specificity, recruiting substrates, and determining the intracellular localization of the holoenzyme. SAPS3 is a regulatory subunit for the protein phosphatase-6 catalytic subunit |  | Protein phosphorylation |
| PPP6R3/S524/-0.68 |  |  |  |
| PRPF40A/S935/-0.73 | **Pre-mRNA processing factor 40 homolog A.** This protein functions as a splicing factor | [73] | Splicing |
| RB1/T826/-1.18 | **RB transcriptional corepressor 1.** The protein a negative regulator of the cell cycle and a tumor suppressor. It also stabilizes constitutive heterochromatin. The active, hypophosphorylated form of the protein binds transcription factor E2F1 |  | Cell cycle,  tumor suppressor,  transcription,  chromatin |
| RB1/T821/-1.12 |  |  |  |
| RB1/T373/-0.82 |  |  |  |
| RB1/S807/-0.74 |  |  |  |
| RB1/S811/-0.74 |  |  |  |
| RPRD2/S374/-0.80 | **Regulation of nuclear pre-mRNA domain containing 2.** Among its related pathways are Gene Expression and Formation of HIV-1 elongation complex containing HIV-1 Tat. Diseases associated with RPRD2 include Retinitis Pigmentosa 30 | [74] | RNA |
| RRP1B/S513/-1.32 | **Ribosomal RNA processing 1B.** The protein is associated with metastasis in several solid tumors. This is a nuclear protein that interacts with bromodomains and is a chromatin-associated protein. One of its targets is SRSF1 (serine/arginine-rich splicing factor 1) (SF2/ASF, splicing factor 2/alternative splicing factor), an essential splicing regulator that also functions as an oncoprotein | [75-77] | Transcription,  chromatin,  splicing |
| RRP36/S67/-1.29 | **Ribosomal RNA processing 36.** RRP36 functions at an early stage in the processing of 35S preribosomal RNA into the mature 18S species. Ribosome biogenesis in eukaryotes is a major cellular activity mobilizing the products of over 200 transcriptionally coregulated genes referred to as the rRNA and ribosome biosynthesis regulon. The Rrp36p protein is nucleolar and interacts with 90S and pre-40S preribosomal particles. Its depletion affects early cleavages of the 35S pre-rRNA and results in a rapid decrease in mature 18S rRNA levels. Rrp36p is a novel component of the 90S preribosome | [78] | RNA,  ribosome |
| SCRIB/S1306/-0.70 | **Scribble planar cell polarity protein.** This scaffold protein is involved in tumor suppression pathways. It is important for cell polarization through its binding to many other proteins. It is important for the self-renewal capacity of hematopoietic stem cells and Scrib-deficient hematopoietic stem cells are functionally impaired | [79] | Scaffold protein,  tumor suppressor,  hematopoietic stem cells |
| SRRM1/S402/-1.20 | **Serine and arginine repetitive matrix 1.** Chimeric RNAs generated by cis-splicing between adjacent genes (cis-SAGe) are increasingly recognized as a widespread phenomenon. The two factors SRRM1 and SF3B1 affect both cis-SAGe chimeras and other types of chimeric RNAs in a genome-wide fashion | [80] | RNA splicing |
| SRRM1/S431/-0.74 |  |  |  |
| SRRM1/S775/-0.70 |  |  |  |
| SRRM2/S1552/-0.91 | **Serine/arginine repetitive matrix 2.** The protein is another mRNA splicing factor | [81] | RNA splicing |
| SRRM2/S2046/-0.75 |  |  |  |
| SRRM2/S2690/-0.74 |  |  |  |
| SRRM2/S2692/-0.74 |  |  |  |
| SRRM2/S2694/-0.74 |  |  |  |
| STT3B/S499/-0.65 | **STT3 oligosaccharyltransferase complex catalytic subunit B.** The protein is a catalytic subunit of a protein complex that transfers oligosaccharides onto asparagine residues |  | Protein glycosylation |
| YBX1/S174/-0.87 | **Y-box binding protein 1.** This cold shock domain protein is both a DNA and RNA binding protein and has been implicated in regulation of transcription and translation, pre-mRNA splicing, DNA repair and mRNA packaging. It is also a component of messenger ribonucleoprotein (mRNP) complexes and may have a role in microRNA processing. This protein can also be secreted through non-classical pathways and functions as an extracellular mitogen. It enhances the proliferation of human AML cells | [82] | DNA repair,  RNA,  transcription, splicing,  AML |
| TOR1AIP/S156/-1.05 | **Torsin 1A interacting protein 1.** This type 2 integral membrane protein binds A- and B-type lamins. The encoded protein localizes to the inner nuclear membrane and may be involved in maintaining the attachment of the nuclear membrane to the nuclear lamina during cell division |  | Nucleus,  mitosis |
| ZC3H13/S1017/-0.75 | **Zinc finger CCCH-type containing 13.** The nuclear protein is important for RNA methylation and it is also important for stem cell self-renewal | [83, 84] | RNA,  stem cell self-renewal |
