# Supplementary material for: Biological characteristics of aging in human acute myeloid leukemia cells: the possible importance of aldehyde dehydrogenase, the cytoskeleton and altered transcriptional regulation
Source: Aging (Albany NY). 2020 Dec 20;12(24):24734–77. doi: 10.18632/aging.202361 (PMC7803495; doi:10.18632/aging.202361)
Supplement: Supplementary Table 7 [file aging-12-202361-s006.docx]

**Supplementary Table 7.** **Proteins with significantly different expression and phosphorylation levels in high-risk and low-risk patients**

A summary of the characteristics for proteins included in the interacting protein networks presented in Figures 4-5. The table is based on information from the Gene database. Gene descriptions of the neutrophil degranulation and platelet degranulation Reactome pathways in Figure 4 contain additional information on tissue gene expression

| **ID.** | **Comment** | **KeywordS** |
| --- | --- | --- |
| **Figure 4B: Neutrophil degranulation** | | |
| CFD | **Complement factor D***.* This protein is a member of the S1, or chymotrypsin, family of serine peptidases. Alternative splicing of this gene results in multiple transcripts. At least one of these variants encodes a preproprotein that is proteolytically processed to generate the mature protease. The gene shows a high expression in fat tissues | Complement activation,  protease |
| STK11IP | **Serine/threonine kinase 11 interacting protein.** The gene shows a broad expression in many tissues including bone marrow, spleen and lymph nodes | Kinase |
| ARSA | **Arylsulfatase A.** The protein hydrolyzes cerebroside sulfate to cerebroside and sulfate. The gene shows a broad expression in many different tissues including bone marrow, spleen and lymph nodes | Cerebroside sulfatation |
| AGA | **Aspartylglucosaminidase***.* This member of the N-terminal nucleophile (Ntn) hydrolase family is proteolytically processed to generate alpha and beta chains that comprise the mature enzyme. This enzyme is involved in the catabolism of N-linked oligosaccharides of glycoproteins. It cleaves asparagine from N-acetylglucosamines as one of the final steps in the lysosomal breakdown of glycoproteins. The gene shows a broad expression in many different tissues including bone marrow, spleen and lymph nodes | Lysosome,  glycoprotein breakdown |
| MPO | **Myeloperoxidase***.* Myeloperoxidase is synthesized during myeloid differentiation that constitutes the major component of neutrophil azurophilic granules. Produced as a single chain precursor, myeloperoxidase is subsequently cleaved into a light and heavy chain. Restricted gene expression toward bone marrow | Neutrophils, |
| ARSB | **Arylsufatase B**. The protein homodimer hydrolyzes sulfate groups of N-Acetyl-D-galactosamine, chondroitin sulfate, and dermatan sulfate. The protein is targeted to the lysozyme. The gene shows a broad expression in many tissues including bone marrow, spleen and lymph nodes | Lysosome sulfate hydrolysis |
| GUSB | **Glucuronidase beta***.* This gene encodes a hydrolase that degrades glycosaminoglycans, including heparan sulfate, dermatan sulfate, and chondroitin-4,6-sulfate. The enzyme forms a homotetramer that is localized to the lysosome. The gene shows a broad expression in many tissues including bone marrow, spleen and lymph nodes | Lysosome,  hydrolase |
| DOCK2 | **Dedicator of cytokinesis 2***.* The protein is involved in remodeling of the actin cytoskeleton required for migration in response to chemokine signaling. It activates members of the Rho family of GTPases, for example RAC1 and RAC2, by acting as a guanine nucleotide exchange factor (GEF) to exchange bound GDP for free GTP. The gene shows a broad expression in many tissues including bone marrow, spleen and lymph nodes | Actin, cytoskeleton,  RAC1, RAC2 |
| VCL | **Vinculin***.* Vinculin is a cytoskeletal protein associated with cell-cell and cell-matrix junctions, where it is thought to function as one of several interacting proteins involved in anchoring F-actin to the membrane. Broad expression in many organs including endometrium and prostate | F-actin,  cytoskeleton |
| CFP | **Complement factor properdin***.* This plasma glycoprotein regulates positively the alternative complement pathway of the innate immune system. The gene shows a broad expression in many tissues including bone marrow, spleen and lymph nodes | Complement |
| METTL7A | **Methyltransferase like 7A.** The gene shows a broad expression in many tissues including liver and thyroid | Methyltransferase |
| ASAH1 | **N-acylsphingosine amidohydrolase 1***.* This is an acid ceramidase. Alternative splicing results in multiple transcript variants, at least one of which encodes a preproprotein that is proteolytically processed. Processing of this preproprotein generates alpha and beta subunits that heterodimerize to form the mature lysosomal enzyme, which catalyzes the degradation of ceramide into sphingosine and free fatty acid. This enzyme is overexpressed in multiple human cancers and may play a role in cancer progression | Lysosome,  ceramide degradation,  lipid metabolism |
| CST3 | **Cystatin C***.* This gene is located in the cystatin locus and encodes the most abundant extracellular inhibitor of cysteine proteases, which is found in high concentrations in biological fluids and is expressed in virtually all organs of the body. The gene shows a broad expression in many tissues including bone marrow, spleen and lymph nodes | Protease inhibitor |
| NBEAL2 | **Neurobeachin like 2***.* The protein contains a beige and Chediak-Higashi (BEACH) domain and multiple WD40 domains, and may play a role in megakaryocyte alpha-granule biogenesis. High expression in bone marrow, spleen and lymph nodes as well as upper gastrointestinal tract | Megakaryocyte  granule biogenesis |
| CD59 | **CD59***.* This cell surface glycoprotein regulates complement-mediated cell lysis and is involved in signal transduction. This protein is a potent inhibitor of the complement membrane attack complex. The gene shows a broad expression in many tissues including bone marrow, spleen and lymph nodes | Cell surface,  complement,  signal transduction |
| ADAM10 | **ADAM metallopeptidase domain 10***.* Members of the ADAM family are cell surface proteins with a unique structure possessing both potential adhesion and protease domains. This ADAM family member cleaves many proteins including TNF-alpha and E-cadherin. The gene shows a broad expression in many different tissues including bone marrow, spleen and lymph nodes | Cell surface,  protease,  adhesion |
| RAB37 | **RAB37, member RAS oncogene family***.* Rab proteins are low molecular mass GTPases that are critical regulators of vesicle trafficking. The gene shows a broad expression in many tissues including bone marrow, spleen and lymph nodes | GTPase,  Vesicle trafficking |
| CPNE3 | **Copine 3***.* Calcium-dependent membrane-binding proteins may regulate molecular events at the interface of the cell membrane and cytoplasm. This gene encodes a protein which contains two type II C2 domains in the amino-terminus and an A domain-like sequence in the carboxy-terminus. The A domain mediates interactions between integrins and extracellular ligands. The gene shows a broad expression in many tissues including bone marrow, spleen and lymph nodes | Cell membrane,  integrins |
| LAMP1/2 | **Lysosomal associated membrane protein 1/2***.* The proteins encoded by these genes are member of a family of membrane glycoproteins. These glycoproteins provide selectins with carbohydrate ligands. They may also play a role in tumor cell metastasis. The genes show a broad expression in many tissues including bone marrow, spleen and lymph nodes | Lysosome |
| MGST1 | **Microsomal glutathione S-transferase 1***.* This protein catalyzes the conjugation of glutathione to electrophiles and the reduction of lipid hydroperoxides. This protein is localized to the endoplasmic reticulum and outer mitochondrial membrane where it is thought to protect these membranes from oxidative stress. Relatively low expression in bone marrow, high expression especially in fat, liver and adrenals | Endoplasmic reticulum,  mitochondria,  oxidative stress |
| ALDH3B1 | **Aldehyde dehydrogenase 3 family member B1.** This member of the aldehyde dehydrogenase protein family is important for lipid peroxidation. It is able to oxidize long-chain fatty aldehydes, and may play a role in protection from oxidative stress. Broad gene expression in lung and bone marrow | Aldehyde dehydrogenase |
| TSPAN14 | **Tetraspanin 14.** Most tetraspanins are cell-surface proteins that are characterized by the presence of four hydrophobic domains. The proteins mediate signal transduction. The gene shows a broad expression in many tissues including bone marrow, spleen and lymph nodes | Signal transduction |
| CLEC12A | **C-type lectin domain family 12 member A.** This protein is a member of the C-type lectin/C-type lectin-like domain (CTL/CTLD) superfamily. Members of this family share a common protein fold and have diverse functions, such as cell adhesion, cell-cell signaling, glycoprotein turnover, and roles in inflammation and immune response. The gene shows high expression especially in spleen and lymph nodes | Cell adhesion, inflammation,  immune response |
| SLC27A2 | **Solute carrier family 27 member 2.** The protein is an isozyme of long-chain fatty-acid-coenzyme A ligase family that converts free long-chain fatty acids into fatty acyl-CoA esters, and thereby play a key role in lipid biosynthesis and fatty acid degradation. This isozyme activates long-chain, branched-chain and very-long-chain fatty acids containing 22 or more carbons to their CoA derivatives. It is expressed primarily in liver and kidney, and is present in both endoplasmic reticulum and peroxisomes, but not in mitochondria | Lipid metabolism,  endoplasmic reticulum,  peroxisomes |
| VPS35L | **VPS35 endosomal protein sorting factor like.** Broad expression in many organs including bone marrow, lymph nodes and spleen | Endosome |
| **Figure 4B: platelet degranulation** | | |
| CFD | **Complement factor D.** This protein is a member of the S1, or chymotrypsin, family of serine peptidases. Alternative splicing of this gene results in multiple transcripts. At least one of these variants encodes a preproprotein that is proteolytically processed to generate the mature protease. The gene shows a high expression in fat tissues | Complement activation,  protease |
| VWF | **Von Willebrand factor***.* This glycoprotein is involved in hemostasis. It is proteolytically processed into large multimeric complexes that function in the adhesion of platelets to sites of vascular injury and the transport of various proteins in the blood. It is expressed in various organs and the expression in normal bone marrow is low | Coagulation |
| SCCPDH | **Saccharopine dehydrogenase** (putative). The protein may be involved in Axl mediated signaling.The gene shows a broad expression in many tissues including bone marrow, spleen and lymph nodes | Signal transduction |
| ACTN1 | **Actinin alpha 1***.* Alpha actinins belong to the spectrin gene superfamily, which represents a diverse group of cytoskeletal proteins. Alpha actinin is an actin-binding protein with multiple roles in different cell types. In nonmuscle cells, the cytoskeletal isoform is found along microfilament bundles and adherent-type junctions, where it is involved in binding actin to the membrane. This gene encodes a nonmuscle, cytoskeletal, alpha actinin isoform. The gene shows a broad expression in many tissues including bone marrow, spleen and lymph nodes | Cytoskeleton |
| VCL | **Vinculin***.* Vinculin is a cytoskeletal protein associated with cell-cell and cell-matrix junctions, where it is thought to function as one of several interacting proteins involved in anchoring F-actin to the membrane. Broad expression in many organs including endometrium and prostate | F-actin,  cytoskeleton |
| LAMP2 | **Lysosomal associated membrane protein 2***.* The protein encoded by this gene is a member of a family of membrane glycoproteins. This glycoprotein provides selectins with carbohydrate ligands. It may also play a role in tumor cell metastasis. The gene shows a broad expression in many tissues including bone marrow, spleen and lymph nodes | Lysosome |
| TUBA4A | **Tubulin alpha 4a.** Microtubules of the eukaryotic cytoskeleton perform essential and diverse functions and are composed of a heterodimer of alpha and beta tubulin. These microtubule constituents are part of the tubulin superfamily, which is composed of six distinct families. Genes from the alpha, beta and gamma tubulin families are found in all eukaryotes. This molecule may be involved in carcinogenesis and/or chemoresistance. The gene shows a broad expression in many tissues including bone marrow, spleen and lymph nodes | Microtubuli |
| RAB27B | **RAB27B, member RAS oncogene family***.* Members of the Rab protein family, including RAB27B, are prenylated, membrane-bound proteins involved in vesicular fusion and trafficking. Biased gene expression in stomach and prostate | Vesicular trafficking |
| **Figure 4B: POST-TRANSLATIONAL PROTEIN MODIFICATION** | | |
| EDEM3 | **ER degradation enhancing alpha-mannosidase like protein 3.** EDEM3 belongs to a group of proteins that accelerate degradation of misfolded glycoproteins in the ER | Mannose trimming |
| MOGS | **Mannosyl-oligosaccharide glucosidase.** This is the first enzyme in the N-linked oligosaccharide processing pathway. The enzyme cleaves the distal alpha-1,2-linked glucose residue from the Glc(3)-Man(9)-GlcNAc(2) oligosaccharide precursor | Glucosidase activity |
| RCN1 | **Reticulocalbin 1.** Reticulocalbin 1 is a calcium-binding protein located in the lumen of the ER. The protein contains six conserved regions with similarity to a high affinity Ca(+2)-binding motif, the EF-hand | Calcium ion binding |
| CALR | **Calreticulin.** Calreticulin is a highly conserved chaperone protein which resides primarily in the endoplasmic reticulum and is involved in a variety of cellular processes, among them, cell adhesion. Additionally, it functions in protein folding quality control and calcium homeostasis | Cell adhesion,  protein folding |
| COPG2 | **COPI coat complex subunit gamma 2** | Intracellular protein transport |
| CD59 | **CD59***.* This gene encodes a cell surface glycoprotein that regulates complement-mediated cell lysis. This protein also plays a role in signal transduction pathways | Complement,  signal transduction |
| CFP | **Complement factor properdin***.* This plasma glycoprotein regulates positively the alternative complement pathway of the innate immune system | Complement |
| CST3 | **Cystatin C***.* The cystatin superfamily encompasses proteins that contain multiple cystatin-like sequences. Some of the members are active cysteine protease inhibitors, while others have lost or perhaps never acquired this inhibitory activity. There are three inhibitory families in the superfamily, including the type 1 cystatins (stefins), type 2 cystatins and the kininogens. The type 2 cystatin proteins are a class of cysteine proteinase inhibitors found in a variety of human fluids and secretions, where they appear to provide protective functions. The cystatin locus on chromosome 20 contains the majority of the type 2 cystatin genes and pseudogenes. This gene is located in the cystatin locus and encodes the most abundant extracellular inhibitor of cysteine proteases | Protease inhibitor |
| RAB37 | **RAB37, member RAS oncogene family***.* Rab proteins are low molecular mass GTPases that are critical regulators of vesicle trafficking | Transport |
| ADAM10 | **ADAM metallopeptidase domain 10***.* Members of the ADAM family are cell surface proteins with a unique structure possessing both potential adhesion and protease domains. This gene encodes an ADAM family member that cleaves many proteins including TNF-alpha and E-cadherin | Protease |
| RAB27B | **RAB27B, member RAS oncogene family***.* Members of the Rab protein family, including RAB27B, are prenylated, membrane-bound proteins involved in vesicular fusion and trafficking | Vesicular trafficking |
| VDAC1 | **Voltage dependent anion channel 1.** This gene encodes a voltage-dependent anion channel protein that is a major component of the outer mitochondrial membrane. The encoded protein facilitates the exchange of metabolites and ions across the outer mitochondrial membrane and may regulate mitochondrial functions | Anion transmembrane transport |
| ARSA | **Arylsulfatase A.** This protein hydrolyzes cerebroside sulfate to cerebroside and sulfate. Defects in this gene lead to metachromatic leucodystrophy (MLD), a progressive demyelination disease which results in a variety of neurological symptoms and ultimately death | Glycosphingolipid metabolism |
| ARSB | **Arylsufatase B**. Arylsulfatase B belongs to the sulfatase family. The arylsulfatase B homodimer hydrolyzes sulfate groups of N-acetyl-D-galactosamine, chondriotin sulfate and dermatan sulfate. The protein is targeted to the lysozyme | Sulfatase,  lysozyme |
| SUMF2 | **Sulfatase modifying factor 2.** This protein is a member of the sulfatase-modifying factor family and has a DUF323 domain that localizes to the lumen of the endoplasmic reticulum. This protein has low levels of C-alpha formylglycine (FGly)-generating activity but can heterodimerize with another family member - a protein with high levels of FGly-generating activity | Endoplasmic reticulum |
| ACTR8 | **Actin related protein 8** | Cell division |
| XPC | **XPC complex subunit, DNA damage recognition and repair factor.** The protein is a key component of the XPC complex, which plays an important role in the early steps of global genome nucleotide excision repair (NER). The encoded protein is important for damage sensing and DNA binding, and shows a preference for single-stranded DNA | Damage DNA binding |
| RAD23B | **RAD23 homolog B, nucleotide excision repair protein.** The protein is one of two human homologs of Saccharomyces cerevisiae Rad23, a protein involved in the nucleotide excision repair (NER). This protein was found to be a component of the protein complex that specifically complements the NER defect of xeroderma pigmentosum group C (XP-c) cell extracts in vitro. This protein was also shown to interact with, and elevate the nucleotide excision activity of 3-methyladenine-DNA glycosylase (MPG), which suggested a role in DNA damage recognition in base excision repair | Damage DNA binding |
| TUBA4A | **Tubulin alpha 4a.** Microtubules of the eukaryotic cytoskeleton perform essential and diverse functions and are composed of a heterodimer of alpha and beta tubulin. These microtubule constituents are part of the tubulin superfamily, which is composed of six distinct families. Genes from the alpha, beta and gamma tubulin families are found in all eukaryotes | Cytoskeleton |
| TUBA1B | **Tubulin alpha 1b** | Cytoskeleton |
| TUBA1C | **Tubulin alpha 1c** | Cytoskeleton |
| TUBB2B | **Tubulin beta 2B class IIb.** The protein is a beta isoform of tubulin, which binds GTP and is a major component of microtubules | Cytoskeleton |
| **Figure 4B: COPI-dependent Golgi-to-ER retrograde traffic** | | |
| NBAS | **NBAS subunit of NRZ tethering complex.** This protein has two leucine zipper domains, a ribosomal protein S14 signature domain and a Sec39 like domain. The protein is thought to be involved in Golgi-to-ER transport | Intracellular transport |
| STX18 | **Syntaxin-18.** The protein belongs to the syntaxin family of soluble N-ethylmaleimide-sensitive factor attachment protein receptors (SNAREs) which is part of a membrane tethering complex that includes other SNAREs and several peripheral membrane proteins, and is involved in vesicular transport between the endoplasmic reticulum and the Golgi complex | Exocytosis |
| COPG2 | **COPI coat complex subunit gamma 2** | Intracellular protein transport |
| TUBA4A | **Tubulin alpha 4a.** Microtubules of the eukaryotic cytoskeleton perform essential and diverse functions and are composed of a heterodimer of alpha and beta tubulin. These microtubule constituents are part of the tubulin superfamily, which is composed of six distinct families. Genes from the alpha, beta and gamma tubulin families are found in all eukaryotes | Cytoskeleton |
| TUBA1B | **Tubulin alpha 1b** | Cytoskeleton |
| TUBA1C | **Tubulin alpha 1c** | Cytoskeleton |
| TUBB2B | **Tubulin beta 2B class IIb.** The protein is a beta isoform of tubulin, which binds GTP and is a major component of microtubules | Cytoskeleton |
| **Figure 4B: CELL-Extracellular matrix interactions** | | |
| ACTN1 | **Actinin alpha 1***.* Alpha actinins belong to the spectrin gene superfamily which represents a diverse group of cytoskeletal proteins, including the alpha and beta spectrins and dystrophins. Alpha actinin is an actin-binding protein with multiple roles in different cell types. It can be found along microfilament bundles and adherens-type junctions and be involved in binding actin to the membrane. This protein is a nonmuscle, cytoskeletal, alpha actinin | Cytoskeleton |
| PARVB | **Parvin beta.** This protein is a member of the parvin family of actin-binding proteins, which play a role in cytoskeleton organization and cell adhesion. These proteins are associated with focal contacts and contain calponin homology domains that bind to actin filaments. This family member binds to alphaPIX and alpha-actinin, and it can inhibit the activity of integrin-linked kinase. This protein also functions in tumor suppression | Cytoskeleton,  adhesion,  tumor suppressor |
| ILK | **Integrin linked kinase***.* This protein has a kinase-like domain and four ankyrin-like repeats. The encoded protein associates at the cell membrane with the cytoplasmic domain of beta integrins, where it regulates integrin-mediated signal transduction | Adhesion,  integrin |
| RSU1 | **Ras suppressor protein 1.** This protein is involved in the Ras signal transduction pathway and growth inhibition. In mouse, the encoded protein was initially isolated based on its ability to inhibit v-Ras transformation | Ras signaling |
| **Figure 5B: Apoptotic execution PHASE** | | |
| SPTAN1 | **Spectrin alpha, non-erythrocytic 1***.* Spectrins are a family of filamentous cytoskeletal proteins that function as essential scaffold proteins that stabilize the plasma membrane and organize intracellular organelles. Spectrins are composed of alpha and beta dimers that associate to form tetramers linked in a head-to-head arrangement. Alpha spectrin is specifically expressed in nonerythrocytic cells and has been implicated in other cellular functions including DNA repair and cell cycle regulation | Cytoskeleton,  DNA repair,  cell cycle regulation |
| TJP2 | **Tight junction protein 2.** This protein is a member of the membrane-associated guanylate kinase homolog family. The protein functions as a component of the tight junction barrier and is necessary for proper assembly of tight junctions | Cell adhesion,  tight junction |
| STK24 | **Serine/threonine kinase 24.** This serine/threonine protein kinase that functions upstream of mitogen-activated protein kinase (MAPK) signaling. The encoded protein is cleaved into two chains by caspases; the N-terminal fragment (MST3/N) translocates to the nucleus and promotes programmed cells death | Proapoptotic kinase |
| ACIN1 | **Apoptotic chromatin condensation inducer 1.** It is a nuclear protein that induces apoptotic chromatin condensation after activation by caspase-3, without inducing DNA fragmentation. This protein has also been shown to be a component of a splicing-dependent multiprotein exon junction complex (EJC) that is deposited at splice junctions on mRNAs, as a consequence of pre-mRNA splicing | Apoptotic chromosome condensation |
| PLEC | **Plectin.** Plectin is a prominent member of an important family of structurally and in part functionally related proteins, termed plakins or cytolinkers, that are capable of interlinking different elements of the cytoskeleton | Cytoskeleton |
| DNM1L | **Dynamin 1 like.** This member of the dynamin superfamily of GTPases mediates mitochondrial and peroxisomal division and is involved in developmentally regulated apoptosis and programmed necrosis | GTPase,  apoptosis,  mitochondria |
| LMNA | **Lamin A/C.** The nuclear lamina consists of a two-dimensional matrix of proteins located next to the inner nuclear membrane. The lamin family of proteins make up the matrix. During mitosis, the lamina matrix is reversibly disassembled as the lamin proteins are phosphorylated. Lamin proteins are thought to be involved in nuclear stability, chromatin structure and gene expression | Nuclear membrane |
| LMNB1 | **Lamin B1.** This protein is one of the two B-type lamin proteins and is a component of the nuclear lamina | Nuclear membrane |
| **Figure 5B: Mitotic prophase** | | |
| CDK1 | **Cyclin dependent kinase 1***.* The Ser/Thr protein kinase is a catalytic subunit of the highly conserved protein kinase complex known as M-phase promoting factor (MPF), which is essential for G1/S and G2/M phase transitions of the cell cycle. Mitotic cyclins stably associate with this protein and function as regulatory subunits. The kinase activity of this protein is controlled by cyclin accumulation and destruction through the cell cycle. The phosphorylation and dephosphorylation of this protein also play important regulatory roles in cell cycle control | Cell cycle regulation |
| RB1 | **RB transcriptional corepressor 1***.* The protein is a negative regulator of the cell cycle and was the first tumor suppressor gene found. The encoded protein also stabilizes constitutive heterochromatin and the active, hypophosphorylated form of the protein binds transcription factor E2F1 | Tumor suppressor |
| TPR | **Translocated promoter region, nuclear basket protein***.* This large coiled-coil protein forms intranuclear filaments attached to the inner surface of nuclear pore complexes (NPCs). It is required for the nuclear export of mRNAs and some proteins | Nuclear export |
| LMNA | **Lamin A/C.** The nuclear lamina consists of a two-dimensional matrix of proteins located next to the inner nuclear membrane. The lamin family of proteins make up the matrix. During mitosis, the lamina matrix is reversibly disassembled as the lamin proteins are phosphorylated. Lamin proteins are thought to be involved in nuclear stability, chromatin structure and gene expression | Nuclear membrane |
| NUMA1 | **Nuclear mitotic apparatus protein 1***.* This large protein forms a structural component of the nuclear matrix. The encoded protein interacts with microtubules and plays a role in the formation and organization of the mitotic spindle | Nuclear matrix,  microtubule |
| **FIGURE 5C: MITOTIC CELL CYCLE PROCESS** | | |
| NUMA1 | **Nuclear mitotic apparatus protein 1***.* This large protein forms a structural component of the nuclear matrix. The encoded protein interacts with microtubules and plays a role in the formation and organization of the mitotic spindle | Nuclear matrix,  microtubule |
| CDK1 | **Cyclin dependent kinase 1***.* The Ser/Thr protein kinase is a catalytic subunit of the highly conserved protein kinase complex known as M-phase promoting factor (MPF), which is essential for G1/S and G2/M phase transitions of the cell cycle. Mitotic cyclins stably associate with this protein and function as regulatory subunits. The kinase activity of this protein is controlled by cyclin accumulation and destruction through the cell cycle. The phosphorylation and dephosphorylation of this protein also play important regulatory roles in cell cycle control | Cell cycle regulation |
| NCAPG | **Non-SMC condensin I complex subunit G.** The protein is a subunit of the condensin complex, which is responsible for the condensation and stabilization of chromosomes during mitosis and meiosis. Phosphorylation of the encoded protein activates the condensin complex | Condensin,  mitosis |
| TUBB | **Tubulin beta class I.** This protein forms a dimer with alpha tubulin and acts as a structural component of microtubules. Alternative splicing results in multiple splice variants | Microtubules |
| CEP131 | **Centrosomal protein 131** | Cell differentiation |
| STMN1 | **Stathmin 1.**.This protein is an ubiquitous cytosolic phosphoprotein proposed to function as an intracellular relay integrating regulatory signals of the cellular environment. The encoded protein is involved in the regulation of the microtubule filament system. It prevents assembly and promotes disassembly of microtubules | Microtubules |
| **FIGURE 5C: RNA PROCESSING, mRNA SPLICING** | | |
| TPR | **Translocated promoter region, nuclear basket protein***.* This large coiled-coil protein forms intranuclear filaments attached to the inner surface of nuclear pore complexes (NPCs). It is required for the nuclear export of mRNAs and some proteins | Nuclear export |
| SRSF11 | **Serine and arginine rich splicing factor 11.** This 54-kD nuclear protein contains an arginine/serine-rich region similar to segments found in pre-mRNA splicing factors. Although the function of this protein is not yet known, structure and immunolocalization data suggest that it may play a role in pre-mRNA processing | RNA processing |
| SRRM1 | **Serine and arginine repetitive matrix 1.** Chimeric RNAs generated by cis-splicing between adjacent genes (cis-SAGe) are increasingly recognized as a widespread phenomenon. The two factors SRRM1 and SF3B1 affect both cis-SAGe chimeras and other types of chimeric RNAs in a genome-wide fashion | RNA splicing |
| SF3B1 | **Splicing factor 3b subunit 1***.* This protein is the subunit 1 of the splicing factor 3b protein complex. Splicing factor 3b, together with splicing factor 3a and a 12S RNA unit, forms the U2 small nuclear ribonucleoproteins complex (U2 snRNP). The splicing factor 3b/3a complex binds pre-mRNA upstream of the intron's branch site in a sequence independent manner and may anchor the U2 snRNP to the pre-mRNA. Splicing factor 3b is also a component of the minor U12-type spliceosome | RNA splicing |
| HNRNPL | **Heterogeneous nuclear ribonucleoprotein L.** Heterogeneous nuclear RNAs (hnRNAs) which include mRNA precursors and mature mRNAs are associated with specific proteins to form heterogenous ribonucleoprotein (hnRNP) complexes. Heterogeneous nuclear ribonucleoprotein L is present in the nucleoplasm as part of the HNRP complex. HNRP proteins have also been identified outside of the nucleoplasm | Nuclear protein,  RNA,  nuclear export |
| SNIP1 | **Smad nuclear interacting protein 1.** This protein contains a coiled-coil motif and C-terminal forkhead-associated (FHA) domain. The encoded protein functions as a transcriptional coactivator that increases c-Myc activity and inhibits transforming growth factor beta (TGF-beta) and nuclear factor kappa-B (NF-kB) signaling. The encoded protein also regulates the stability of cyclin D1 mRNA and may play a role in cell proliferation and cancer progression | Transcription |
