# Supplementary material for: Biological characteristics of aging in human acute myeloid leukemia cells: the possible importance of aldehyde dehydrogenase, the cytoskeleton and altered transcriptional regulation
Source: Aging (Albany NY). 2020 Dec 20;12(24):24734–77. doi: 10.18632/aging.202361 (PMC7803495; doi:10.18632/aging.202361)
Supplement: Supplementary Table 8 [file aging-12-202361-s007.pdf]

SUPPLEMENTARY TABLE

Supplementary Table 8. Differentially regulated CDK1/2 phosphorylation sites identified by immunoprecipitation and LC-MS/MS analysis.

Protein	Phosphosites	FC high-risk/low-risk (LIMMA)
CDK1	T14	2.82
CDK2	T14	2.82
CDK1	T14 Y15	2.32
CDK2	T14 Y15	2.30

Phosphosites CDK1/2 T14 and Y15 were enriched using anti-pTyr-antibody immunoprecipitation and analyzed by LC-MS/MS. Significant phosphorylation differences between the high-risk and the low-risk groups were calculated with LIMMA statistics. The table below shows differentially regulated tyrosine-phosphorylated peptides with *Q*-values <0.05.
