# Supplementary material for: Biological characteristics of aging in human acute myeloid leukemia cells: the possible importance of aldehyde dehydrogenase, the cytoskeleton and altered transcriptional regulation
Source: Aging (Albany NY). 2020 Dec 20;12(24):24734–77. doi: 10.18632/aging.202361 (PMC7803495; doi:10.18632/aging.202361)
Supplement: Supplementary References [file aging-12-202361-s013.pdf]

SUPPLEMENTARY REFERENCES

1. López-Otín C, Blasco MA, Partridge L, Serrano M, Kroemer G. The hallmarks of aging. Cell. 2013; 153:1194–217.
https://doi.org/10.1016/j.cell.2013.05.039
PMID:23746838

2. Lee J, Yoon SR, Choi I, Jung H. Causes and mechanisms of hematopoietic stem cell aging. Int J Mol Sci. 2019; 20:1272.
https://doi.org/10.3390/ijms20061272
PMID:30871268

3. Zjablovskaja P, Florian MC. Acute myeloid leukemia: aging and epigenetics. Cancers (Basel). 2019; 12:103.
https://doi.org/10.3390/cancers12010103
PMID:31906064

4. Hu XY, Fang Q, Ma D, Jiang L, Yang Y, Sun J, Yang C, Wang JS. Aldehyde dehydrogenase 2 protects human umbilical vein endothelial cells against oxidative damage and increases endothelial nitric oxide production to reverse nitroglycerin tolerance. Genet Mol Res. 2016; 15.
https://doi.org/10.4238/gmr.15027822
PMID:27323160

5. Woolthuis CM, Park CY. Hematopoietic stem/ progenitor cell commitment to the megakaryocyte lineage. Blood. 2016; 127:1242–48.
https://doi.org/10.1182/blood-2015-07-607945
PMID:26787736

6. Aslam MA, Alemdehy MF, Pritchard CE, Song JY, Muhaimin FI, Wijdeven RH, Huijbers IJ, Neefjes J, Jacobs H. Towards an understanding of C9orf82 protein/CAAP1 function. PLoS One. 2019; 14:e0210526.
https://doi.org/10.1371/journal.pone.0210526
PMID:30629682

7. Rodríguez D, Bretones G, Quesada V, Villamor N, Arango JR, López-Guillermo A, Ramsay AJ, Baumann T, Quirós PM, Navarro A, Royo C, Martín-Subero JI, Campo E, López-Otín C. Mutations in CHD2 cause defective association with active chromatin in chronic lymphocytic leukemia. Blood. 2015; 126:195–202.
https://doi.org/10.1182/blood-2014-10-604959
PMID:26031915

8. Tanna CE, Goss LB, Ludwig CG, Chen PW. Arf GAPs as regulators of the actin cytoskeleton-an update. Int J Mol Sci. 2019; 20:442.
https://doi.org/10.3390/ijms20020442
PMID:30669557

9. van Gastel J, Boddaert J, Jushaj A, Premont RT, Luttrell LM, Janssens J, Martin B, Maudsley S. GIT2-a keystone in ageing and age-related disease. Ageing Res Rev. 2018; 43:46–63.
https://doi.org/10.1016/j.arr.2018.02.002
PMID:29452267

10. Buschman MD, Field SJ. MYO18A: an unusual myosin. Adv Biol Regul. 2018; 67:84–92.
https://doi.org/10.1016/j.jbior.2017.09.005
PMID:28942352

11. Kevenaar JT, Bianchi S, van Spronsen M, Olieric N, Lipka J, Frias CP, Mikhaylova M, Harterink M, Keijzer N, Wulf PS, Hilbert M, Kapitein LC, de Graaff E, et al. Kinesin-binding protein controls microtubule dynamics and cargo trafficking by regulating kinesin motor activity. Curr Biol. 2016; 26:849–61.
https://doi.org/10.1016/j.cub.2016.01.048
PMID:26948876

12. Malaby HL, Dumas ME, Ohi R, Stumpff J. Kinesin-binding protein ensures accurate chromosome segregation by buffering KIF18A and KIF15. J Cell Biol. 2019; 218:1218–34.
https://doi.org/10.1083/jcb.201806195
PMID:30709852

13. Zhang Q, Thakur C, Shi J, Sun J, Fu Y, Stemmer P, Chen F. New discoveries of mdig in the epigenetic regulation of cancers. Semin Cancer Biol. 2019; 57:27–35.
https://doi.org/10.1016/j.semcancer.2019.06.013
PMID:31276784

14. Chen M, Zhang H, Shi Z, Li Y, Zhang X, Gao Z, Zhou L, Ma J, Xu Q, Guan J, Cheng Y, Jiao S, Zhou Z. The MST4-MOB4 complex disrupts the MST1-MOB1 complex in the hippo-YAP pathway and plays a pro-oncogenic role in pancreatic cancer. J Biol Chem. 2018; 293:14455–69.
https://doi.org/10.1074/jbc.RA118.003279
PMID:30072378

15. Yeung YT, Guerrero-Castilla A, Cano M, Muñoz MF, Ayala A, Argüelles S. Dysregulation of the hippo pathway signaling in aging and cancer. Pharmacol Res. 2019; 143:151–65.
https://doi.org/10.1016/j.phrs.2019.03.018
PMID:30910741

16. Kimura I, Konishi M, Asaki T, Furukawa N, Ukai K, Mori M, Hirasawa A, Tsujimoto G, Ohta M, Itoh N, Fujimoto M. Neudesin, an extracellular heme-binding protein, suppresses adipogenesis in 3T3-L1 cells via the MAPK cascade. Biochem Biophys Res Commun. 2009; 381:75–80.

https://doi.org/10.1016/j.bbrc.2009.02.011
PMID:19351598

17. Puts GS, Leonard MK, Pamidimukkala NV, Snyder DE, Kaetzel DM. Nuclear functions of NME proteins. Lab Invest. 2018; 98:211–18. https://doi.org/10.1038/labinvest.2017.109 PMID:29058704

18. Singh KP, Bennett JA, Casado FL, Walrath JL, Welle SL, Gasiewicz TA. Loss of aryl hydrocarbon receptor promotes gene changes associated with premature hematopoietic stem cell exhaustion and development of a myeloproliferative disorder in aging mice. Stem Cells Dev. 2014; 23:95–106. https://doi.org/10.1089/scd.2013.0346 PMID:24138668

19. Stacpoole PW. The pyruvate dehydrogenase complex as a therapeutic target for age-related diseases. Aging Cell. 2012; 11:371–77. https://doi.org/10.1111/j.1474-9726.2012.00805.x PMID:22321732

20. Lillenes MS, Espeseth T, Støen M, Lundervold AJ, Frye SA, Rootwelt H, Reinvang I, Tønjum T. DNA base excision repair gene polymorphisms modulate human cognitive performance and decline during normal life span. Mech Ageing Dev. 2011; 132:449–58. https://doi.org/10.1016/j.mad.2011.08.002 PMID:21884718

21. Zhu S, Wang Z, Zhang Z, Wang J, Li Y, Yao L, Mei Q, Zhang W. PTPLAD2 is a tumor suppressor in esophageal squamous cell carcinogenesis. FEBS Lett. 2014; 588:981–89. https://doi.org/10.1016/j.febslet.2014.01.058 PMID:24530685

22. Camici GG, Savarese G, Akhmedov A, Lüscher TF. Molecular mechanism of endothelial and vascular aging: implications for cardiovascular disease. Eur Heart J. 2015; 36:3392–403. https://doi.org/10.1093/eurheartj/ehv587 PMID:26543043

23. Kopetz S, Shah AN, Gallick GE. Src continues aging: current and future clinical directions. Clin Cancer Res. 2007; 13:7232–36. https://doi.org/10.1158/1078-0432.CCR-07-1902 PMID:18094400

24. Krosl J, Mamo A, Chagraoui J, Wilhelm BT, Girard S, Louis I, Lessard J, Perreault C, Sauvageau G. A mutant allele of the Swi/Snf member BAF250a determines the pool size of fetal liver hemopoietic stem cell populations. Blood. 2010; 116:1678–84. https://doi.org/10.1182/blood-2010-03-273862 PMID:20522713

25. Ruiz-Lafuente N, Minguela A, Muro M, Parrado A. The role of DOCK10 in the regulation of the transcriptome and aging. Heliyon. 2019; 5:e01391. https://doi.org/10.1016/j.heliyon.2019.e01391 PMID:30963125

26. Watanabe R, Ui A, Kanno S, Ogiwara H, Nagase T, Kohno T, Yasui A. SWI/SNF factors required for cellular resistance to DNA damage include ARID1A and ARID1B and show interdependent protein stability. Cancer Res. 2014; 74:2465–75. https://doi.org/10.1158/0008-5472.CAN-13-3608 PMID:24788099

27. Kadono M, Kanai A, Nagamachi A, Shinriki S, Kawata J, Iwato K, Kyo T, Oshima K, Yokoyama A, Kawamura T, Nagase R, Inoue D, Kitamura T, et al. Biological implications of somatic DDX41 p.R525H mutation in acute myeloid leukemia. Exp Hematol. 2016; 44:745–54.e4. https://doi.org/10.1016/j.exphem.2016.04.017 PMID:27174803

28. Makishima H. [Sequential acquisition of mutations in myelodysplastic syndromes]. Rinsho Ketsueki. 2017; 58:1828–37. https://doi.org/10.11406/rinketsu.58.1828 PMID:28978821

29. Geiger H, Zheng Y. Cdc42 and aging of hematopoietic stem cells. Curr Opin Hematol. 2013; 20:295–300. https://doi.org/10.1097/MOH.0b013e3283615aba PMID:23615056

30. Hao YH, Doyle JM, Ramanathan S, Gomez TS, Jia D, Xu M, Chen ZJ, Billadeau DD, Rosen MK, Potts PR. Regulation of WASH-dependent actin polymerization and protein trafficking by ubiquitination. Cell. 2013; 152:1051–64. https://doi.org/10.1016/j.cell.2013.01.051 PMID:23452853

31. Singh J, Kumar S, Krishna CV, Rattan S. Aging-associated oxidative stress leads to decrease in IAS tone via RhoA/ROCK downregulation. Am J Physiol Gastrointest Liver Physiol. 2014; 306:G983–91. https://doi.org/10.1152/ajpgi.00087.2014 PMID:24742984

32. Tapia PC. RhoA, rho kinase, JAK2, and STAT3 may be the intracellular determinants of longevity implicated in the progeric influence of obesity: insulin, IGF-1, and leptin may all conspire to promote stem cell exhaustion. Med Hypotheses. 2006; 66:570–76. https://doi.org/10.1016/j.mehy.2005.09.008 PMID:16226846

33. Florian MC, Dörr K, Niebel A, Daria D, Schrezenmeier H, Rojewski M, Filippi MD, Hasenberg A, Gunzer M, Scharffetter-Kochanek K, Zheng Y, Geiger H. Cdc42

activity regulates hematopoietic stem cell aging and rejuvenation. Cell Stem Cell. 2012; 10:520–30. https://doi.org/10.1016/j.stem.2012.04.007 PMID:22560076

34. Florian MC, Klose M, Sacma M, Jablanovic J, Knudson L, Nattamai KJ, Marka G, Vollmer A, Soller K, Sakk V, Cabezas-Wallscheid N, Zheng Y, Mulaw MA, et al. Aging alters the epigenetic asymmetry of HSC division. PLoS Biol. 2018; 16:e2003389. https://doi.org/10.1371/journal.pbio.2003389 PMID:30235201

35. Ramalho-Oliveira R, Oliveira-Vieira B, Viola JP. IRF2BP2: a new player in the regulation of cell homeostasis. J Leukoc Biol. 2019; 106:717–23. https://doi.org/10.1002/JLB.MR1218-507R PMID:31022319

36. Brauchle M, Yao Z, Arora R, Thigale S, Clay I, Inverardi B, Fletcher J, Taslimi P, Acker MG, Gerrits B, Voshol J, Bauer A, Schübeler D, et al. Protein complex interactor analysis and differential activity of KDM3 subfamily members towards H3K9 methylation. PLoS One. 2013; 8:e60549. https://doi.org/10.1371/journal.pone.0060549 PMID:23593242

37. Li J, Yu B, Deng P, Cheng Y, Yu Y, Kevork K, Ramadoss S, Ding X, Li X, Wang CY. Author correction: KDM3 epigenetically controls tumorigenic potentials of human colorectal cancer stem cells through Wnt/β-catenin signalling. Nat Commun. 2019; 10:5020. https://doi.org/10.1038/s41467-019-12878-z PMID:31685815

38. Lai WF, Wong WT. Roles of the actin cytoskeleton in aging and age-associated diseases. Ageing Res Rev. 2020; 58:101021. https://doi.org/10.1016/j.arr.2020.101021 PMID:31968269

39. Martin-Rendon E, Hale SJ, Ryan D, Baban D, Forde SP, Roubelakis M, Sweeney D, Moukayed M, Harris AL, Davies K, Watt SM. Transcriptional profiling of human cord blood CD133+ and cultured bone marrow mesenchymal stem cells in response to hypoxia. Stem Cells. 2007; 25:1003–12. https://doi.org/10.1634/stemcells.2006-0398 PMID:17185612

40. Bocquet N, Bizard AH, Abdulrahman W, Larsen NB, Faty M, Cavadini S, Bunker RD, Kowalczykowski SC, Cejka P, Hickson ID, Thomä NH. Structural and mechanistic insight into holliday-junction dissolution by topoisomerase IIIα and RMI1. Nat Struct Mol Biol. 2014; 21:261–68. https://doi.org/10.1038/nsmb.2775 PMID:24509834

41. Bounaix Morand du Puch C, Barbier E, Kraut A, Couté Y, Fuchs J, Buhot A, Livache T, Sève M, Favier A, Douki T, Gasparutto D, Sauvaigo S, Breton J. TOX4 and its binding partners recognize DNA adducts generated by platinum anticancer drugs. Arch Biochem Biophys. 2011; 507:296–303. https://doi.org/10.1016/j.abb.2010.12.021 PMID:21184731

42. Vanheer L, Song J, De Geest N, Janiszewski A, Talon I, Provenzano C, Oh T, Chappell J, Pasque V. Tox4 modulates cell fate reprogramming. J Cell Sci. 2019; 132:jcs232223. https://doi.org/10.1242/jcs.232223 PMID:31519808

43. Barbosa K, Li S, Adams PD, Deshpande AJ. The role of TP53 in acute myeloid leukemia: challenges and opportunities. Genes Chromosomes Cancer. 2019; 58:875–88. https://doi.org/10.1002/gcc.22796 PMID:31393631

44. Ou HL, Schumacher B. DNA damage responses and p53 in the aging process. Blood. 2018; 131:488–95. https://doi.org/10.1182/blood-2017-07-746396 PMID:29141944

45. Wu D, Prives C. Relevance of the p53-MDM2 axis to aging. Cell Death Differ. 2018; 25:169–79. https://doi.org/10.1038/cdd.2017.187 PMID:29192902

46. Chen J, Wang A, Chen Q. SirT3 and p53 deacetylation in aging and cancer. J Cell Physiol. 2017; 232:2308–11. https://doi.org/10.1002/jcp.25669 PMID:27791271

47. Rufini A, Tucci P, Celardo I, Melino G. Senescence and aging: the critical roles of p53. Oncogene. 2013; 32:5129–43. https://doi.org/10.1038/onc.2012.640 PMID:23416979

48. Kaiser RW, Ignarski M, Van Nostrand EL, Frese CK, Jain M, Cukoski S, Heinen H, Schaechter M, Seufert L, Bunte K, Frommolt P, Keller P, Helm M, et al. A protein-RNA interaction atlas of the ribosome biogenesis factor AATF. Sci Rep. 2019; 9:11071. https://doi.org/10.1038/s41598-019-47552-3 PMID:31363146

49. Koo SJ, Fernández-Montalván AE, Badock V, Ott CJ, Holton SJ, von Ahsen O, Toedling J, Vittori S, Bradner JE, Gorjánácz M. ATAD2 is an epigenetic reader of newly synthesized histone marks during DNA replication. Oncotarget. 2016; 7:70323–35. https://doi.org/10.18632/oncotarget.11855 PMID:27612420

50. Morozumi Y, Boussouar F, Tan M, Chaikuad A, Jamshidikia M, Colak G, He H, Nie L, Petosa C, de Dieuleveult M, Curtet S, Vitte AL, Rabatel C, et al. Atad2 is a generalist facilitator of chromatin dynamics in embryonic stem cells. J Mol Cell Biol. 2016; 8:349–62. https://doi.org/10.1093/jmcb/mjv060 PMID:26459632

51. Shao AW, Sun H, Geng Y, Peng Q, Wang P, Chen J, Xiong T, Cao R, Tang J. Bclaf1 is an important NF-κB signaling transducer and C/EBPβ regulator in DNA damage-induced senescence. Cell Death Differ. 2016; 23:865–75. https://doi.org/10.1038/cdd.2015.150 PMID:26794446

52. Chae U, Park JW, Lee SR, Lee HJ, Lee HS, Lee DS. Reactive oxygen species-mediated senescence is accelerated by inhibiting Cdk2 in Idh2-deficient conditions. Aging (Albany NY). 2019; 11:7242–56. https://doi.org/10.18632/aging.102259 PMID:31503005

53. Heshmati Y, Türköz G, Harisankar A, Kharazi S, Boström J, Dolatabadi EK, Krstic A, Chang D, Månsson R, Altun M, Qian H, Walfridsson J. The chromatin-remodeling factor CHD4 is required for maintenance of childhood acute myeloid leukemia. Haematologica. 2018; 103:1169–81. https://doi.org/10.3324/haematol.2017.183970 PMID:29599201

54. Sperlazza J, Rahmani M, Beckta J, Aust M, Hawkins E, Wang SZ, Zu Zhu S, Podder S, Dumur C, Archer K, Grant S, Ginder GD. Depletion of the chromatin remodeler CHD4 sensitizes AML blasts to genotoxic agents and reduces tumor formation. Blood. 2015; 126:1462–72. https://doi.org/10.1182/blood-2015-03-631606 PMID:26265695

55. Ooga M, Funaya S, Hashioka Y, Fujii W, Naito K, Suzuki MG, Aoki F. Chd9 mediates highly loosened chromatin structure in growing mouse oocytes. Biochem Biophys Res Commun. 2018; 500:583–88. https://doi.org/10.1016/j.bbrc.2018.04.105 PMID:29665362

56. Unlu I, Lu Y, Wang X. The cyclic phosphodiesterase CNP and RNA cyclase RtcA fine-tune noncanonical XBP1 splicing during ER stress. J Biol Chem. 2018; 293:19365–76. https://doi.org/10.1074/jbc.RA118.004872 PMID:30355738

57. Xie J, de Souza Alves V, von der Haar T, O'Keefe L, Lenchine RV, Jensen KB, Liu R, Coldwell MJ, Wang X, Proud CG. Regulation of the elongation phase of protein synthesis enhances translation accuracy and modulates lifespan. Curr Biol. 2019; 29:737–49.e5. https://doi.org/10.1016/j.cub.2019.01.029 PMID:30773367

58. Wen F, Zhou R, Shen A, Choi A, Uribe D, Shi J. The tumor suppressive role of eIF3f and its function in translation inhibition and rRNA degradation. PLoS One. 2012; 7:e34194. https://doi.org/10.1371/journal.pone.0034194 PMID:22457825

59. Schauder CM, Wu X, Saheki Y, Narayanaswamy P, Torta F, Wenk MR, De Camilli P, Reinisch KM. Structure of a lipid-bound extended synaptotagmin indicates a role in lipid transfer. Nature. 2014; 510:552–55. https://doi.org/10.1038/nature13269 PMID:24847877

60. Yang Q, Wang J, Zhong P, Mou T, Hua H, Liu P, Xie F. The clinical prognostic value of lncRNA FAM83H-AS1 in cancer patients: a meta-analysis. Cancer Cell Int. 2020; 20:72. https://doi.org/10.1186/s12935-020-1148-8 PMID:32165862

61. Lin MF, Yang YF, Peng ZP, Zhang MF, Liang JY, Chen W, Liu XH, Zheng YL. FOXK2, regulted by miR-1271-5p, promotes cell growth and indicates unfavorable prognosis in hepatocellular carcinoma. Int J Biochem Cell Biol. 2017; 88:155–61. https://doi.org/10.1016/j.biocel.2017.05.019 PMID:28506857

62. Liu X, Wei X, Niu W, Wang D, Wang B, Zhuang H. Downregulation of FOXK2 is associated with poor prognosis in patients with gastric cancer. Mol Med Rep. 2018; 18:4356–64. https://doi.org/10.3892/mmr.2018.9466 PMID:30221666

63. Nestal de Moraes G, Carneiro LD, Maia RC, Lam EW, Sharrocks AD. FOXK2 transcription factor and its emerging roles in cancer. Cancers (Basel). 2019; 11:393. https://doi.org/10.3390/cancers11030393 PMID:30897782

64. Balboula AZ, Nguyen AL, Gentilello AS, Quartuccio SM, Drutovic D, Solc P, Schindler K. Haspin kinase regulates microtubule-organizing center clustering and stability through aurora kinase C in mouse oocytes. J Cell Sci. 2016; 129:3648–60. https://doi.org/10.1242/jcs.189340 PMID:27562071

65. Liang C, Chen Q, Yi Q, Zhang M, Yan H, Zhang B, Zhou L, Zhang Z, Qi F, Ye S, Wang F. A kinase-dependent role for haspin in antagonizing wapl and protecting mitotic centromere cohesion. EMBO Rep. 2018; 19:43–56. https://doi.org/10.15252/embr.201744737 PMID:29138236

66. Maiolica A, de Medina-Redondo M, Schoof EM, Chaikuad A, Villa F, Gatti M, Jeganathan S, Lou HJ, Novy K, Hauri S, Toprak UH, Herzog F, Meraldi P, et al. Modulation of the chromatin phosphoproteome by the haspin protein kinase. Mol Cell Proteomics. 2014; 13:1724–40. https://doi.org/10.1074/mcp.M113.034819 PMID:24732914

67. Short B. GTSE1 leads cancer cells into CIN. J Cell Biol. 2016; 215:593.

https://doi.org/10.1083/jcb.2155if PMID:27888203

68. Thalappilly S, Feng X, Pastyryeva S, Suzuki K, Muruve D, Larocque D, Richard S, Truss M, von Deimling A, Riabowol K, Tallen G. The p53 tumor suppressor is stabilized by inhibitor of growth 1 (ING1) by blocking polyubiquitination. PLoS One. 2011; 6:e21065. https://doi.org/10.1371/journal.pone.0021065 PMID:21731648

69. Schmidt K, Zhang Q, Tasdogan A, Petzold A, Dahl A, Arneth BM, Slany R, Fehling HJ, Kranz A, Stewart AF, Anastassiadis K. The H3K4 methyltransferase Setd1b is essential for hematopoietic stem and progenitor cell homeostasis in mice. Elife. 2018; 7:e27157. https://doi.org/10.7554/eLife.27157 PMID:29916805

70. Cornell RB, Ridgway ND. CTP:phosphocholine cytidylyltransferase: function, regulation, and structure of an amphitropic enzyme required for membrane biogenesis. Prog Lipid Res. 2015; 59:147–71. https://doi.org/10.1016/j.plipres.2015.07.001 PMID:26165797

71. Ganduri S, Lue NF. STN1-POLA2 interaction provides a basis for primase-pol α stimulation by human STN1. Nucleic Acids Res. 2017; 45:9455–66. https://doi.org/10.1093/nar/gkx621 PMID:28934486

72. Szlavicz E, Szabo K, Groma G, Bata-Csorgo Z, Pagani F, Kemeny L, Szell M. Splicing factors differentially expressed in psoriasis alter mRNA maturation of disease-associated EDA+ fibronectin. Mol Cell Biochem. 2017; 436:189–99. https://doi.org/10.1007/s11010-017-3090-1 PMID:28589370

73. Qiu R, Zhang J, Xiang X. The splicing-factor Prp40 affects dynein-dynactin function in Aspergillus nidulans. Mol Biol Cell. 2020; 31:1289–301. https://doi.org/10.1091/mbc.E20-03-0166 PMID:32267207

74. Ni Z, Xu C, Guo X, Hunter GO, Kuznetsova OV, Tempel W, Marcon E, Zhong G, Guo H, Kuo WW, Li J, Young P, Olsen JB, et al. RPRD1A and RPRD1B are human RNA polymerase II C-terminal domain scaffolds for Ser5 dephosphorylation. Nat Struct Mol Biol. 2014; 21:686–95. https://doi.org/10.1038/nsmb.2853 PMID:24997600

75. Alsarraj J, Faraji F, Geiger TR, Mattaini KR, Williams M, Wu J, Ha NH, Merlino T, Walker RC, Bosley AD, Xiao Z, Andresson T, Esposito D, et al. BRD4 short isoform interacts with RRP1B, SIPA1 and components of the LINC complex at the inner face of the nuclear membrane. PLoS One. 2013; 8:e80746. https://doi.org/10.1371/journal.pone.0080746 PMID:24260471

76. Lee M, Dworkin AM, Gildea D, Trivedi NS, Moorhead GB, Crawford NP, and NISC Comparative Sequencing Program. RRP1B is a metastasis modifier that regulates the expression of alternative mRNA isoforms through interactions with SRSF1. Oncogene. 2014; 33:1818–27. https://doi.org/10.1038/onc.2013.133 PMID:23604122

77. Maiese K. Disease onset and aging in the world of circular RNAs. J Transl Sci. 2016; 2:327–29. https://doi.org/10.15761/jts.1000158 PMID:27642518

78. Gérus M, Bonnart C, Caizergues-Ferrer M, Henry Y, Henras AK. Evolutionarily conserved function of RRP36 in early cleavages of the pre-rRNA and production of the 40S ribosomal subunit. Mol Cell Biol. 2010; 30:1130–44. https://doi.org/10.1128/MCB.00999-09 PMID:20038530

79. Dash BP, Schnöder TM, Kathner C, Mohr J, Weinert S, Herzog C, Godavarthy PS, Zanetti C, Perner F, Braun-Dullaeus R, Hartleben B, Huber TB, Walz G, et al. Diverging impact of cell fate determinants scrib and Llgl1 on adhesion and migration of hematopoietic stem cells. J Cancer Res Clin Oncol. 2018; 144:1933–44. https://doi.org/10.1007/s00432-018-2724-3 PMID:30083817

80. Chwalenia K, Qin F, Singh S, Li H. A cell-based splicing reporter system to identify regulators of cis-splicing between adjacent genes. Nucleic Acids Res. 2019; 47:e24. https://doi.org/10.1093/nar/gky1288 PMID:30590765

81. Zanini IM, Soneson C, Lorenzi LE, Azzalin CM. Human cactin interacts with DHX8 and SRRM2 to assure efficient pre-mRNA splicing and sister chromatid cohesion. J Cell Sci. 2017; 130:767–78. https://doi.org/10.1242/jcs.194068 PMID:28062851

82. Bhullar J, Sollars VE. YBX1 expression and function in early hematopoiesis and leukemic cells. Immunogenetics. 2011; 63:337–50. https://doi.org/10.1007/s00251-011-0517-9 PMID:21369783

83. Knuckles P, Lence T, Haussmann IU, Jacob D, Kreim N, Carl SH, Masiello I, Hares T, Villaseñor R, Hess D, Andrade-Navarro MA, Biggiogera M, Helm M, et al. Zc3h13/flacc is required for adenosine methylation by bridging the mRNA-binding factor Rbm15/Spenito to the m^6A machinery component Wtap/Fl(2)d. Genes Dev. 2018; 32:415–29. https://doi.org/10.1101/gad.309146.117 PMID:29535189

84. Wen J, Lv R, Ma H, Shen H, He C, Wang J, Jiao F, Liu H, Yang P, Tan L, Lan F, Shi YG, He C, et al. Zc3h13 regulates nuclear RNA m^6A methylation and mouse embryonic stem cell self-renewal. Mol Cell. 2018; 69:1028–38.e6. https://doi.org/10.1016/j.molcel.2018.02.015 PMID:29547716
