# Supplementary material for: Biological characteristics of aging in human acute myeloid leukemia cells: the possible importance of aldehyde dehydrogenase, the cytoskeleton and altered transcriptional regulation
Source: Aging (Albany NY). 2020 Dec 20;12(24):24734–77. doi: 10.18632/aging.202361 (PMC7803495; doi:10.18632/aging.202361)
Supplement: Supplementary Data Analysis [file aging-12-202361-s014.pdf]

SUPPLEMENTARY DATA ANALYSIS

Supplementary Analysis. The proliferative *in vitro* responsiveness of primary human AML cells derived from 63 consecutive patients.

The proliferative responsiveness was tested by a [^{3}H]-thymidine incorporation assay prepared in serum-free medium as described in a previous article [1]. All growth factors were tested at a final concentration of 20 ng/ml. During *in vitro* culture AML cells undergo spontaneous apoptosis. We tested [^{3}H]-thymidine by adding [^{3}H]-thymidine after six days of culture, and the cultures were harvested 24 hours later. Thus, the proliferative responsiveness reflects the characteristics for a subset of cells within the hierarchically organized AML cell population that is capable of surviving for at least six days and still be able to show detectable proliferation.

The results are presented as number of patients with detectable proliferation, this was defined as a [^{3}H]-thymidine incorporation corresponding to >1000 cpm. Patient characteristics are described in the first table

REFERENCE

1. Bruserud Ø, Ryningen A, Olsnes AM, Stordrange L, Øyan AM, Kalland KH, Gjertsen BT. Subclassification of patients with acute myelogenous leukemia based on chemokine responsiveness and constitutive chemokine release by their leukemic cells. Haematologica. 2007; 92:332–41. https://doi.org/10.3324/haematol.10148 PMID:17339182

Supplementary Analysis Table 1. Characteristics of 63 consecutive patients used in the study of possible associations between age and differentiation.

**Younger patients (<65 years of age, n=38)**				**Elderly patients (>65 years of age, n=25)**			
Male/female 20/18		*Karyotype*		Male/female 15/10		*Karyotype*	
		Favorable	2			Favorable	1
FAB classification		Adverse	4	*FAB classification*		Adverse	4
M0	3	Intermediate	10	M0	5	Intermediate	2
M1	6	Normal	18	M1	5	Normal	3
M2	10	Not tested	4	M2	7	Not tested	15
**M4**	**11**			**M4**	**5**		
**M5**	**7**	*FLT3 abnormalities*		**M5**	**3**	*FLT3 abnormalities*	
M6	1	ITD	11	M6	0	ITD	8
		D835	2			D835	2
		WT	18			WT	12
		Not tested	7			Not tested	3
CD34 expression	18			*CD34 expression*	11		
de novo	31			*de novo*	16		
Predisposition		*NPM1 abnormalities*		*Predisposition*		*NPM1 abnormalities*	
MDS	1	Insertion	8	MDS	6	Insertion	2
MPN	1	WT	14	MPN	1	WT	20
Chemotherapy	5	Not tested	16	Chemotherapy	2	Not tested	3

COMMENTS: We investigated 63 consecutive patients with high peripheral blood blast counts (REK 1759/2015, REK 305/2017). Enriched AML cells could thereby be prepared by density gradient separation alone (see Material and methods in the main text). CD34 positivity was defined as at least 20% positive cells in flow cytometric analysis compared with the negative isotype control. The data above are presented as the number of patients. Red font indicates statistical difference between the two age groups.

In contrast to our main patient cohort that included only high- and low-risk patients, this second cohort included consecutive patients (and thereby unselected) patients and also patients with intermediate prognosis, i.e. normal or intermediate karyotype that constitutes approximately 60% of all patients in our biobank.

Supplementary Analysis Table 2. Proliferative responsiveness of the AML cells from 63 patients.

**Younger patients (<65 years of age, n=38)**		**Elderly patients (>65 years of age, n=25)**	
Culture condition	*Number of responders*	*Culture condition*	*Number of responders*
Medium alone	15	Medium alone	7
IL1β	27	IL1β	16
IL3	28	IL3	20
SCF	27	SCF	19
FLT3-ligand	24	FLT3-ligand	15
GM-CSF	27	GM-CSF	20
G-CSF	25	G-CSF	20
M-CSF	17	M-CSF	12
Thrombopoietin	15	Thrombopoietin	12
**Patients without morphological signs of monocytic differentiation (n=36)**		**Patients with morphological signs of monocytic differentiation (FAB-M4/M5, n=27)**	
Culture condition	*Number of responders*	*Culture condition*	*Number of responders*
Medium alone	14	Medium alone	8
IL1β	24	IL1β	16
IL3	30	IL3	18
SCF	29	SCF	17
FLT3-ligand	27	FLT3-ligand	12
GM-CSF	29	GM-CSF	18
G-CSF	29	G-CSF	16
M-CSF	16	M-CSF	13
Thrombopoietin	16	Thrombopoietin	11

COMMENTS: In this analysis we compared morphology, CD34 expression and proliferative responsiveness in the presence of several growth factors for a group of consecutive patients. It can be seen from Supplementary Table 1 and Table 1 that morphological signs of monocytic differentiation (i.e. FAB M4/M5) were more common among low-risk (i.e. relatively young) than among high-risk patients (Fisher's exact test, *P*= 0.039). We therefore investigated whether there were any significant associations between age (i.e. comparing patients above and below 65 years of age) and differentiation in an additional cohort that included 63 consecutive/unselected AML patients. None of the patients from the high-/low-risk groups (see Supplementary Table 1) was included among these 63 patients. Morphological signs of monocytic differentiation were significantly more frequent among younger patients also in this cohort (Fisher's exact test, *P*= 0.0334). However, patient age showed no significant associations with expression of the CD34 stem cell marker, molecular differentiation markers (i.e. CD13, CD14, CD15, CD33; data not shown) or the proliferative responsiveness to hematopoietic growth factors with (G-CSF, M-CSF, thrombopoietin) or without (IL1β, IL3, SCF, FLT3-ligand) lineage associations. The proliferative responsiveness of patients with and without morphological signs of differentiation did not differ significantly either.

CONCLUSION: Although we observed a difference in the expression of mitosis/proliferation regulatory proteins when comparing high-risk and low-risk AML patients, we could not find any evidence for a general association between differentiation status and proliferative capacity of primary human AML cells when investigating this consecutive group of patients.

Abbreviations: FAB, French-American-British; G/GM/M-CSF, granulocyte/granulocyte-monocyte/monocyte colony-stimulating factor; IL, interleukin; ITD, internal tandem duplication; MDS, myelodysplastic syndrome; MPN, myeloproliferative neoplasia; SCF, stem cell factor: WT, wild –type.
